# Gene expression network analysis of lymph node involvement in colon cancer identifies AHSA2, CDK10, and CWC22 as possible prognostic markers

**DOI:** 10.1038/s41598-020-63806-x

**Published:** 2020-04-28

**Authors:** Sung Won Han, Ji Young Ahn, Soobin Lee, Young Seon Noh, Hee Chan Jung, Min Hyung Lee, Hae Jun Park, Hoon Jai Chun, Seong Ji Choi, Eun Sun Kim, Ji-Yun Lee

**Affiliations:** 1 School of Industrial Management Engineering, Korea University, Seoul, 02841 Republic of Korea; 2 Department of Internal Medicine, Eulji University College of Medicine 68 Hangeulbiseok-ro, Nowon-gu, Seoul 01830 Republic of Korea; 3 Department of Internal Medicine, Korea University College of Medicine, Seoul, 02841 Republic of Korea; 4 Department of Pathology, Korea University College of Medicine, Seoul, 02841 Republic of Korea

**Keywords:** Colorectal cancer, Scientific data

## Abstract

Colon cancer has been well studied using a variety of molecular techniques, including whole genome sequencing. However, genetic markers that could be used to predict lymph node (LN) involvement, which is the most important prognostic factor for colon cancer, have not been identified. In the present study, we compared LN(+) and LN(−) colon cancer patients using differential gene expression and network analysis. Colon cancer gene expression data were obtained from the Cancer Genome Atlas and divided into two groups, LN(+) and LN(−). Gene expression networks were constructed using LASSO (Least Absolute Shrinkage and Selection Operator) regression. We identified hub genes, such as APBB1, AHSA2, ZNF767, and JAK2, that were highly differentially expressed. Survival analysis using selected hub genes, such as AHSA2, CDK10, and CWC22, showed that their expression levels were significantly associated with the survival rate of colon cancer patients, which indicates their possible use as prognostic markers. In addition, protein-protein interaction network, GO enrichment, and KEGG pathway analysis were performed with selected hub genes from each group to investigate the regulatory relationships between hub genes and LN involvement in colon cancer; these analyses revealed differences between the LN(−) and LN(+) groups. Our network analysis may help narrow down the search for novel candidate genes for the treatment of colon cancer, in addition to improving our understanding of the biological processes underlying LN involvement. All R implementation codes are available at journal website as Supplementary Materials.

## Introduction

Colon cancer is a disease that worldwide has both a high incidence and prevalence, such that its impact on human health is well recognized^1^. Unlike other cancers, the progression of colon cancer has been well understood since 1988 when Vogelstein *et al*.^2^ described the process of the development of an adenoma into cancer, suggesting that the development of cancer is a systematic process. With the improvement of molecular technologies, our understanding of the molecular mechanisms by which genetic changes, such as alterations in DNA, that lead a normal mucosa to become colon cancer has deepened^3^. Increasingly, various mechanisms related to colon carcinogenesis have been revealed, such as chromosomal instability, microsatellite instability (MSI), non-MSI hypermutability, aberrant DNA methylation, global DNA hypomethylation, as well as DNA mutation^4,5^.

Most molecular and genetic studies in colon cancer have focused on tumorigenesis and have revealed the existence of several important genes and pathways that can lead to the early diagnosis of colon cancer. Nevertheless, the most important prognostic factor in colon cancer remains the tumor node metastasis (TNM) stage^6^. Stage II and III cancers are mainly differentiated based on nodal (N) stage, indicating the importance of lymph node (LN) involvement in prognosis. Currently, the N stage is decided by the pathologist after examination of LNs removed during surgery. However, sometimes patients are under-staged because of an inadequate number of LNs retrieved during surgery; these under-staged patients lose their opportunity for adjuvant chemotherapy resulting in a higher risk of tumor recurrence^7^. This makes the prediction or diagnosis of lymph node involvement extremely important for patient care.

To assess the diagnostic and/or prognostic possibilities regarding LN involvement in colon cancer, we analyzed and compared gene network of the gene expression in LN (+) colon cancer and LN (−) colon cancer, and identify significantly differing gene(s) from the gene networks using the Cancer Genome Atlas (TCGA) database (https://cancergenome.nih.gov/). A conventional and widely used method of gene expression profiling is the differential expression of genes (DEG). However, a DEG analysis has the evident limitation of being unable to identify interactions between multiple genes, and also the inability to ensure the involvement of the most significantly differentially expressed genes with the disease^8,9^. To overcome these limitations, we combined a network analysis referred to as the degree of centrality method with the DEG analysis^10^. The degree of centrality method is one of the simplest methods to measure the degree of the edge between a hub gene constituting a network and other genes directly connected to the hub using the number of adjacent hub genes. It is possible to identify very important hub genes or connector genes in terms of degree on the network by a degree centrality analysis, which detects how far genes are located from the center or genes acting as connectors or hubs in a network.

In addition, the protein-protein interaction network, GO enrichment, and KEGG pathways were searched using the selected hub genes from each group to better understand the regulatory relationship between hub genes and the biological events driving LN involvement in colon cancer.

## Methods

### Data collection and characterization

The RNA sequencing data set from colon cancer patients was obtained from Fire Browse (Version 1.1.35), which provides the TCGA data sets (accessed in Feb 2017)^11^. These data provide RNA sequencing V2 expression levels values for each gene. We reviewed and characterized the clinical information from the collected data set and divided them into two groups designated as LN (−) or (+) (Table 1). The clinical data include age, gender, TNM stage, and radiation therapy status etc. Out of a total of 395 colorectal cancer patients, there were 179 LN(+) samples, and 216 LN(−) samples. The average age at diagnosis was 64.54 years-old in the LN(+) colorectal cancer group, whereas it was 68.29 years-old in the LN(−) colorectal cancer group.

**Table 1:** Clinical characteristics of the LN(+) and LN(−) patients group with colorectal cancer collected from TCGA.

COAD		Lymph node		Total	LN(+) vs. LN(−) p-value
		Positive	Negative		
		Value (%)	Value (%)	Value(%)	
		179 (45)	216(55)	395(100)	
Age	mean (SD)	64.54 ± 13.4	68.29 ± 12.37	66.58 ± 13	0.005
	median	66	70	68	0.006
Gender	FEMALE	89(50)	90(42)	179(45)	0.134
	MALE	90(50)	126(58)	216(55)	0.134
	NA	0	0	0	
Status	Alive	123(69)	185(86)	308(78)	8.83E-05
	Dead	56(31)	31(14)	87(22)	8.83E-05
	NA	0	0	0	
Race	WHITE	87(74)	93(76)	180(75)	0.014
	BLACK OR AFRICAN AMERICAN	29(25)	19(16)	48(20)	0.014
	ASIAN	1(1)	10(8)	11(5)	0.014
	AMERICAN INDIAN OR ALASKA NATIVE	1(1)	0(0)	1(0)	0.014
	NA	61	94	155	
Radiation Therapy	NO	148(97)	178(98)	326(98)	0.549
	YES	5(3)	3(2)	8(2)	0.549
	NA	26	35	0	
Stage	I	0(0)	66(31)	66(17)	3.30E-79
	II	0(0)	142(66)	142(36)	3.30E-79
	III	125(70)	0(0)	125(32)	3.30E-79
	IV	54(30)	8(4)	62(16)	3.30E-79
	NA	0	0	0	
T stage	t1	1(1)	8(4)	9(2)	7.16E-16
	t2	9(5)	57(26)	66(17)	7.16E-16
	t3	129(72)	149(69)	278(70)	7.16E-16
	t4	40(22)	1(0)	41(10)	7.16E-16
	tis	0(0)	0	1(0)	7.16E-16
	NA	0	0	0	
N stage	n0	0(0)	216(100)	216(55)	1.69E-86
	n1	101(56)	0(0)	101(26)	1.69E-86
	n2	78(44)	0(0)	78(20)	1.69E-86
	NA	0	0	0	
M stage	m0	102(57)	207(96)	309(78)	2.50E-20
	m1	54(30)	8(4)	62(16)	2.50E-20
	mx	23(13)	0(0)	23(6)	2.50E-20
	NA	0	1	0	

### DEG and network analysis

The RNA sequencing data from the LN (+) and (−) groups were pre-processed as follows: A total of 17,009 genes was selected after removing genes where the expression value were assigned as “0” in more than half of the samples. The expression value of each gene was converted to log2 scale and standardized for DEG analysis. Statistically significant differences in the gene expression levels of the LN(+) and LN(−) colorectal cancer samples were analyzed using a *t*-test. A total of 17,009 selected genes, which were used for the DEG analysis, were used to evaluate the gene networks in the LN (−) and LN (+) colorectal cancer groups based on a network estimation method. The network estimation method finds probabilistic neighbors (the edge gene in a network) for each gene (the node within the network) using a LASSO regression. The penalty parameter value in LASSO was obtained using the formula proposed by Meinshausen and Buhlmann, and it satisfied the asymptotic property^12^. The LASSO regression was performed using the R package glmnet. The hub genes in the network of LN(+) and LN(−) colorectal cancer groups were analyzed by the degree of centrality using R programming. For further network analysis, hub genes with a less than 20% coefficient of variation (CV) were selected from both groups. A CV cutoff of 20% was chosen because in general a CV of less than 20%, and not more than 30%, is considered to be an indicator of the reliability or measurement error in any analysis^13^.

### Survival analysis

Kaplan-Meier plots were used to estimate survival rates^14^. A multivariate analysis was used to evaluate whether the groups clustered by the expression levels of selected genes were an independent prognostic factor for overall survival. A P value less than 0.05 was considered statistically significant.

### Protein-protein interaction network, Gene ontology (GO), and KEGG pathway enrichment analysis

The protein-protein interaction network, gene ontology (GO), and KEGG pathway enrichment were searched using 353 hub genes from the LN(−) group and 240 hub genes from the LN(−) group. The protein-protein interaction network was analyzed using the Tool for the Retrieval of Interacting Genes/Proteins (STRING), and interactions with a confidence score of more than 0.95 were selected (https://string-db.org/). GO enrichment analysis was performed using DAVID Bioinformatics Resources (version 7.0). KEGG pathway enrichment analysis was performed using KEGG Mapper (https://www.genome.jp/kegg/tool/map_pathway2.html).

## Results

### DEG analysis

To analyze the DEG levels between the LN(+) and LN(−) groups, we extracted the 1918 genes with p-value < 0.005 and calculated the median gene expression levels using a Wilcoxon-test. The relative gene expression levels between the LN(+) to LN(−) groups were subdivided into upregulated and downregulated (Supplementary Table 1), which were plotted into heat map (Supplementary Fig. 1). The genes INTS10, AGPAT5, NAT1, MINPPP1, EFR1, and PBK etc. were downregulated in the LN(+) group, which reflects an upregulation in LN(−) group. The genes TEAD3, RGL2, ITFG3, BAT3, ATF6B, and RARA etc. were upregulated in the LN(+) group, which reflects a down-regulation in the LN(−) group.

### Degree of centrality analysis

If the relationship between log degree and log number of a gene is linear, the topology suggests there is a scale-free network, which refers to a network that appears in many natural phenomena in network analyses. In a scale-free network, the degree of each gene is uneven and is concentrated at a specific hub gene. Therefore, the number of hub gene degrees in a scale-free network follows a power-law distribution. Both networks in the LN(+) and LN(−) colorectal cancer groups showed a scale-free topology (Supplementary Fig. 2).

**Figure 1:**
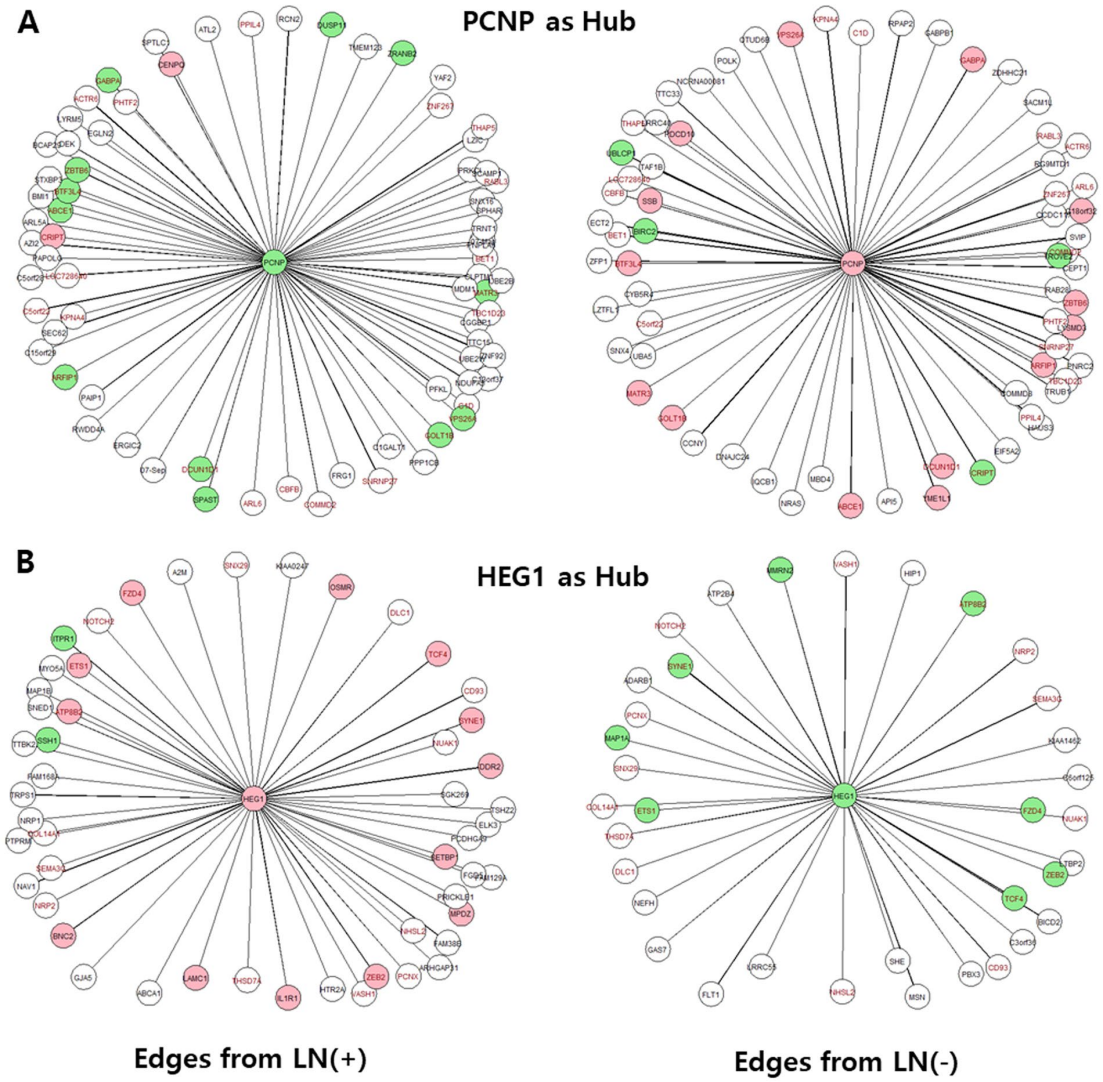
Representative hub with its edge genes calculated using the degree centrality analysis of the LN(+) and LN(−) groups. (**A**) PCNP and (**B**) HEG1 as hub genes. Green fill: downregulated genes in the DEG analysis, Red fill: upregulated genes in the DEG analysis, Red font: common genes in both groups, Edge width: coefficient power.

In the degree of centrality analysis, we calculated the number of hub genes from the pre-processed set of 17,009 genes, and as a result, a total of 16,579 hub genes with at least one edge (neighboring) gene were identified, with the degree of centrality of the edge genes sorted by degree (i.e. the number of edge genes) in each group (Supplementary Tables 2A and 3A). The mean degree per hub gene was ~7.5 with a range of 0–72 in the LN(+) group and ~7.7 with a range of 0–70 in LN(−) group, which was similar in both groups. Hub genes over 26 degrees (i.e. CV ≤ 20%) were selected, with 240 being identified in the LN(+) group and 353 in the LN(−) group, and analyzed further. As a result, 127 genes were identified as the hub (a common hub) in both groups (Supplementary Tables 2A, 3A, and 6), representing 52.9% (127/240) in the LN (+) group and 34.0% (127/353) in the LN(−) group. The mean degree for the 127 common hub genes was 33 in the LN(+) group and 35.9 in the LN(−) group. These 127 common hub genes shared 12.5 (38%) common edge genes with the LN(+) group and 12.5 (35.3%) common edge genes with the LN(−) group, with a range of 4–26, but did not share 20.5 (62%) common edge genes, with a range of 11–46, for the LN(+) group, and 23.3 (64.7%) common edge genes, with a range of 10–54, for the LN(−) group as different edges genes from each group. The representative network of hub and edge genes with high degrees in each group are shown and compared in Fig. 1 and Supplementary Fig. 3. This result indicates that there are gene network differences between the LN(−) and LN(+) groups.

A degree of centrality analysis was performed using the 240 selected hub genes in the LN(+) group with the LN(−) group, to investigate and compare how edge genes and degree are changed/altered with the same hub genes in each group (Supplementary Table 2B). A mean degree of 10.3 (32.7%) in the LN(+) group, and 10.3 (38.3%) in the LN(−) group, with a range of 0–26 per hub gene, was seen for the common edge genes in both groups, when the hub genes of the LN(+) group were applied to the LN(−) group. In the same manner, 353 hub genes in the LN(−) group were applied to the LN(+) group (Supplementary Table 3B). A mean degree of 9.3 (39.9%) in the LN(−) group and 9.3 (28.8%) in the LN(+) group, with a range of 0–26 per hub gene, was seen with common edge genes in both groups. This result implies that approximately 60–70% of the edge genes with the same hub gene are from each other’s groups confirming that the gene network differs between the LN (−) and (+) groups.

### Degree of centrality analysis of only the hub genes

**Figure 2:**
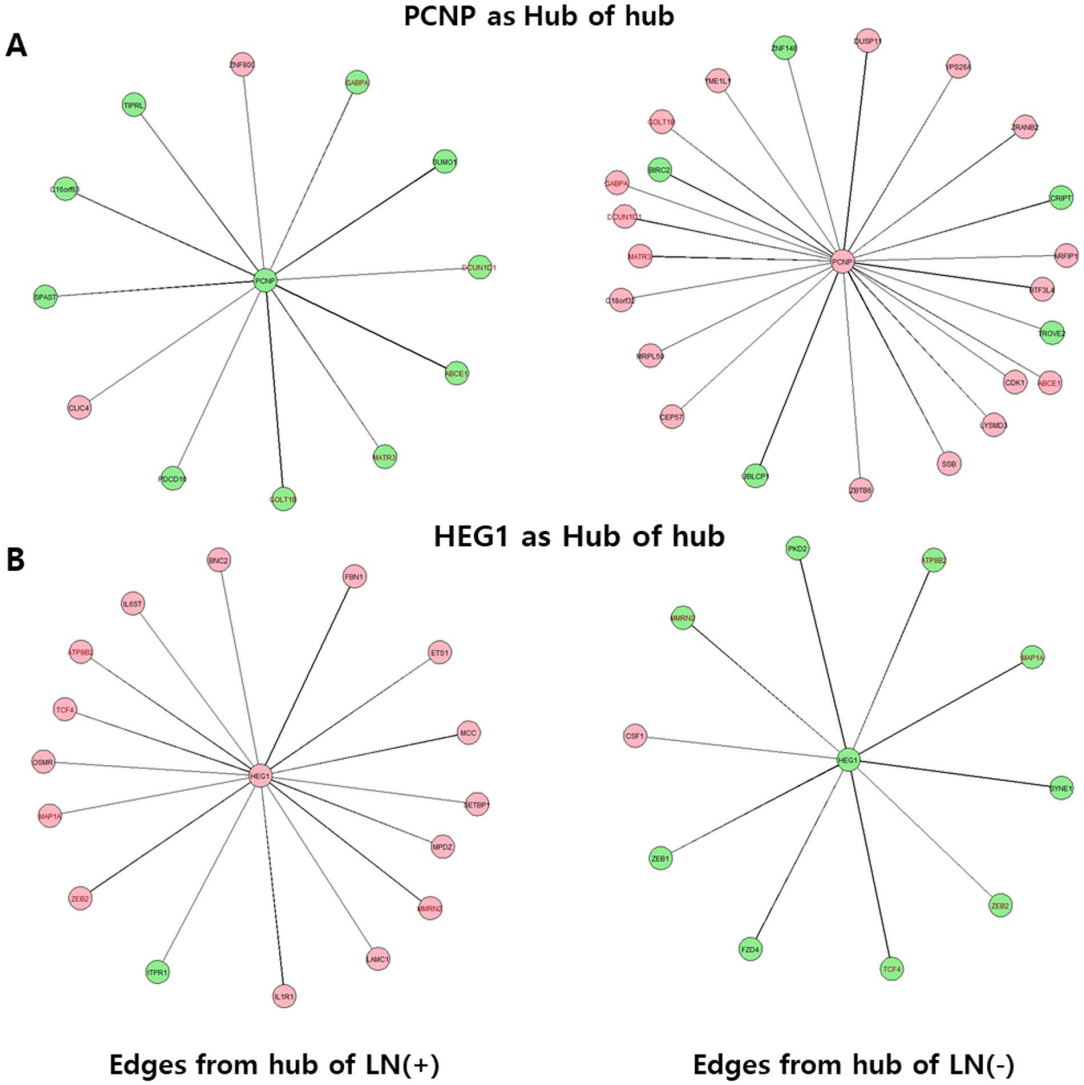
Representative the hub of hub gene with its edge genes calculated by the degree of centrality analysis from the LN(+) and LN(−) groups. (**A**) PCNP and (**B**) HEG1 as the hub of hub genes. Green fill: downregulated genes in the DEG analysis, Red fill: upregulated genes in the DEG analysis, Red font: common genes in both groups, Edge width: coefficient power.

A network analysis using only hub genes [240 from the LN(+) group and 353 from the LN(−) group], without counting their edge genes, was conducted to investigate the hub of hub genes (Fig. 2 and Supplementary Tables 4A and 5A) and understand the relationship between the hub genes. The mean degree of the hub of hub genes was 7.7 with a range of 2–16 in the LN(+) group and 8.3 with a range of 3–23 in the LN(−) group. The 127 common hub genes from both groups were also common in the hub of hub genes, with the mean degree of the common hub of hub genes being 8.4 in the LN(+) group and 9.3 in the LN(−) group. These common hub of hub genes shared a mean of 5.1 (62.7%) in the LN(+) group and 5.3 (57.8%) in the LN(−) group with a range of 1–12 as common edge genes and were not shared with a mean of 3.3 (37.3%) and a range of 0-–0 in the LN(+) group and 4.0 (42.2%) with a range of 0–11 in the LN(−) group representing different edge genes from each group. This result indicates that hub genes with a high degree are implicated as important genes in the gene network and are still shared by both groups, even if the edge genes are changed. The representative network of the hub of hub genes and the edge genes with a high degree from each group are shown in Fig. 2 and Supplementary Fig. 4.

In addition, a degree of centrality analysis was performed using the 240 selected hub of hub genes of the LN(+) group in the LN(−) group, to investigate and compare how edge genes and their degree changes with the same hub genes in each other’s group (Supplementary Table 4B). Mean degrees of 4.5 (61.3%) were found in the LN(+) group and 4.5 (61.7%) in the LN(−) group, with a range of 0–12 per hub of hub gene with common edge genes in both group, when the hub genes in the LN(+) group were applied to the LN(−) group. An identical analysis was conducted using the 353 hub genes in the LN(−) group (Supplementary Table 5B). A mean degree of 4.2 (52.3%) was found in the LN(−) group, and a mean degree of 4.2 (52.3%) was found in the LN(+) group, with a range of 0–12 per hub of hub gene, with common edge genes in both group, when hub genes in the LN(−) group were applied to the LN(+) group. This result indicates that approximately 38–48% of the edge genes for the same hub genes are different in each group confirming that high degree hub genes, which may play an important role in the gene network, still exist as a hub gene network in both groups.

**Figure 3:**
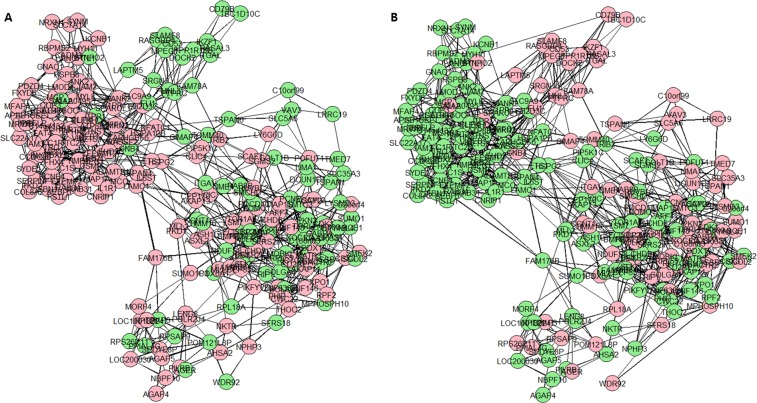
Degree of centrality analysis of the top 240 hub genes in the LN(+) group. (**A)** 240 Hub genes in the LN(+) group. (**B**) Hub genes (240) in the LN(−) group. The location of each gene in (**A**,**B**) is identical. Green fill: downregulated genes in the DEG analysis, Red fill: upregulated genes in the DEG analysis, Edge width: coefficient power.

Furthermore, the network between hub genes was determined and the hub genes from one group were applied to the other group. Data showed that the network of hub genes from the LN(+) group changed when the same hub genes were applied to the LN(−) group (Fig. 3). The opposite analysis showed a consistent result, indicating once again that the network between the LN(−) and LN(+) groups had changed (Supplementary Fig. 5). In addition, the networks of common hubs from both groups were compared and found to be very different, confirming that the relationship between hub genes differed between the LN(−) group and the LN(+) group (Supplementary Fig. 6).

### Comparison of DEG sets and hub genes from each group

**Figure 4:**
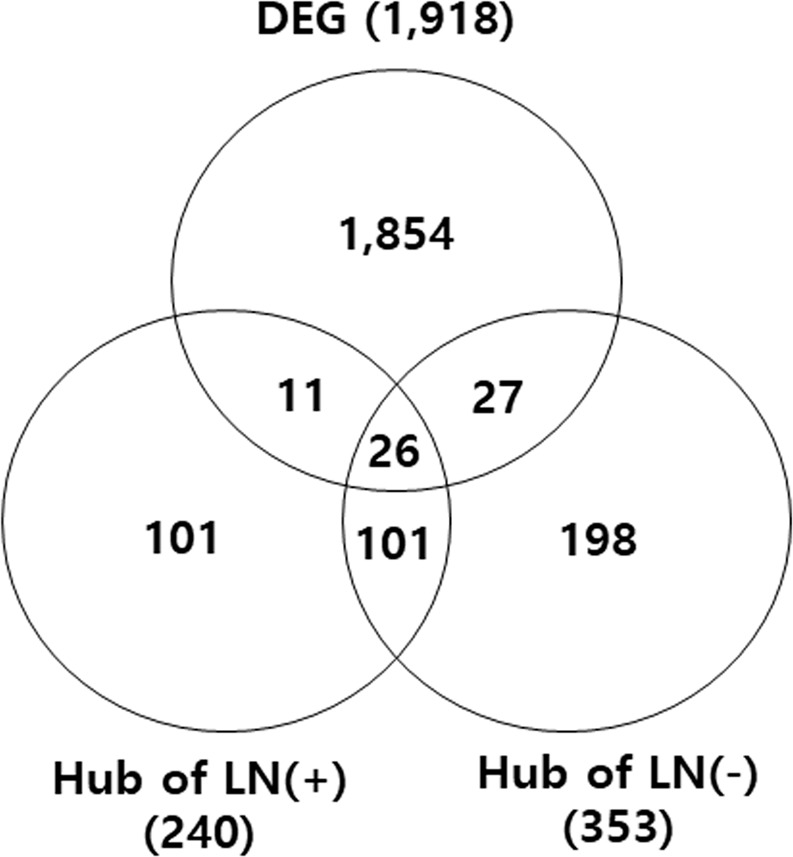
Venn diagram of genes shared across the 1918 DEG (p < 0.005) sets and hub genes (CV ≤ 20%) from each group. The number indicates the number of genes, which listed in Table 2.

Hub genes from the LN(+) and LN(−) groups were compared with the DEG set (1918 genes) to select hub genes which were highly differentially expressed between the two groups, and which may be serve as a marker to distinguish LN(−) for LN(+), and which could potentially be used as a prognostic markers (Supplementary Table 7). The analysis revealed that as the hub genes in both the LN(+) and LN(−) group, five genes were downregulated and 21 genes were upregulated in LN(+) group compare to LN(−) groups. As hub genes in the LN(+) group, three genes were downregulated and eight genes were upregulated and in the LN(−) group, thirteen genes were downregulated and 14 genes were upregulated in LN(+) group compare to LN(−) group (Fig. 4 and Table 2). Furthermore, the expression level differences between hub genes in the LN(+) and LN(−) groups were examined, and it was shown that 155 hub genes showed significantly (p ≤ 0.05) different expression levels between the LN(+) group and the LN(−) group, even if these genes were not included in 1918 DEG set (Supplementary Table 7).

**Table 2:** Selected hub genes by comparison DEG sets and hub genes from each group.

Degree Centrality			LN(+)	LN(−)	Relative gene expression level [LN(+)/LN(−)]	p-value
			179	216		
DEG&LN(+) &LN(−) [26]	SLC22A17	degree	40	28	up	0.0000
		median of expression (log2)	6.456	5.991		
	APBB1	degree	37	43	up	0.0001
		median of expression (log2)	7.009	6.599		
	SLC7A14	degree	28	31	up	0.0002
		median of expression (log2)	0.952	0		
	JAM3	degree	26	36	up	0.0002
		median of expression (log2)	7.998	7.722		
	PRELP	degree	30	27	up	0.0004
		median of expression (log2)	7.495	6.542		
	LYSMD3	degree	28	29	down	0.0004
		median of expression (log2)	8.554	8.757		
	RBPMS2	degree	29	34	up	0.0008
		median of expression (log2)	4.736	4.345		
	LMOD1	degree	32	30	up	0.0008
		median of expression (log2)	8.205	7.599		
	GEFT	degree	35	26	up	0.0010
		median of expression (log2)	7.006	6.459		
	SALL2	degree	26	27	up	0.0010
		median of expression (log2)	4.872	4.601		
	TNS1	degree	32	46	up	0.0010
		median of expression (log2)	10.254	9.675		
	EFEMP2	degree	31	40	up	0.0014
		median of expression (log2)	9.071	8.798		
	SYDE1	degree	27	34	up	0.0016
		median of expression (log2)	7.844	7.579		
	CLIP3	degree	48	37	up	0.0016
		median of expression (log2)	7.515	7.128		
	MRVI1	degree	30	35	up	0.0016
		median of expression (log2)	8.567	8.244		
	PKN2	degree	26	36	down	0.0018
		median of expression (log2)	9.935	10.147		
	AHSA2	degree	34	51	up	0.0022
		median of expression (log2)	8.660	8.378		
	AKAP11	degree	28	28	up	0.0026
		median of expression (log2)	10.494	10.250		
	TIMP2	degree	36	35	up	0.0026
		median of expression (log2)	12.056	11.636		
	CDK1	degree	33	32	down	0.0029
		median of expression (log2)	10.289	10.405		
	ABCE1	degree	28	34	down	0.0030
		median of expression (log2)	10.849	10.973		
	PKD1	degree	27	27	up	0.0031
		median of expression (log2)	10.563	10.364		
	SGMS2	degree	26	32	down	0.0033
		median of expression (log2)	9.014	9.180		
	MGP	degree	41	39	up	0.0034
		median of expression (log2)	9.336	8.834		
	HSPB8	degree	30	28	up	0.0037
		median of expression (log2)	6.676	6.234		
	BOC	degree	41	53	up	0.0039
		median of expression (log2)	6.997	6.573		
DEG&LN(+) ^11^	TMTC3	degree	28	24	down	0.0004
		median of expression (log2)	8.432	8.568		
	FXYD6	degree	28	20	up	0.0004
		median of expression (log2)	8.095	7.513		
	PDZD4	degree	33	10	up	0.0007
		median of expression (log2)	4.063	3.538		
	SLC35A3	degree	26	19	down	0.0009
		median of expression (log2)	9.293	9.592		
	TMED7	degree	28	25	down	0.0014
		median of expression (log2)	11.003	11.198		
	SCAF1	degree	33	16	up	0.0017
		median of expression (log2)	10.830	10.641		
	TUB	degree	27	15	up	0.0019
		median of expression (log2)	4.819	4.296		
	MYH11	degree	29	21	up	0.0023
		median of expression (log2)	10.712	10.094		
	C14orf132	degree	31	20	up	0.0026
		median of expression (log2)	6.466	5.961		
	SPARCL1	degree	28	21	up	0.0034
		median of expression (log2)	10.075	9.712		
	TRO	degree	31	15	up	0.0035
		median of expression (log2)	5.470	5.182		
DEG&LN(−) [27]	C12orf48	degree	17	26	down	0.0000
		median of expression (log2)	8.204	8.499		
	C14orf129	degree	15	30	down	0.0000
		median of expression (log2)	10.160	10.653		
	C18orf32	degree	20	33	down	0.0002
		median of expression (log2)	9.154	9.436		
	PDLIM7	degree	16	34	up	0.0004
		median of expression (log2)	9.937	9.549		
	COPS4	degree	19	28	down	0.0004
		median of expression (log2)	9.258	9.414		
	ADAMTSL3	degree	19	26	up	0.0005
		median of expression (log2)	4.943	4.269		
	FHL1	degree	23	30	up	0.0005
		median of expression (log2)	8.568	8.263		
	GPRASP1	degree	19	40	up	0.0006
		median of expression (log2)	5.955	5.530		
	HMCN1	degree	20	29	up	0.0007
		median of expression (log2)	6.655	6.041		
	GBP4	degree	14	26	down	0.0010
		median of expression (log2)	8.569	9.076		
	JAK2	degree	18	26	down	0.0011
		median of expression (log2)	7.856	8.159		
	MXRA8	degree	23	28	up	0.0012
		median of expression (log2)	9.792	9.426		
	SETD1A	degree	14	28	up	0.0012
		median of expression (log2)	9.872	9.755		
	RAB27B	degree	14	26	down	0.0013
		median of expression (log2)	4.289	5.011		
	TNRC6A	degree	10	26	up	0.0014
		median of expression (log2)	9.662	9.499		
	NUMA1	degree	21	26	up	0.0014
		median of expression (log2)	12.548	12.352		
	MRPL50	degree	11	28	down	0.0022
		median of expression (log2)	9.049	9.150		
	ZNF24	degree	21	28	down	0.0026
		median of expression (log2)	9.947	10.147		
	LONRF2	degree	22	27	up	0.0034
		median of expression (log2)	2.224	1.616		
	ZNF767	degree	16	26	up	0.0036
		median of expression (log2)	7.424	7.248		
	ARFIP1	degree	22	40	down	0.0037
		median of expression (log2)	10.082	10.215		
	USP33	degree	10	26	down	0.0037
		median of expression (log2)	9.917	10.092		
	C5orf44	degree	19	34	down	0.0042
		median of expression (log2)	8.568	8.676		
	ZNF720	degree	16	26	up	0.0045
		median of expression (log2)	7.938	7.768		
	UBA3	degree	13	39	down	0.0046
		median of expression (log2)	9.826	9.953		
	LDB2	degree	19	26	up	0.0048
		median of expression (log2)	6.801	6.555		
	CDK10	degree	14	33	up	0.0049
		median of expression (log2)	10.293	10.166		

### Survival analysis with selected hub genes

**Figure 5:**
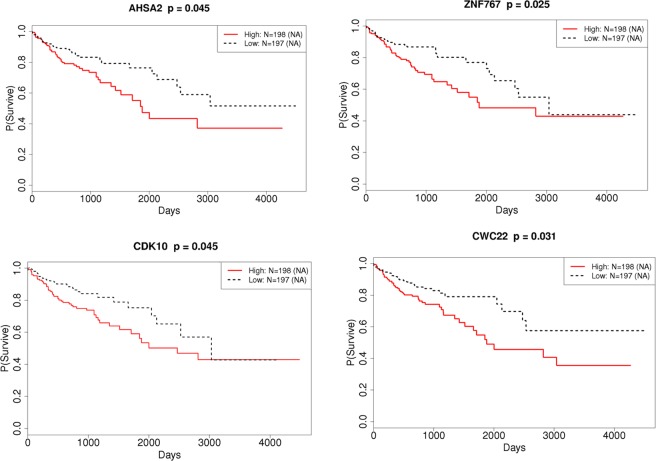
Representative Kaplan-Meier survival curves of selected hub genes. AHSA2, ZNF767, CDK10, and CWC22.

To understand the potential use of these selected genes for prognosis we compared the DEG set, and the hub genes as well as the hub of hub genes with high degree, a survival analysis using a Kaplan-Meier estimation was performed (Fig. 5, Supplementary Fig. 6, and Supplementary Table 8). When the survival rate was compared with the expression levels of hub genes, the results were consistent with our expectation. Hub genes that were upregulated by the DEG analysis in the LN(+) group, such as AHSA2, ZNF767, and CDK10 showed a significantly (p ≤ 0.05) reduced survival rate in the up-regulated group compared to the down-regulated group. However, the hub genes selected as downregulated by the DEG analysis in the LN(+) group showed a tendency, but not significantly, toward a reduced survival rate in the down-regulated group compared with the up-regulated group. CWC22, a hub gene which at the same time functions as the hub of hub genes with a high degree, and which was not a significant DEG, also showed significant survival rate differences. This result indicates the possibility of using these selected hub genes identified from a network analysis as prognostic markers.

### Protein-protein interaction network, GO and KEGG pathway enrichment analysis with selected hub genes

**Figure 6:**
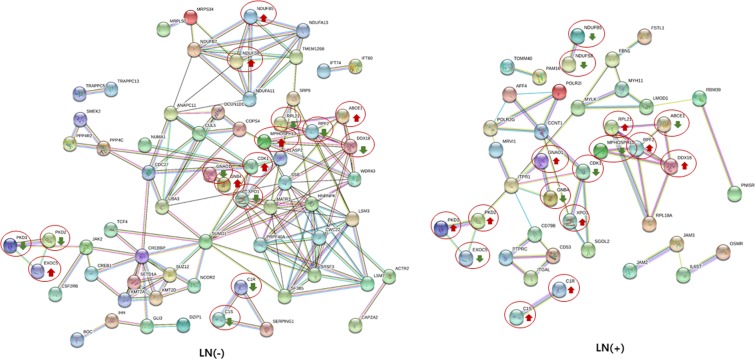
Protein-protein interaction network among the hub genes from LN(−) and LN(+) with more than a 0.95 confidence score as analyzed by STRING. Balls represent proteins, and lines represent interactions between proteins. A red circle around a ball indicates genes shared among both groups. Red arrow indicates upregulation. Green arrow indicates downregulation.

The results of STRING analysis showed a protein-protein interaction network of 41 hub genes (17.08%) in the LN (+) group and 66 hub genes (18.70%) in the LN(−) group, with >0.95 confidence score. Of these hub genes, 16 were shared among both groups, while 50 hub genes from LN(−) replaced 25 different hub genes from LN(+), resulting in protein-protein network differences between the LN(−) and LN(+) groups (Fig. 6 and Supplementary Table 9). However, interactions between the shared hub genes did not differ between groups, which retained from LN(+) to LN(−) group.

**Figure 7:**
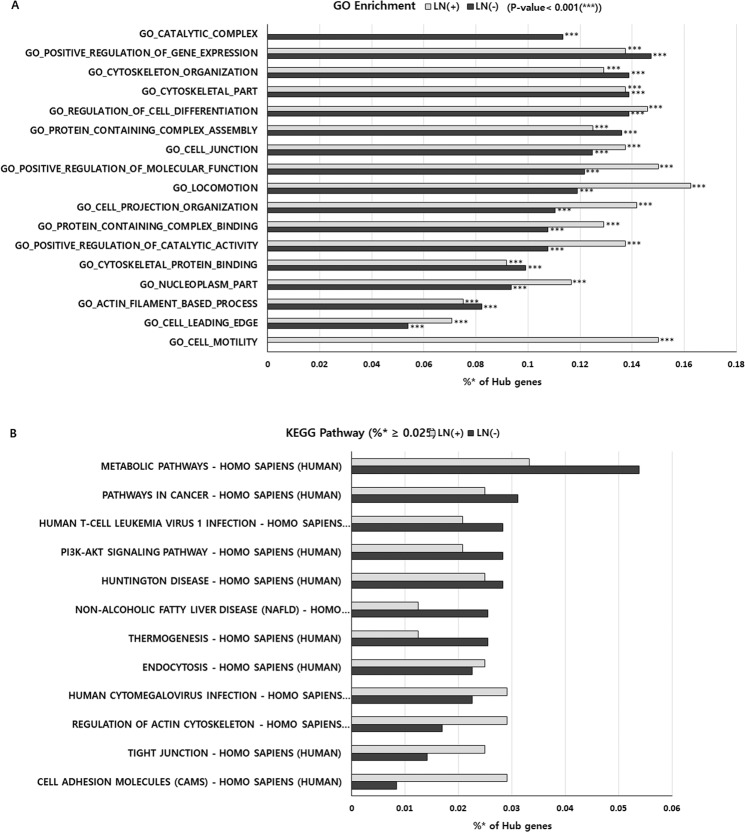
A. Top 10 enriched GO terms B. KEGG pathway with more than 0.025% of the hub genes involved [searched using 353 hub genes from LN(−) and 240 hub genes from LN(+)]. *Indicates proportion of the number of genes: [Number of hub genes involved in this pathway/number of total hub genes from LN(+) or LN(−)] × 100.

To examine the characteristics of the hub genes from each group, the functional classifications of the hub genes were searched using the GO tool. The top 100 most significantly enriched GO terms for biological process from each group were determined (Supplementary Table 10). Of these 100 enriched GO terms, 54 were common among both groups and 46 differed between groups. The top ten most highly enriched GO terms from each group were selected and compared (Fig. 7A). Results showed that hub genes from the LN(+) group were enriched for cell motility and cell locomotion relative to that in the LN(−) group.

KEGG pathway analysis was also performed to further understand the biological functions of the genes. A total of 150 pathways from the LN(+) and 213 pathways from the LN(−) groups were enriched (Supplementary Table 11). Of these pathways, 142 pathways were common between both groups, eight pathways (5.3%) were enriched only in the LN(+) group, and 71 pathways (33.3%) were enriched only in the LN(−) group. Pathways where more than 0.025% of hub genes were involved were compared between the LN(−) and LN(+) groups (Fig. 7B); this analysis highlighted the differences at the level of the metabolic and cell adhesion molecule pathways.

## Discussion

The genetic basis of the development of colon cancer is well understood, however prognostic factors related to the LN involvement are still under investigation. Here, we have attempted to understand the pathophysiology of colon cancer and how the gene changes from LN(−) to LN(+) using a network analysis and by comparing DEGs in LN(+) and LN(−) groups of colon cancer patients using the TCGA data set. Our data showed that the gene network differs from LN(−) to LN(+), since 62–64.7% of edge genes for the same hub genes from both group differed between the two groups. However, main hub genes, such as PNCP, HEG1, SECISBP2L, and CWC22 etc. were still present, even though the edge genes changed from the LN(−) to the LN(+) group. Furthermore, the hub of hub genes with a high degree, such as HEG1, SECISBP2L, TCF4, CLIP3, MSRB3, PCNP, VIM, and ATP8B2, were hub genes with a high degree in the LN(+) group, and the hub of hub genes with a high degree, such as PCNP, XPO1, TNS1, PIKFYVE, VPS26A, CWC22, CCDC80, MATR3, ZEB1, C1S, AFF4, ZEB2, LY6G6D, and SSB, were hub genes with a high degree in the LN(−) group. These results imply that the important hub genes are not altered even if their edge genes are changed from the LN(−) to LN(+) groups. Of these selected hub genes, or hub of hub genes, *PCNP*, which selected as both a hub gene, as well as a hub of hub gene with a high degree in both groups. It is known that *PCNP* is related to control of the cell cycle and may be involved in tumorigenesis^15,16^, however, to date there have been no reports suggesting a role for *PCNP* in colon cancer. Another interesting hub gene observed was *HEG1*, a heart development protein with EGF-like domains 1, which is known to be associated with the stabilization of cell–cell junctions^17^ and has been suggested as a tumor marker and a therapeutic target in malignant mesothelioma^18^.

To investigate possible markers to distinguish LN(−) to LN(+), hub genes from the LN(+) and LN(−) groups were compared with the DEG set to select hub genes that were highly differentially expressed between the two groups. Hub genes which were highly differentially expressed, such as APBB1, AHSA2, ZNF767, and JAK2 etc., were included within the 1918 DEGs set. A survival analysis using selected hub genes, such as AHSA2, ZNF767, SECISBP2L, CDK10 and CWC22, showed that their expression levels were significantly associated with survival rate, indicating the possibility that they could be useful as prognostic markers; these genes could not have been identified by a DEG analysis alone. AHSA2, as a hub gene, was found to be upregulated in the LN(+) group compared to the LN(−) group and was significantly associated with survival. AHSA2(AHA1) is an activator of the heat shock 90 kDa protein ATPase homolog 2, and belongs to the AHA family, which encodes proteins that can activate the ATPase activity of Hsp90 as co-chaperones^19^. The basal level of expression of AHA1 is different across a panel of different human cancer cell lines, however HCT116 cells, which is known to be a highly aggressive colon cell line, showed increased expression levels of AHA1 compared to HT29 cells, which is a less aggressive colon cancer cell line^20^. Thus, modulation of AHA1 has been suggested as a potential therapeutic strategy to increase the sensitivity to HSP90 inhibitors, since treatment with 17-AAG results in the sustained up-regulation of AHA1, and in addition the silencing of AHA1 expression increases cellular sensitivity to an HSP90 inhibitor^21^. Function of ZNF767, which is also edge gene of AHSA2 in our data, and SECISBP2L has not been studied yet. CDK10, cyclin dependent kinase 10, has been reported high expression in colon cancer and inactivation of its kinase domain showed prevention of tumor growth lately^22^. CWC22, the other upregulated hub genes in the LN(+) group, is a CWC22 spliceosome associated protein and has been suggested to be an unfavorable prognostic marker in renal and liver cancer (https://www.proteinatlas.org/ENSG00000163510-CWC22/pathology), although its function still needs to be investigated. However, hub genes, such as PCNP and HEG1, were not identified as DEGs between the LN(+) vs, LN(−) groups, even if their edge genes were changed. It is possible that there are other mechanisms, not expression differences, which need to be further explored.

In addition, the protein-protein interaction network, GO enrichment, and KEGG pathway were searched using the selected hub genes from each group. A STRING analysis was performed to further explore the physical and functional protein interaction networks among the hub genes from each group, and the results showed changes in the protein-protein interactions among the hub genes, as 50 hub genes from the LN(−) group were replaced by 25 different hub genes in the LN(+) group. Four hub genes (MYH11, MRVI1, LMOD1, and JAM3) from the LN(+) group, seven hub genes (UBA3, SETD1A, NUMA1, MRPL50, JAK2, COPS4, and BOC) from the LN (−) group, and three hub genes (PKD1, CDK1, and ABCE1) from both groups were included in the 1918 DEG (p < 0.005) set, indicating differential expression between the LN(−) and LN(+) groups (Table 2). However, survival analysis using a Kaplan-Meier estimation of these genes was not significant between LN(+) and LN(−) (Supplementary Fig. 7). In the GO enrichment analysis, cell motility enrichment was only shown in the LN(+) group, and cell locomotion enrichment was higher in the LN(+) group than that in the LN(−) group. The results of the KEGG pathway analysis showed differences at the level of the metabolic and cell adhesion molecule pathways. The cell adhesion molecule pathway is known to be associated with cell motility. Taken together, the GO and KEGG results implied that the hub genes from the LN(+) group are more related with cell movement and metastatic ability.

In conclusion, using a gene expression network analysis, we have identified hub genes, such as AHSA2, CDK10, and CWC22, as being possible prognostic markers, that were not previously known to be associated with colon cancer. Additionally, the regulatory relationships among the hub genes with respect to biological processes, and the LN involvement in colon cancer were different.Since we only used gene expression data for network construction, further research is needed to confirm the role of these genes in colon cancer. The results of this network analysis may help narrow down the search for novel candidate genes for the treatment of colon cancer, in addition to improving our understanding of the biological events underlying LN involvement in colon cancer.

## Supplementary information

Supplementary Data.

Supplementary Figures.

R implementation codes.

## References

[1] Jemal A, Center MM, DeSantis C, Ward EM (2010). Global patterns of cancer incidence and mortality rates and trends. Cancer Epidemiol. Biomarkers Prev..

[2] Vogelstein B (1988). Genetic alterations during colorectal-tumor development. N. Engl. J. Med..

[3] Ponz de Leon M, Percesepe A (2000). Pathogenesis of colorectal cancer. Dig. Liver Dis..

[4] Grady WM, Markowitz SD (2015). The molecular pathogenesis of colorectal cancer and its potential application to colorectal cancer screening. Dig. Dis. Sci..

[5] Cancer Genome Atlas Network (2012). Comprehensive molecular characterization of human colon and rectal cancer. Nature.

[6] Vlad C, Kubelac P, Vlad D, Irimie A, Achimas Cadariu P (2015). Evaluation of clinical, morphopathological and therapeutic prognostic factors in rectal cancer. Experience of a tertiary oncology center. J. BUON.

[7] Edler D, Ohrling K, Hallstrom M, Karlberg M, Ragnhammar P (2007). The number of analyzed lymph nodes - a prognostic factor in colorectal cancer. Acta Oncol..

[8] Hudson NJ, Dalrymple BP, Reverter A (2012). Beyond differential expression: the quest for causal mutations and effector molecules. BMC Genomics.

[9] Wu C, Zhu J, Zhang X (2013). Network-based differential gene expression analysis suggests cell cycle related genes regulated by E2F1 underlie the molecular difference between smoker and non-smoker lung adenocarcinoma. BMC Bioinformatics.

[10] Koschutzki D, Schreiber F (2008). Centrality analysis methods for biological networks and their application to gene regulatory networks. Gene Regul. Syst. Bio..

[11] Cancer Genome Atlas Research Network (2013). The Cancer Genome Atlas Pan-Cancer analysis project. Nat. Genet..

[12] Meinshausen, N. & Bühlmann, P. J. T. A. O. S. High-dimensional graphs and variable selection with the lasso. **34**, 1436–1462 (2006).

[13] Kittelson JM (2011). A Review of: “Fundamentals of Biostatistics, 7th ed., by B. Rosner”. J. Biopharm. Stat..

[14] Goel MK, Khanna P, Kishore J (2010). Understanding survival analysis: Kaplan-Meier estimate. Int. J. Ayurveda Res..

[15] Mori T, Li Y, Hata H, Kochi H (2004). NIRF is a ubiquitin ligase that is capable of ubiquitinating PCNP, a PEST-containing nuclear protein. FEBS Lett..

[16] Mori T, Li Y, Hata H, Ono K, Kochi H (2002). NIRF, a novel RING finger protein, is involved in cell-cycle regulation. Biochem. Biophys. Res. Commun..

[17] de Kreuk BJ (2016). Heart of glass anchors Rasip1 at endothelial cell-cell junctions to support vascular integrity. eLife.

[18] Tsuji S (2017). HEG1 is a novel mucin-like membrane protein that serves as a diagnostic and therapeutic target for malignant mesothelioma. Sci. Rep..

[19] Panaretou B (2002). Activation of the ATPase activity of hsp90 by the stress-regulated cochaperone aha1. Mol. Cell.

[20] Yeung TM, Gandhi SC, Wilding JL, Muschel R, Bodmer WF (2010). Cancer stem cells from colorectal cancer-derived cell lines. Proc. Natl. Acad. Sci. USA.

[21] Holmes JL, Sharp SY, Hobbs S, Workman P (2008). Silencing of HSP90 cochaperone AHA1 expression decreases client protein activation and increases cellular sensitivity to the HSP90 inhibitor 17-allylamino-17-demethoxygeldanamycin. Cancer Res..

[22] Weiswald LB (2017). Inactivation of the Kinase Domain of CDK10 Prevents Tumor Growth in a Preclinical Model of Colorectal Cancer, and Is Accompanied by Downregulation of Bcl-2. Mol. Cancer Ther..

