# Supplementary material for: Gene expression network analysis of lymph node involvement in colon cancer identifies AHSA2, CDK10, and CWC22 as possible prognostic markers
Source: Sci Rep. 2020 Apr 28;10:7170. doi: 10.1038/s41598-020-63806-x (PMC7189385; doi:10.1038/s41598-020-63806-x)
Supplement: Supplementary file 2 — Supplementary Figures. [file 41598_2020_63806_MOESM2_ESM.pdf]

Gene expression network analysis of lymph node involvement in colon cancer identifies AHSA2, CDK10, and CWC22 as possible prognostic markers

Sung Won Han[1,+], Ji Young Ahn[1,+], Soobin Lee[1], YoungSeon Noh[1], Hee Chan Jung[2], Min Hyung Lee[1], Hae Jun Park[1], Hoon Jai Chun[3], Seong Ji Choi[3], Eun Sun Kim[3,*] and Ji Yun Lee[4,*]

Supplementary Information

List of supplementary Table 1- 11 (supplementary data: excel)

Supplementary Figures 1-7

List of supplementary Tables (Excel files)

Supplementary Table 1. 1918 genes selected ($p < 0.005$) from DEG analysis of the LN(+) and LN(-) group with colorectal cancer collected from TCGA

Supplementary Table 2A. Degree of centrality analysis of the LN(+) hub genes with their edges. Hub genes (CV≤20%) with their edge genes used for further analysis. The green fill indicates the common hub and edge genes (CV≤20%) in the LN(-) group (Supplementary Table 3A). The bolded genes indicate the different edge genes when the 240 hub genes (CV≤ 20%) were applied to the LN(-) group (Supplementary Table 2B)

Supplementary Table 2B. Degree of centrality analysis of the 240 hub genes for the LN(+) group (CV≤20%) in the LN(-) group. Bolded genes indicate the different edge genes when the 240 hub genes (CV≤20%) were applied to the LN(+) group (Supplementary Table 2A)

Supplementary Table 3A. Degree of centrality analysis of the LN(-) hub genes with their edges. Hub genes (CV≤20%) with their edge genes were used for further analysis. The green fill indicates common hub and edge genes (CV≤20%) in the LN(+) group (Supplementary Table 2A). Bolded genes indicate different edge genes when the 353 hub genes (CV≤20%) were applied to the LN(+) group (Supplementary Table 3B)

Supplementary Table 3B. Degree of centrality analysis of the 353 hub gene for the LN(-) group (CV≤20%) in the LN(+) group. Bolded gene indicate different edge genes when the 353 hub genes (CV≤20%) were applied to the LN(-) group (Supplementary Table 3A)

List of supplementary Tables (Excel files): continued

Supplementary Table 4A. Degree of centrality analysis of the hub of hub genes with their edge genes, using only 240 hub genes and discounting their edge genes in the LN(+) group. Bolded gene indicate the different edge genes when the 240 hub genes were applied to the LN(-) group (Supplementary Table 4B). The green fill indicates common hub and edge genes (CV≤20% for the hub genes) in the LN(-) group (Supplementary Table 5A)

Supplementary Table 4B. Degree of centrality analysis of the hub of hub genes with their edge genes, using only the 240 hub genes from the LN(+) group in the LN(-) group. Bolded gene indicate the different edge genes when the 240 hub genes were applied to the LN(+) group (Supplementary Table 4A)

Supplementary Table 5A. Degree of centrality analysis of the hub of hub genes with their edge genes, using only the 353 hub genes and discounting their edge genes in the LN(-) group. Bolded gene indicate the different edge genes when the 353 hub genes were applied to the LN(+) group (Supplementary Table 5B). The green fill indicates common hub and edge genes (CV≤20% for the hub gene) in the LN(+) group (Supplementary Table 4A)

Supplementary Table 5B. Degree of centrality analysis of the hub of hub genes with their edge genes using only the 353 hub genes from the LN(-) group in the LN(+) group. Bolded gene indicate the different edge genes when the 353 hub genes were applied to the LN(-) group (Supplementary Table 5A)

List of supplementary Tables (Excel files): continued

Supplementary Table 6. Summary of common and different hub (= the hub of hub) genes in LN(+) and LN(-)

Supplementary Table 7. DEG of hub genes in LN(+) and LN(-). The yellow fill indicates a p-value < 0.05 for the hub genes

Supplementary Table 8. Survival analysis using the selected hub genes

Supplementary Table 9. List of hub genes investigated [353 hub genes from LN(-) and 240 hub genes from LN(+)] by STRING analysis (Confidence score ≥ 0.95)

Supplementary Table 10. Enriched GO terms obtained upon searching 353 hub genes from the LN(-) and 240 hub genes from the LN(+) groups

Supplementary Table 11. Enriched pathways based on KEGG pathway analysis of the 353 hub genes from LN(-) and 241 hub genes from LN(+)

Supplementary Figure 1. Heatmap of the top 100 differentially expressed genes (DEGs) classified into each group.

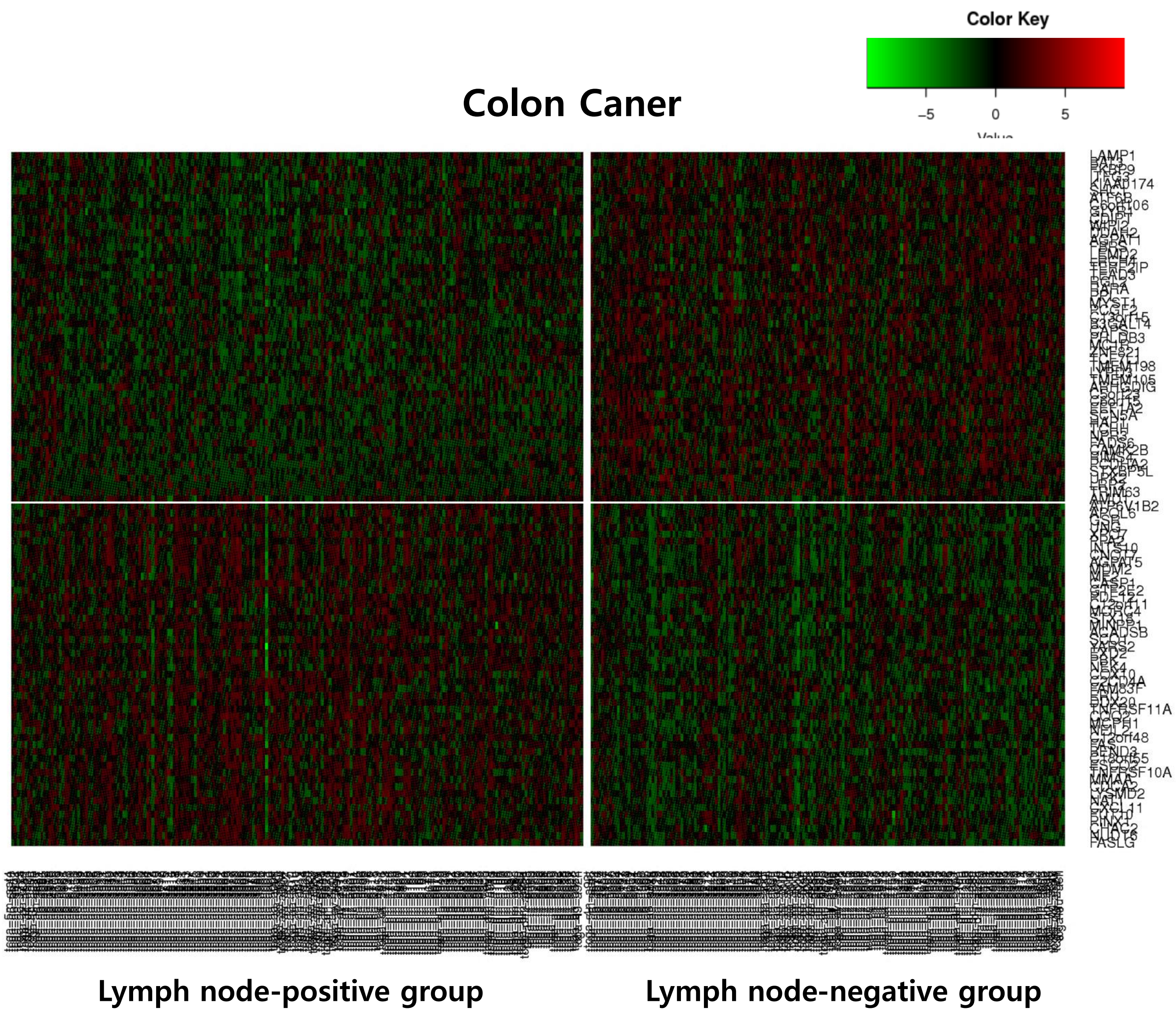

Supplementary Figure 2. Linear relationship between log degree and the log number of nodes A. LN(+) (R^2 = 0.79) B. LN(-) (R^2 = 0.80)

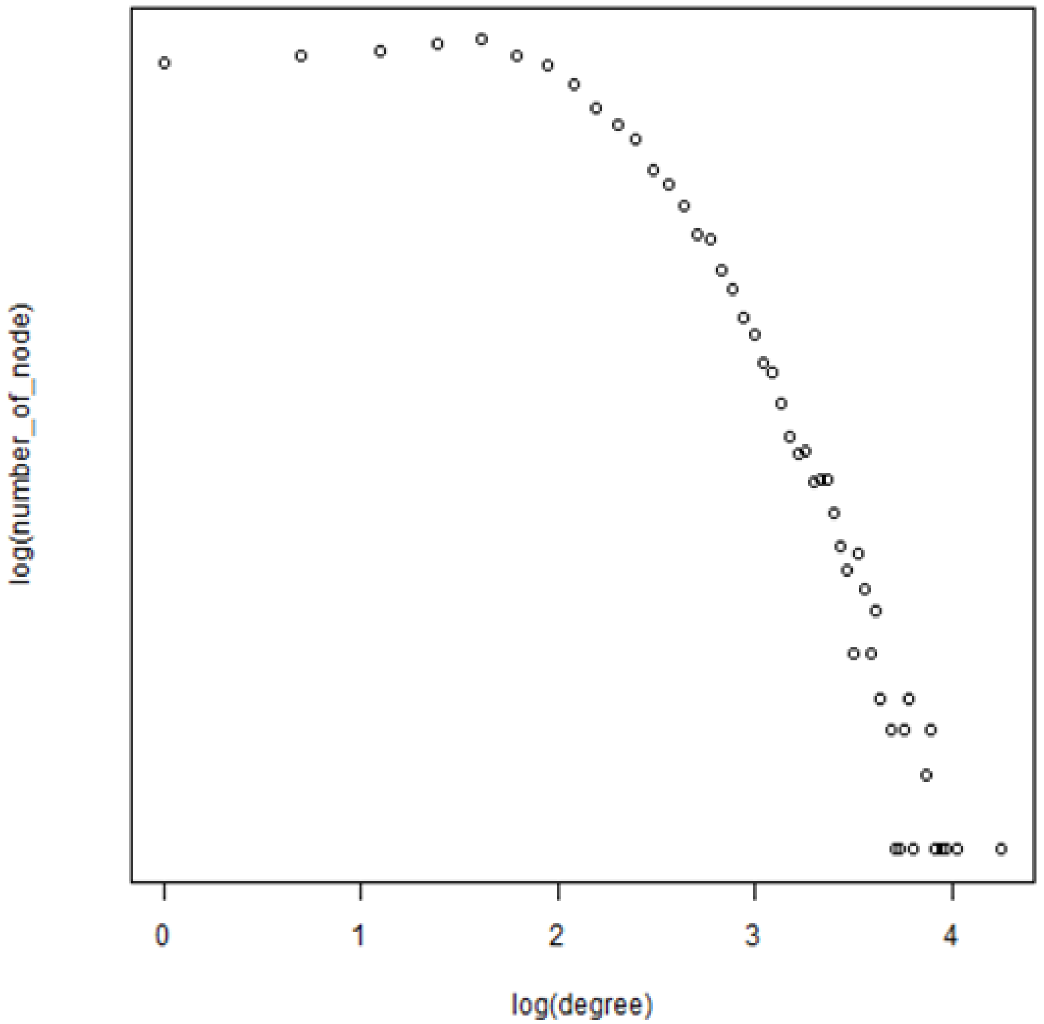

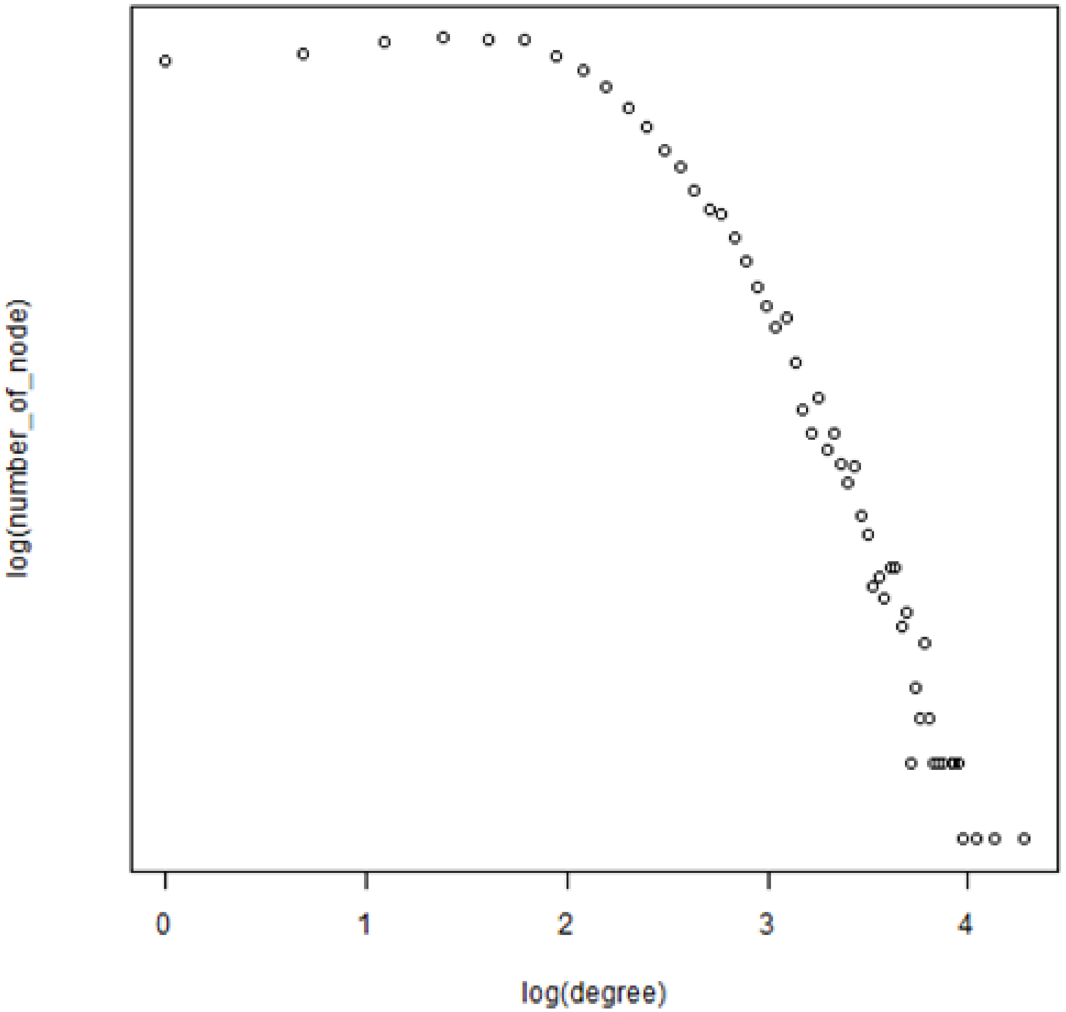

Supplementary Figure 3. Representative 26 of hub with its edge genes calculated by the degree of centrality analysis from the LN(+) and LN(-) groups (addition to Figure 1). Green fill: downregulated genes in the DEG analysis, Red fill: upregulated genes in the DEG analysis, Red font: common genes in both groups, Edge width: coefficient power

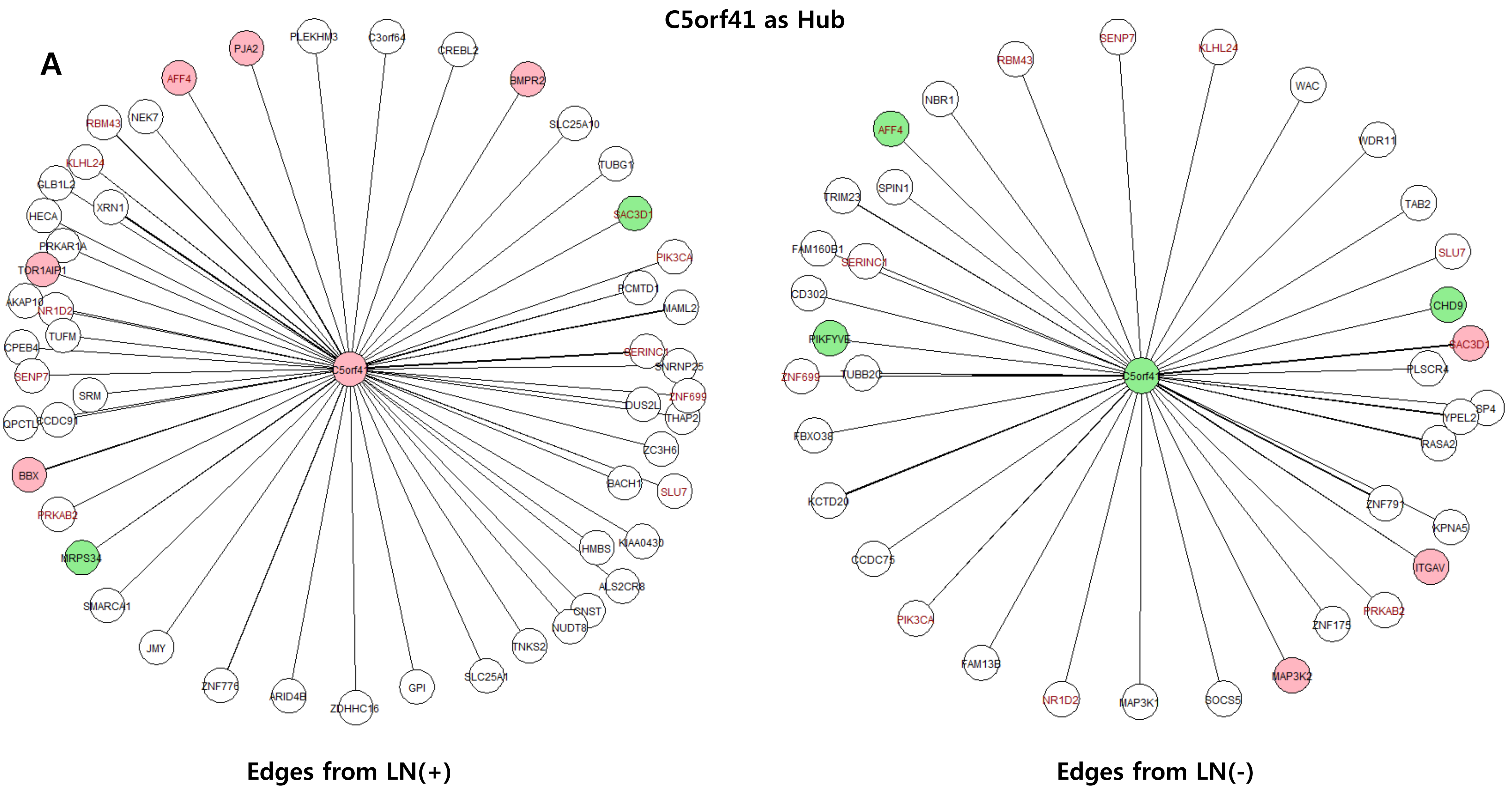

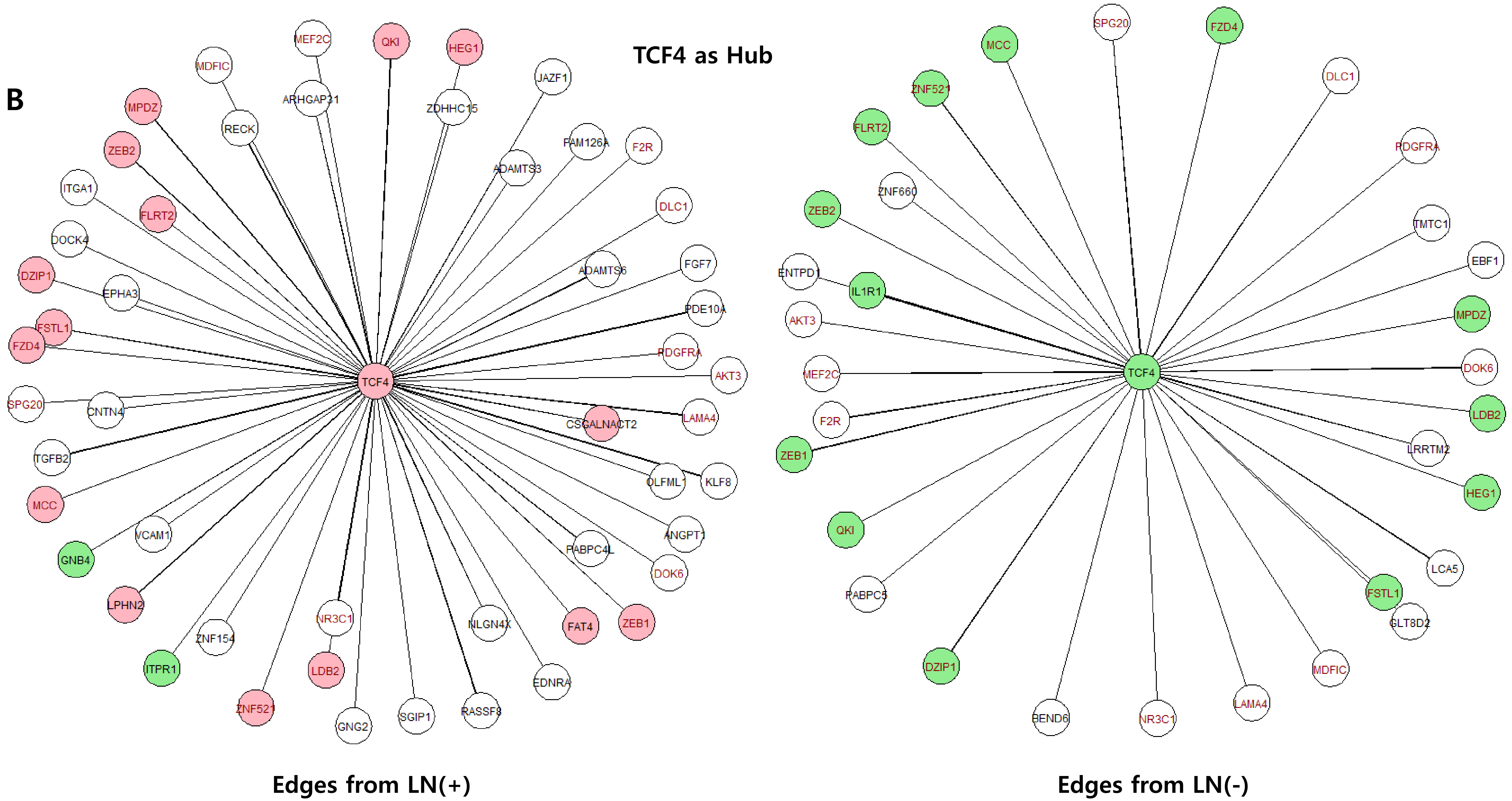
B
TCF4 as Hub
MEF2C
QKI
HEG1
MDFIC
ARHGAP31
ZDHHC15
JAZF1
MPDZ
RECK
FAM126A
F2R
ZEB2
ADAMTS3
ITGA1
FLRT2
DLC1
DOCK4
ADAMTS6
FGF7
DZIP1
EPHA3
PDE10A
FSTL1
FZD4
PDGFRA
TCF4
AKT3
SPG20
CNTN4
LAMA4
CSGALNACT2
TGFB2
OLFML1
KLF8
MCC
ANGPT1
VCAM1
PABPC4L
GNB4
DOK6
LPHN2
NR3C1
NLGN4X
FAT4
ZEB1
ZNF154
ITPR1
LDB2
EDNRA
ZNF521
GNG2
SGIP1
RASSF8
Edges from LN(+)
SPG20
FZD4
MCC
DLC1
ZNF521
FLRT2
PDGFRA
ZNF660
ZEB2
TMTC1
EBF1
ENTPD1
IL1R1
MPDZ
AKT3
TCF4
MEF2C
DOK6
LDB2
F2R
ZEB1
LRRTM2
HEG1
QKI
LCA5
PABPC5
FSTL1
GLT8D2
DZIP1
MDFIC
LAMA4
BEND6
NR3C1
Edges from LN(-)

C

SECISBP2L as Hub

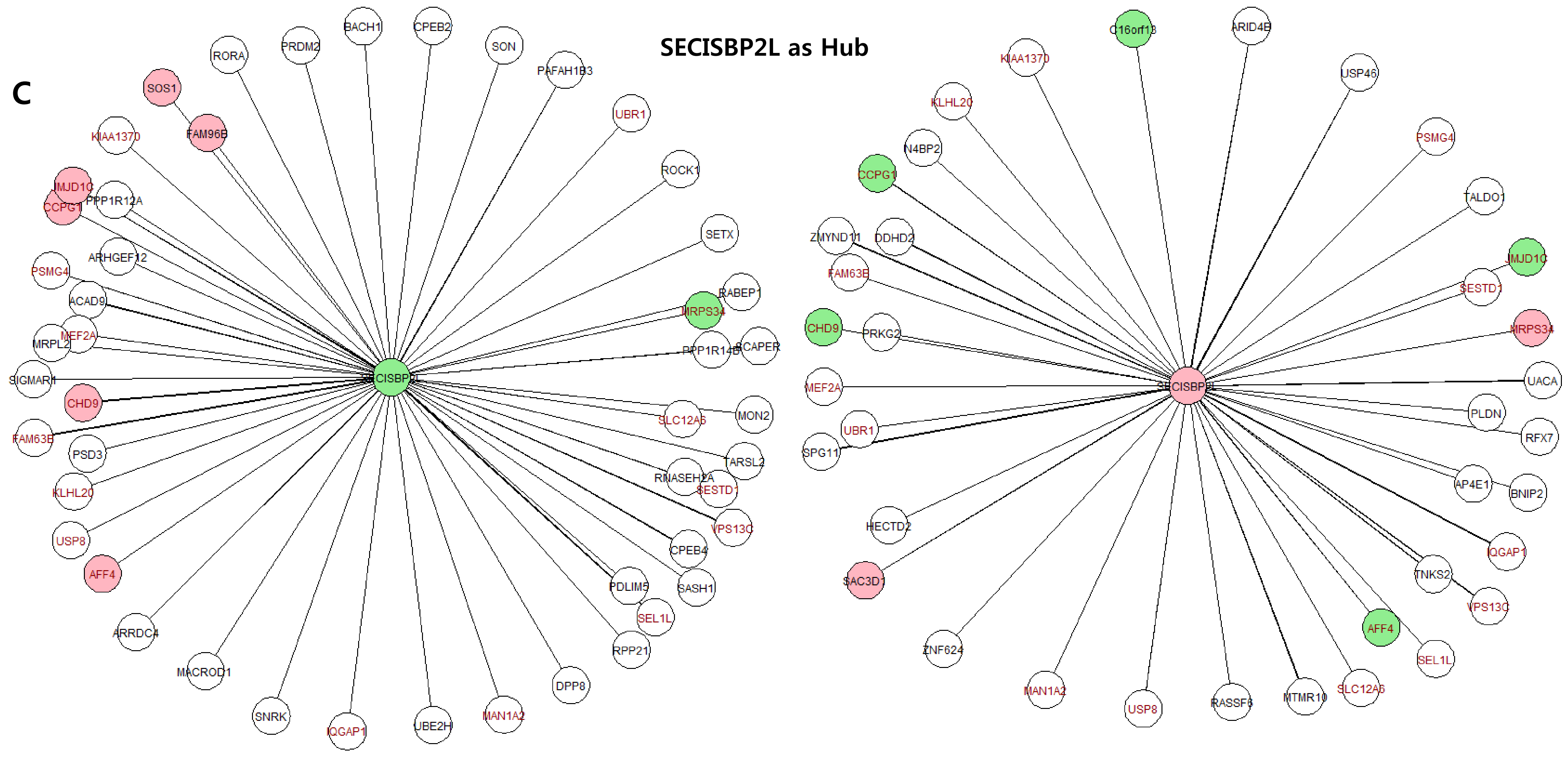

Edges from LN(+)

Edges from LN(-)

TPI1P3 as Hub

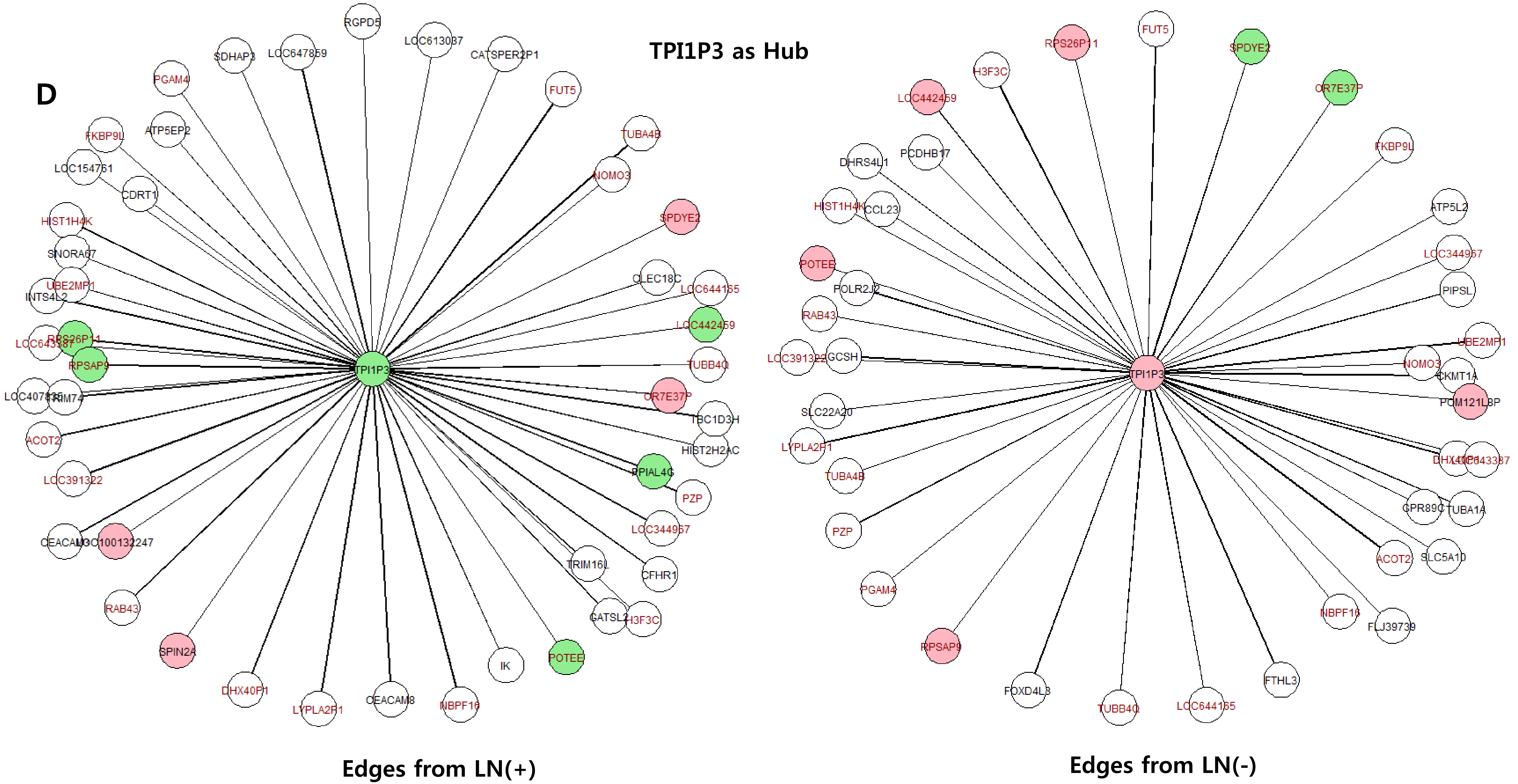

E

VIM as Hub

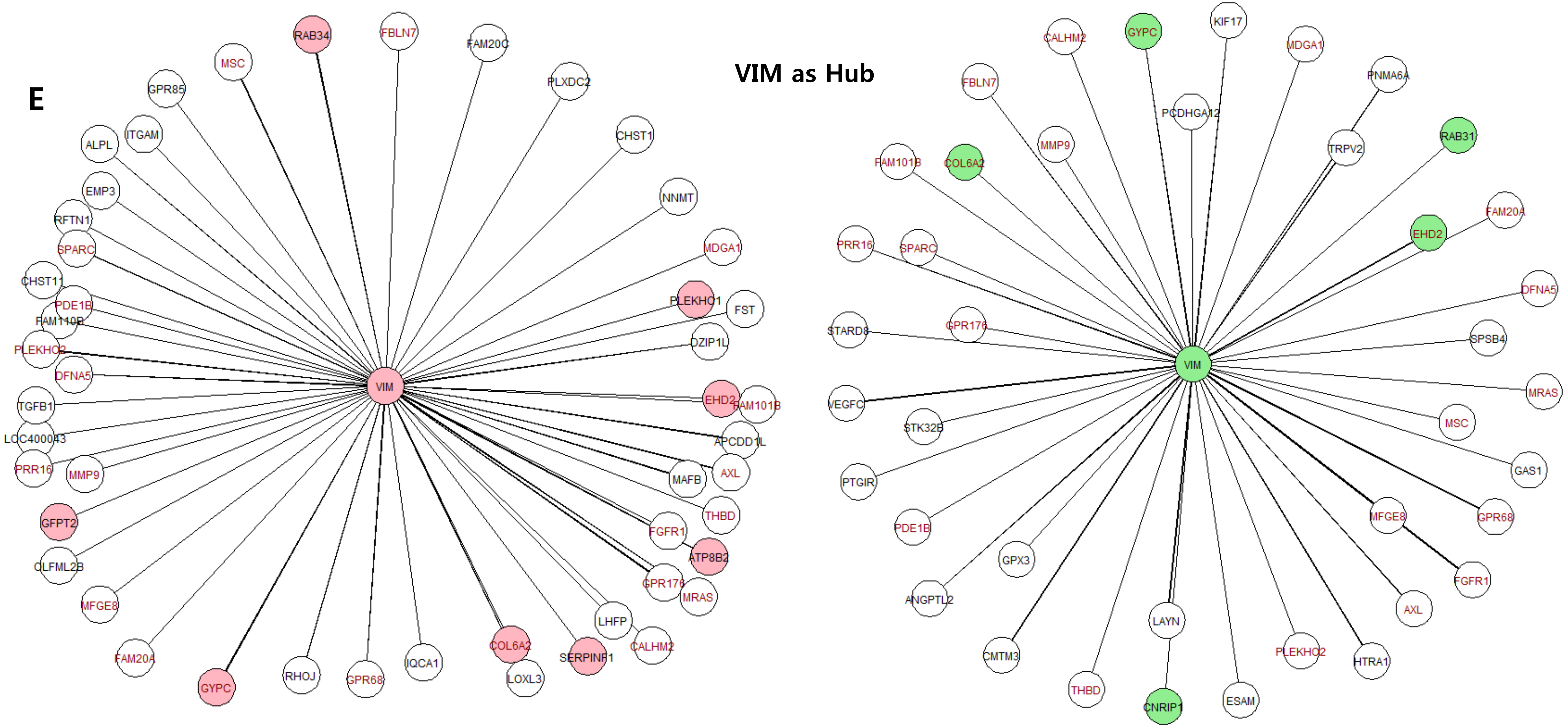

Edges from LN(+)

Edges from LN(-)

F

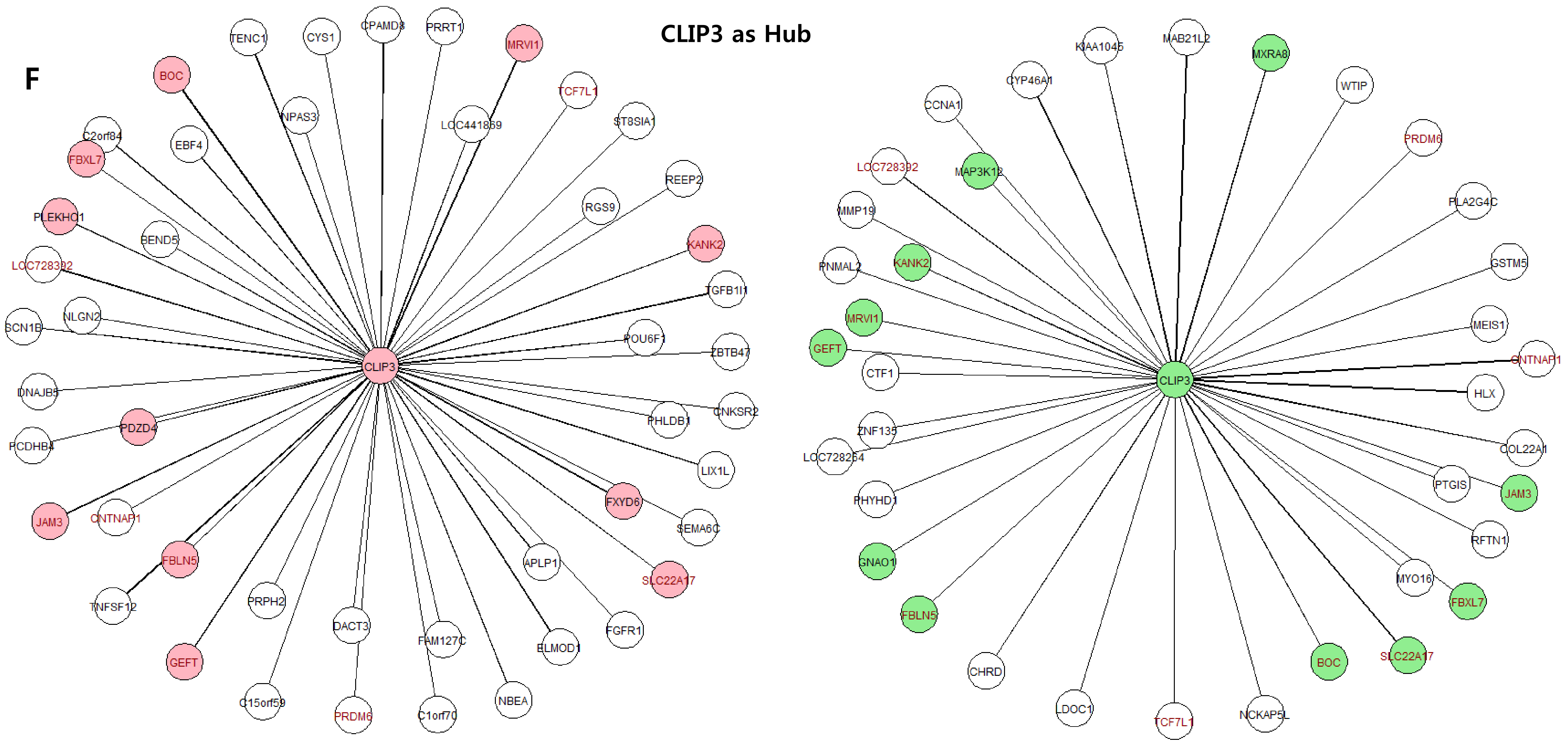

Edges from LN(+)

Edges from LN(-)

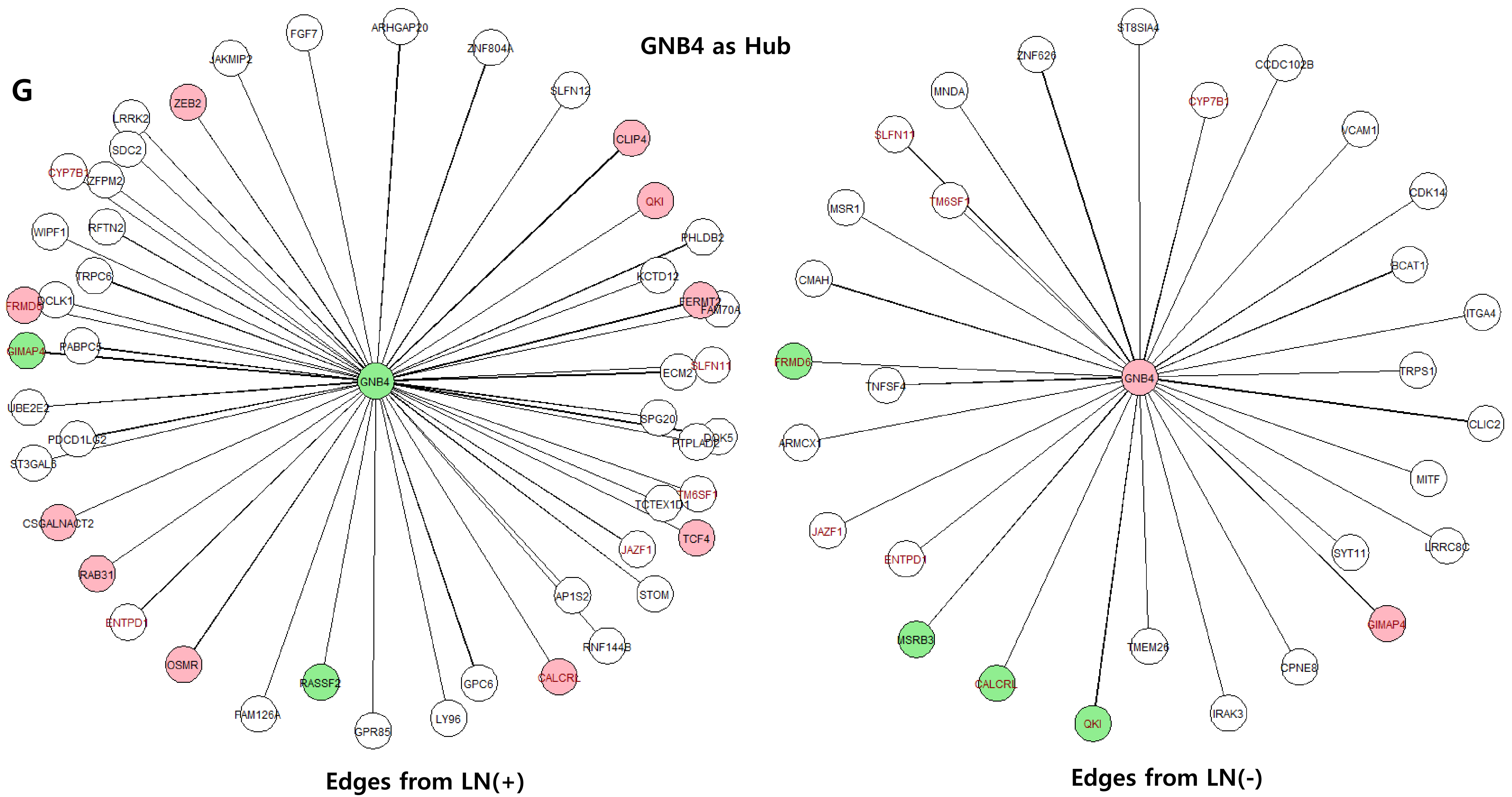

G
GNB4 as Hub
GNB4
FGF7
ARHGAP20
ZNF804A
JAKMIP2
SLFN12
ZEB2
LRRK2
CLIP4
SDC2
CYP7B1
ZFPM2
QKI
PHLDB2
WIPF1
RFTN2
KCTD12
TRPC6
FERMT2
FAM70A
FRMD6
DCLK1
GIMAP4
PABPC5
SLFN11
ECM2
UBE2E2
SPG20
PDCD1LG2
PTPLAD2
DOK5
ST3GAL6
TM6SF1
TCTEX1D1
CSGALNACT2
TCF4
JAZF1
RAB31
AP1S2
STOM
ENTPD1
RNF144B
OSMR
CALCRL
RASSF2
GPC6
FAM126A
LY96
GPR85
Edges from LN(+)
ST8SIA4
ZNF626
CCDC102B
MNDA
CYP7B1
SLFN11
VCAM1
TM6SF1
MSR1
CDK14
BCAT1
CMAH
ITGA4
FRMD6
TNFSF4
TRPS1
CLIC2
ARMCX1
MITF
JAZF1
SYT11
LRRC8C
ENTPD1
GIMAP4
MSRB3
TMEM26
CPNE8
CALCRL
IRAK3
QKI
Edges from LN(-)

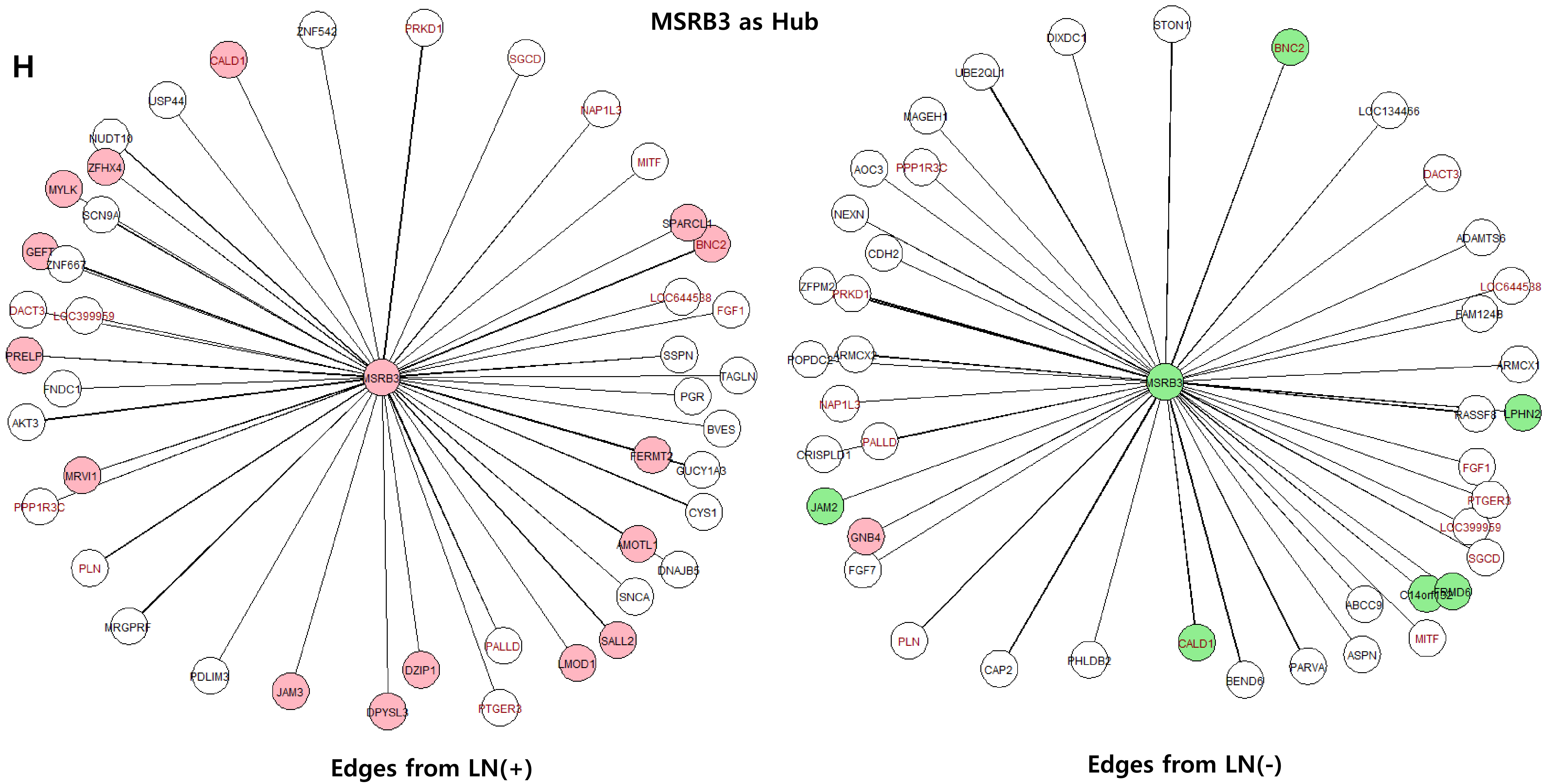

H
MSRB3 as Hub
ZNF542
PRKD1
CALD1
SGCD
USP44
NAP1L3
NUDT10
MITF
ZFHX4
MYLK
SCN9A
SPARCL1
BNC2
GEFT
ZNF667
LOC644538
FGF1
DACT3
LOC399959
PRELP
SSPN
TAGLN
MSRB3
FNDC1
PGR
AKT3
BVES
FERMT2
GUCY1A3
MRVI1
PPP1R3C
CYS1
AMOTL1
PLN
DNAJB5
SNCA
MRGPRF
SALL2
PALLD
LMOD1
PDLIM3
DZIP1
JAM3
PTGER3
DPYSL3
Edges from LN(+)
STON1
DIXDC1
BNC2
UBE2QL1
LOC134466
MAGEH1
DACT3
AOC3
PPP1R3C
NEXN
ADAMTS6
CDH2
LOC644538
ZFPM2
PRKD1
FAM124B
POPDC2
ARMCX2
ARMCX1
MSRB3
NAP1L3
RASSF8
LPHN2
PALLD
CRISPLD1
FGF1
JAM2
PTGER3
GNB4
LOC399959
SGCD
FGF7
C14orf132
FRMD6
ABCC9
MITF
PLN
ASPN
CAP2
PHLDB2
CALD1
PARVA
BEND6
Edges from LN(-)

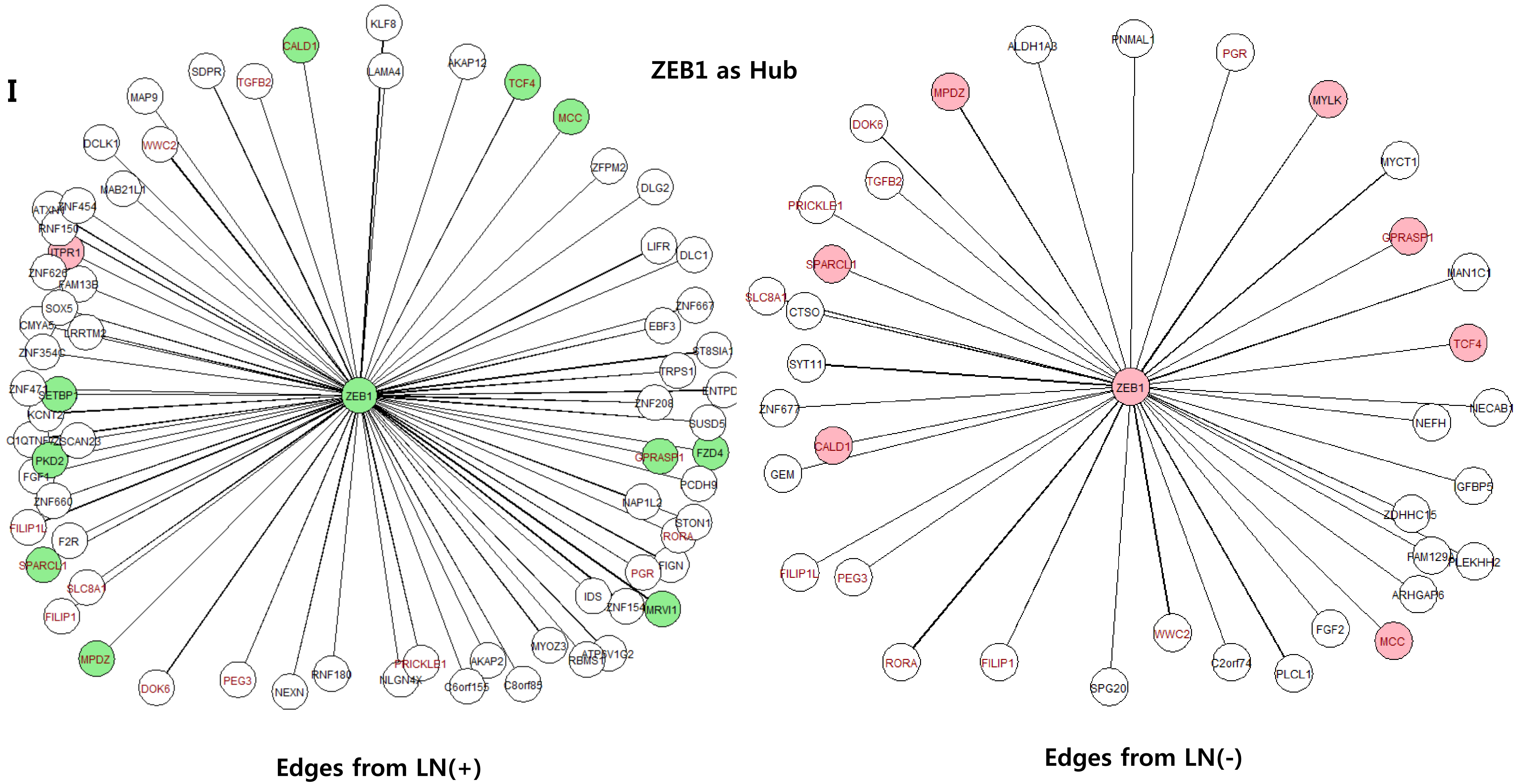

I
ZEB1 as Hub
Edges from LN(+)
Edges from LN(-)
ZEB1
KLF8
CALD1
LAMA4
SDPR
TGFB2
AKAP12
TCF4
MAP9
MCC
DCLK1
WWC2
ZFPM2
DLG2
MAB21L1
ATXN1
ZNF454
RNF150
ITPR1
LIFR
DLC1
ZNF626
FAM13B
SOX5
ZNF667
EBF3
CMYA5
LRRTM2
ST8SIA1
ZNF354C
TRPS1
ZNF47
SETBP1
ENTPD
ZNF208
KCNT2
SUSD5
C1QTNF7
ZSCAN23
GPRASP1
FZD4
PKD2
FGF1
PCDH9
ZNF660
NAP1L2
STON1
FILIP1L
RORA
F2R
FIGN
SPARCL1
PGR
SLC8A1
IDS
ZNF154
MRVI1
FILIP1
MPDZ
MYOZ3
ATP6V1G2
RBMS1
AKAP2
PRICKLE1
DOK6
PEG3
NEXN
RNF180
NLGN4X
C6orf155
C8orf85
ALDH1A3
PNMAL1
PGR
MPDZ
MYLK
DOK6
MYCT1
TGFB2
PRICKLE1
GPRASP1
SPARCL1
MAN1C1
SLC8A1
CTSO
TCF4
SYT11
ZEB1
NECAB1
ZNF677
NEFH
CALD1
GEM
GFBP5
ZDHHC15
FAM129A
PLEKHH2
FILIP1L
PEG3
ARHGAP6
FGF2
MCC
WWC2
RORA
FILIP1
C2orf74
PLCL1
SPG20

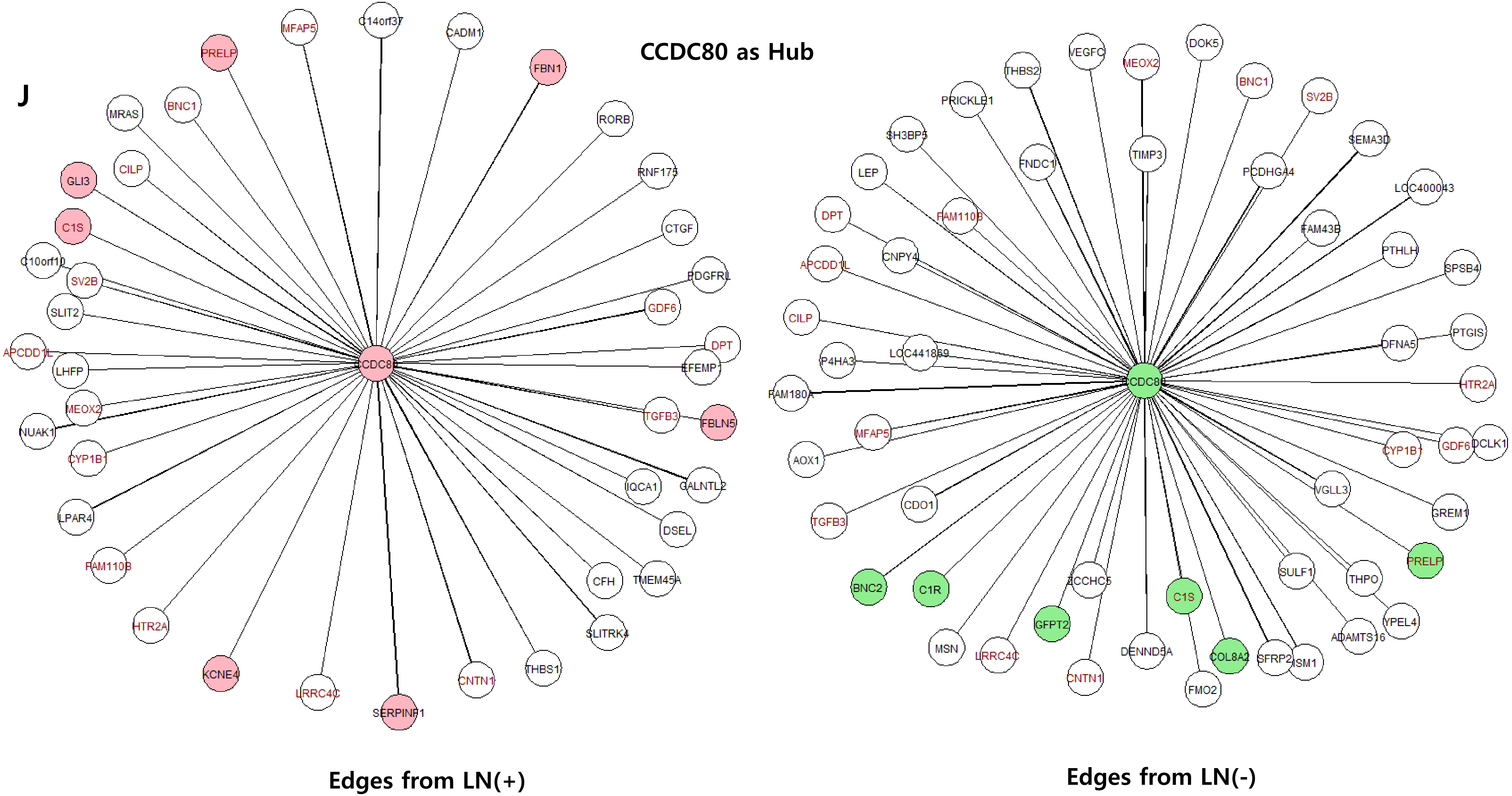
J
CCDC80 as Hub
Edges from LN(+)
Edges from LN(-)
CCDC80
PRELP
MFAP5
C14orf37
CADM1
FBN1
RORB
RNF175
CTGF
PDGFRL
GDF6
DPT
EFEMP1
TGFB3
FBLN5
GALNTL2
IQCA1
DSEL
TMEM45A
CFH
SLITRK4
THBS1
CNTN1
SERPINF1
LRRC4C
KCNE4
HTR2A
FAM110B
LPAR4
CYP1B1
NUAK1
MEOX2
LHFP
APCDD1L
SLIT2
SV2B
C10orf10
C1S
GLI3
CILP
MRAS
BNC1
VEGFC
MEOX2
DOK5
THBS2
BNC1
SV2B
PRICKLE1
TIMP3
SEMA3D
SH3BP5
FNDC1
PCDHGA4
LEP
LOC400043
DPT
FAM110B
FAM43B
CNPY4
PTHLH
APCDD1L
SPSB4
CILP
PTGIS
DFNA5
P4HA3
LOC441869
FAM180A
HTR2A
MFAP5
GDF6
DCLK1
CYP1B1
AOX1
VGLL3
CDO1
GREM1
TGFB3
PRELP
SULF1
THPO
BNC2
C1R
ZCCHC5
C1S
YPEL4
GFPT2
ADAMTS16
MSN
LRRC4C
DENND5A
COL8A2
SFRP2
ISM1
CNTN1
FMO2

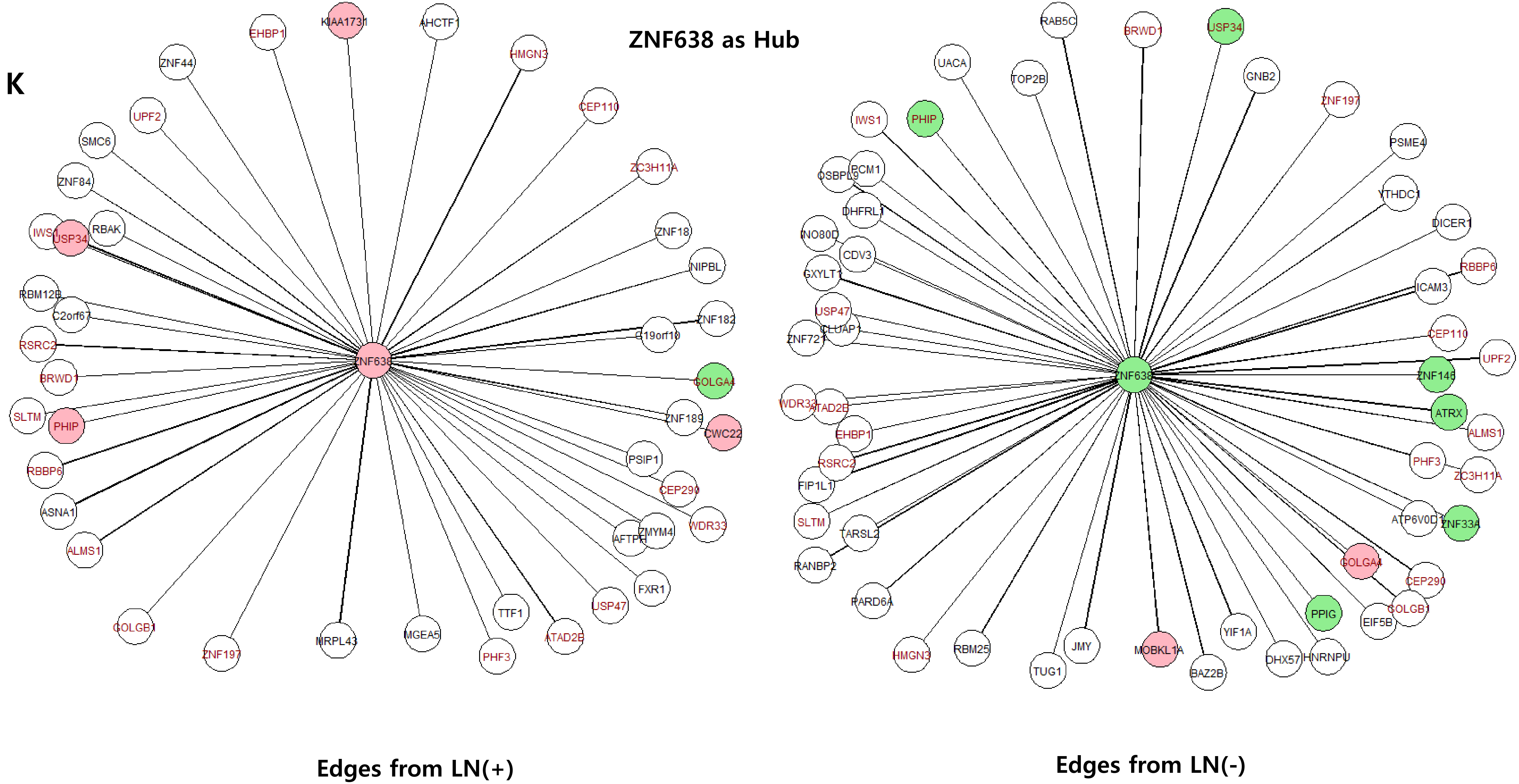
K
ZNF638 as Hub
ZNF638
EHBP1
KIAA1731
AHCTF1
HMGN3
CEP110
ZC3H11A
ZNF18
NIPBL
ZNF182
C19orf10
GOLGA4
ZNF189
CWC22
PSIP1
CEP290
WDR33
ZMYM4
AFTPH
FXR1
USP47
ATAD2B
TTF1
PHF3
MGEA5
MRPL43
ZNF197
GOLGB1
ALMS1
ASNA1
RBBP6
PHIP
SLTM
BRWD1
RSRC2
C2orf67
RBM12B
RBAK
USP34
IWS1
ZNF84
SMC6
UPF2
ZNF44
Edges from LN(+)
ZNF638
RAB5C
BRWD1
USP34
GNB2
ZNF197
PSME4
YTHDC1
DICER1
RBBP6
ICAM3
CEP110
UPF2
ZNF146
ATRX
ALMS1
PHF3
ZC3H11A
ATP6V0D1
ZNF334
GOLGA4
CEP290
GOLGB1
EIF5B
PPIG
HNRNPU
DHX57
YIF1A
BAZ2B
MOBKL1A
JMY
TUG1
RBM25
HMGN3
PARD6A
RANBP2
TARSL2
SLTM
FIP1L1
RSRC2
EHBP1
ATAD2B
WDR33
ZNF721
CLUAP1
USP47
GXYLT1
CDV3
INO80D
DHFRL1
OSBPL9
PCM1
IWS1
PHIP
UACA
TOP2B
Edges from LN(-)

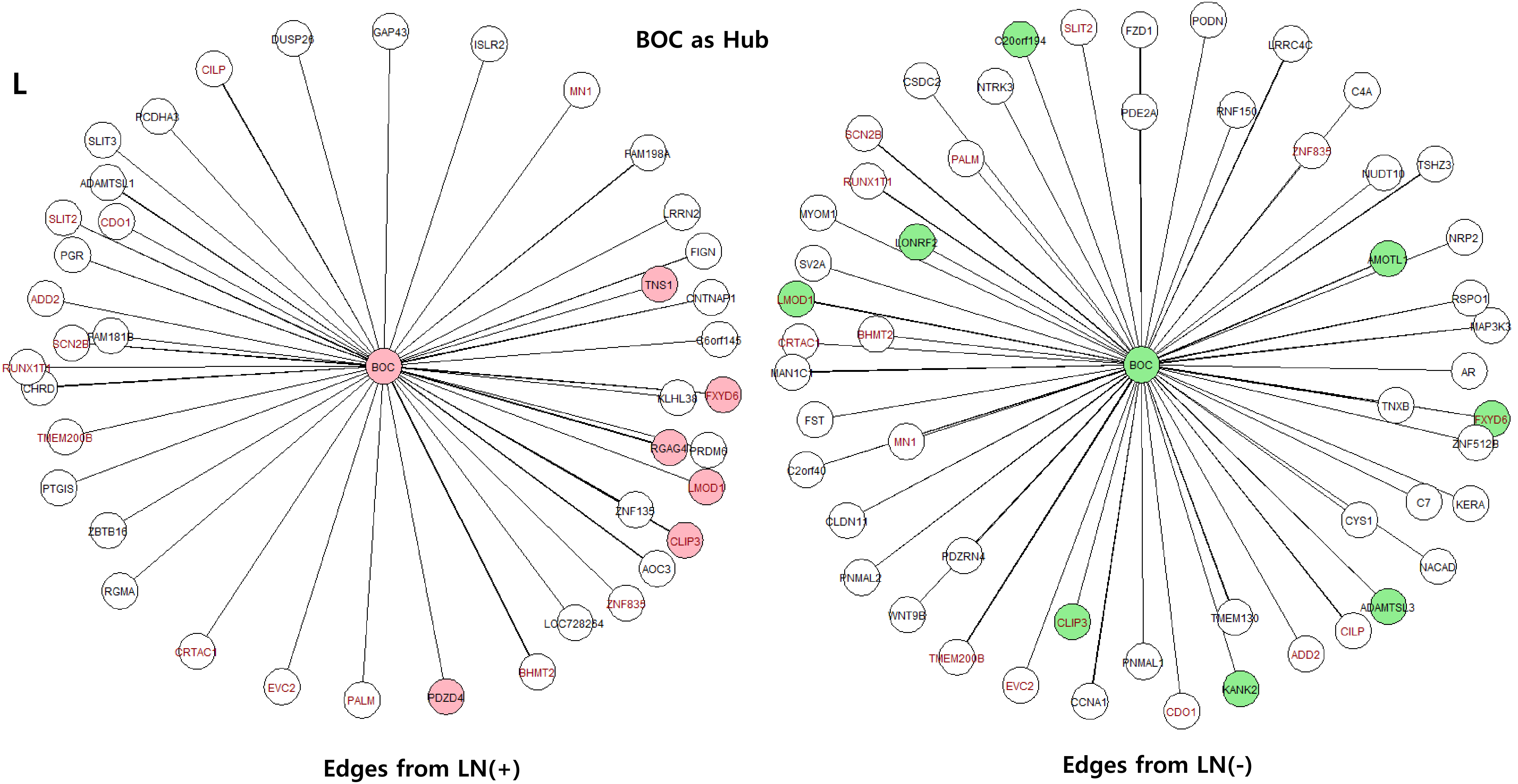
L
BOC as Hub
BOC
DUSP26
GAP43
ISLR2
CILP
MN1
PCDHA3
FAM198A
SLIT3
ADAMTSL1
LRRN2
SLIT2
CDO1
FIGN
PGR
TNS1
CNTNAP1
ADD2
C6orf145
SCN2B
FAM181B
RUNX1T1
CHRD
KLHL38
FXYD6
TMEM200B
RGAG4
PRDM6
PTGIS
LMOD1
ZBTB16
ZNF135
CLIP3
RGMA
AOC3
ZNF835
LOC728264
CRTAC1
BHMT2
EVC2
PALM
PDZD4
Edges from LN(+)
C20orf194
SLIT2
FZD1
PODN
LRRC4C
CSDC2
NTRK3
PDE2A
RNF150
C4A
SCN2B
PALM
ZNF835
NUDT10
TSHZ3
RUNX1T1
MYOM1
LONRF2
NRP2
SV2A
AMOTL1
LMOD1
RSPO1
BHMT2
MAP3K3
CRTAC1
BOC
AR
MAN1C1
TNXB
FXYD6
FST
MN1
ZNF512B
C2orf40
C7
KERA
CLDN11
CYS1
NACAD
PDZRN4
PNMAL2
ADAMTSL3
WNT9B
TMEM130
CILP
CLIP3
ADD2
TMEM200B
PNMAL1
EVC2
KANK2
CCNA1
CDO1
Edges from LN(-)

AHSA2 as Hub

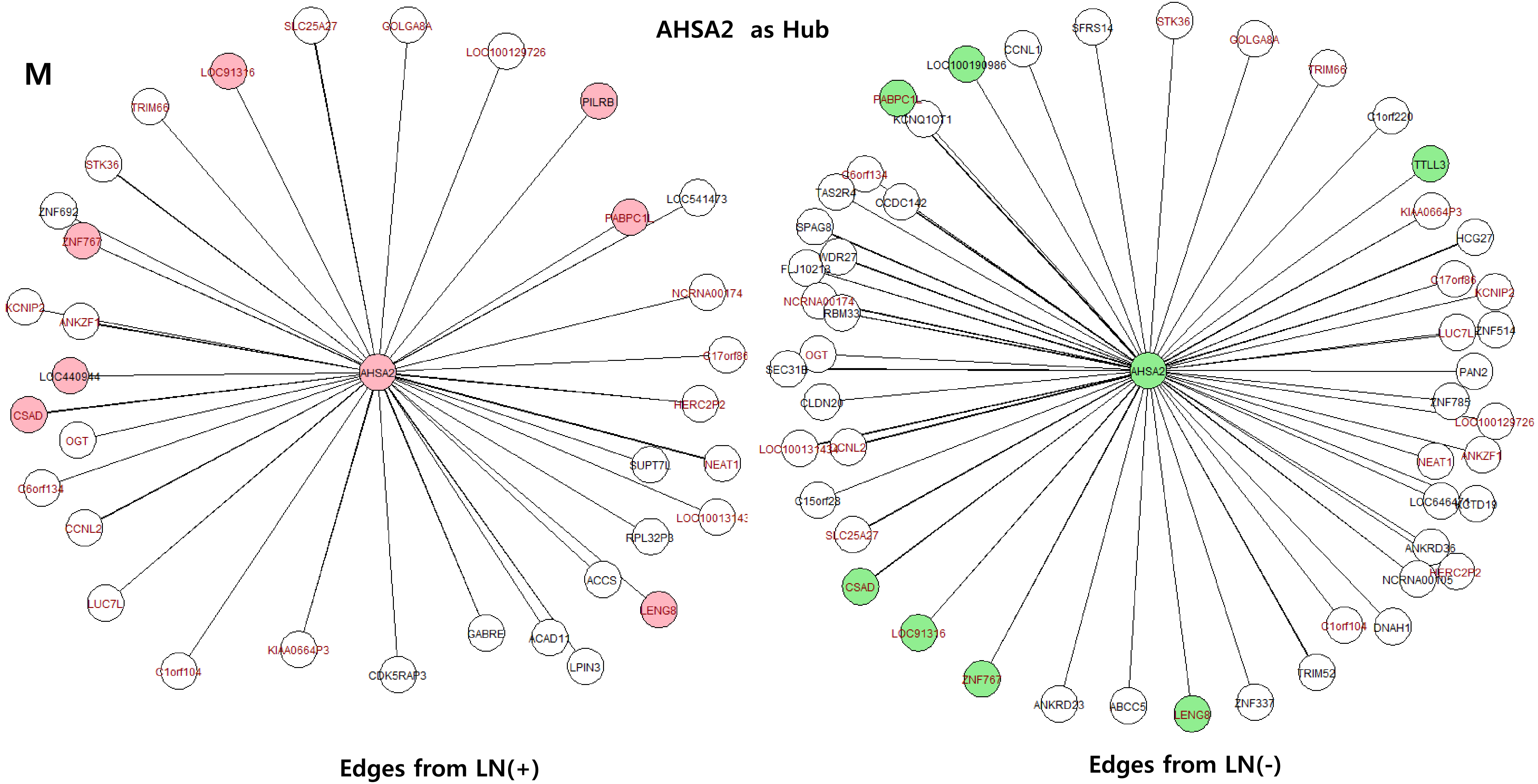
M
AHSA2
SLC25A27
GOLGA8A
LOC100129726
LOC91316
PILRB
TRIM66
STK36
LOC541473
PABPC1L
ZNF692
ZNF767
NCRNA00174
KCNIP2
ANKZF1
C17orf86
LOC440944
CSAD
HERC2P2
OGT
SUPT7L
NEAT1
C6orf134
CCNL2
RPL32P3
ACCS
LUC7L
LENG8
GABRE
ACAD11
KIAA0664P3
C1orf104
CDK5RAP3
LPIN3
Edges from LN(+)
SFRS14
STK36
CCNL1
GOLGA8A
LOC100190986
TRIM66
PABPC1L
KCNQ1OT1
C1orf220
TTLL3
C6orf134
TAS2R4
CCDC142
KIAA0664P3
SPAG8
HCG27
WDR27
FLJ10213
C17orf86
KCNIP2
NCRNA00174
RBM33
LUC7L
ZNF514
OGT
SEC31B
PAN2
CLDN20
ZNF785
LOC100129726
CCNL2
C15orf28
NEAT1
ANKZF1
LOC646471
KCTD19
SLC25A27
ANKRD36
CSAD
NCRNA00105
HERC2P2
LOC91316
C1orf104
DNAH1
ZNF767
TRIM52
ANKRD23
ABCC5
LENG8
ZNF337
Edges from LN(-)

N

PIKFYVE as Hub

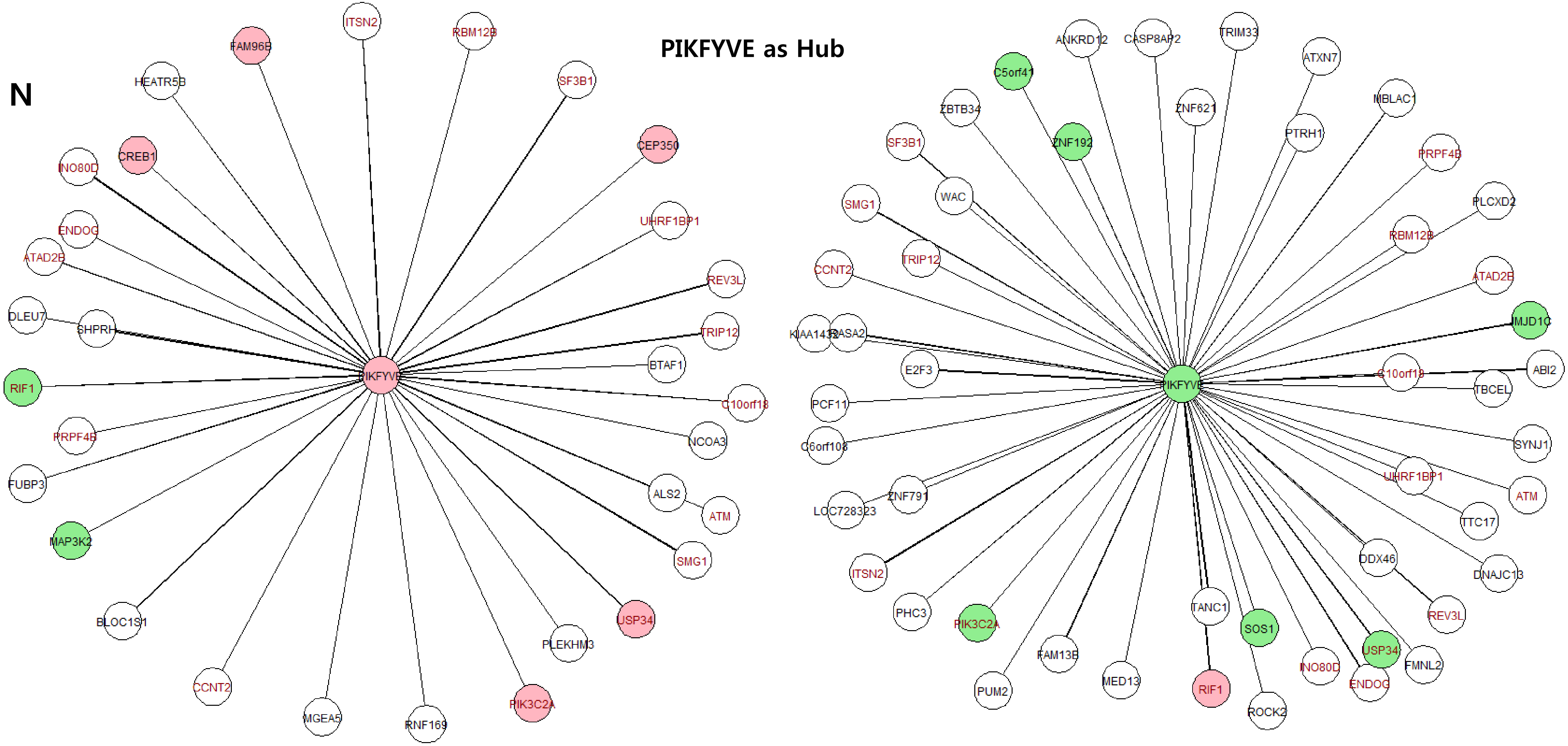

Edges from LN(+)

Edges from LN(-)

O

C1S as Hub

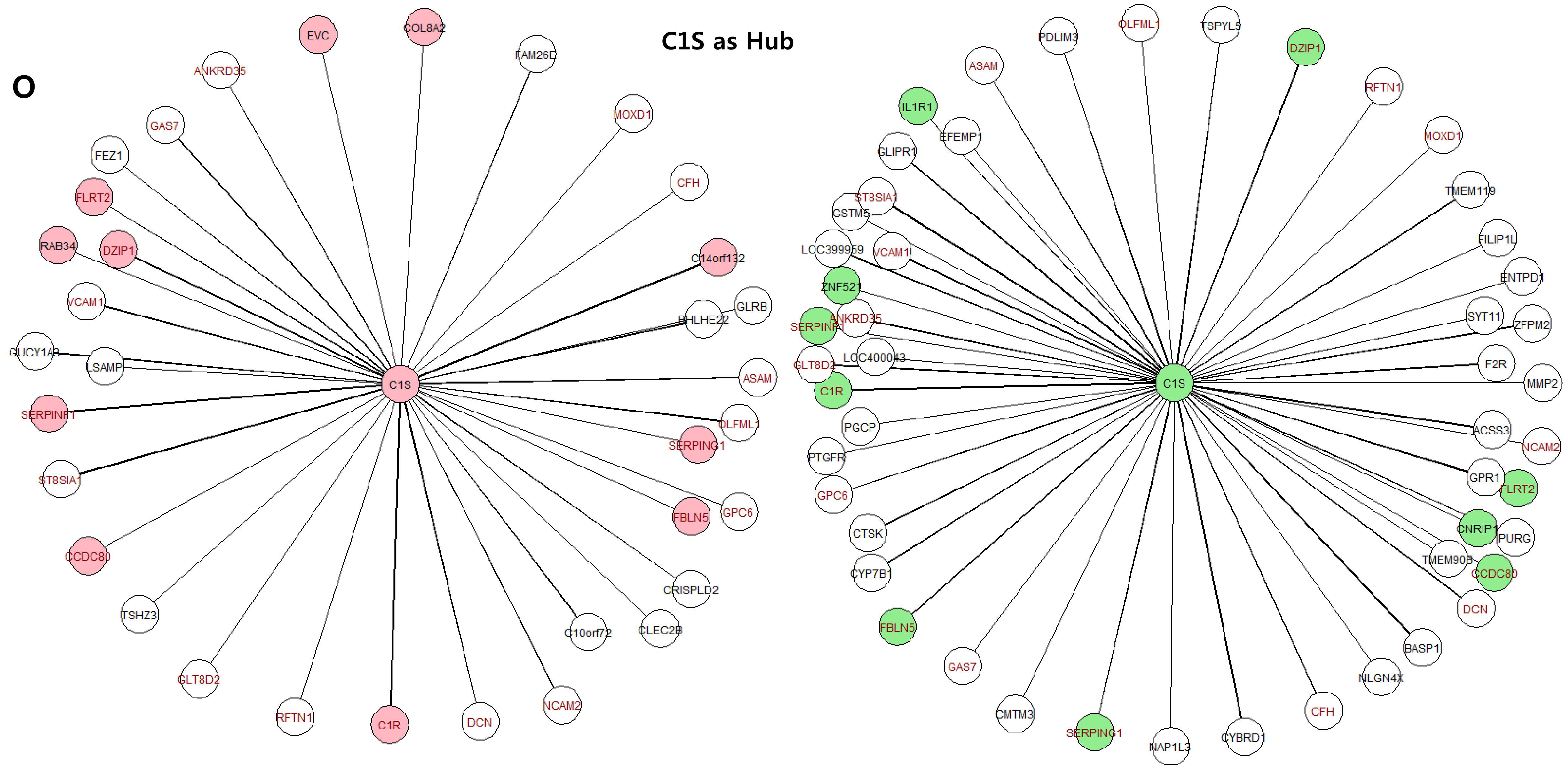

Edges from LN(+)

Edges from LN(-)

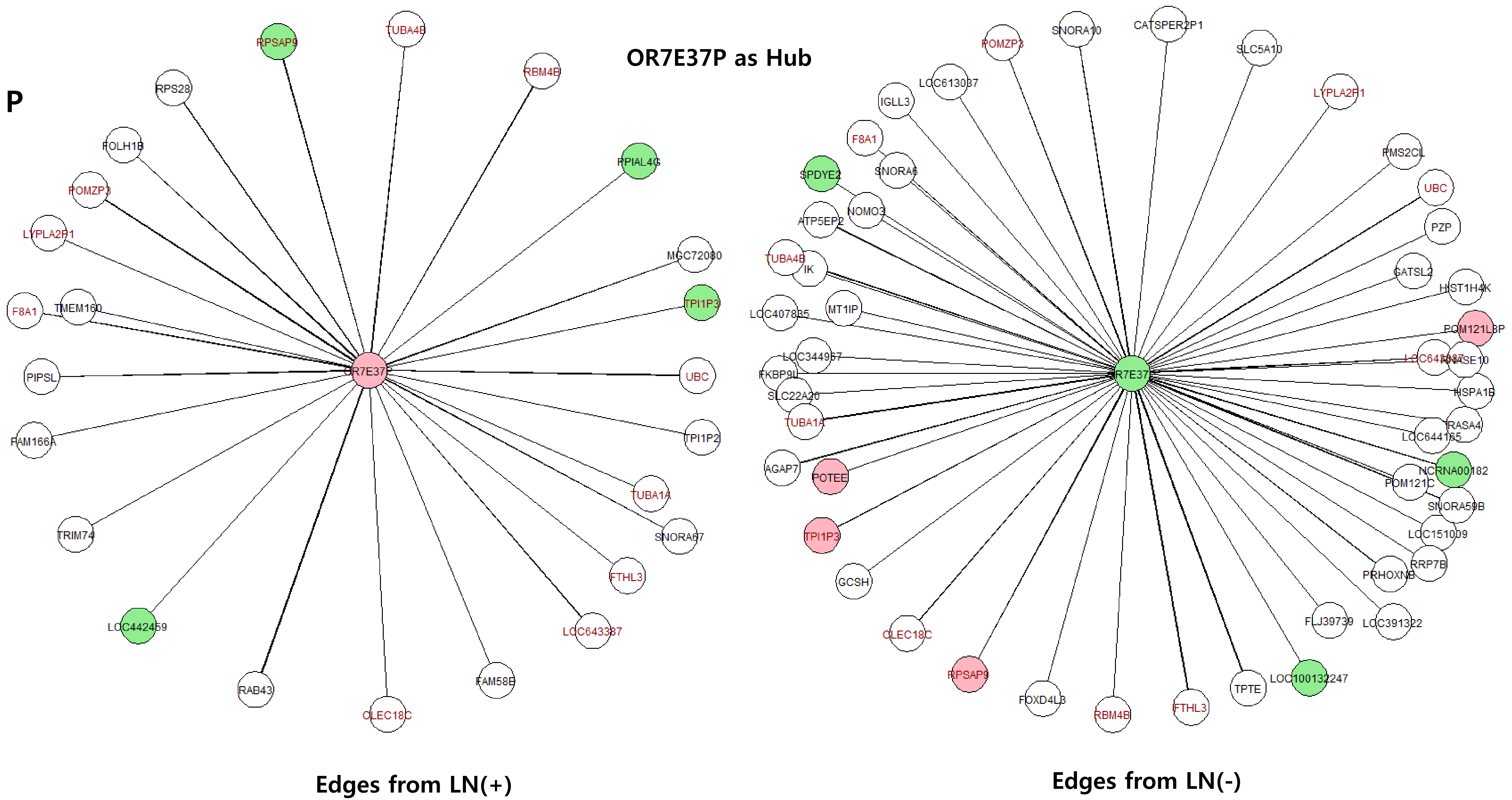

P
OR7E37P as Hub
OR7E37P
RPSAP9
TUBA4B
RBM4B
RPS28
FOLH1B
POMZP3
LYPLA2P1
F8A1
TMEM160
PIPSL
FAM166A
TRIM74
LOC442459
RAB43
CLEC18C
FAM58B
LOC643387
FTHL3
SNORA67
TUBA1A
TPI1P2
UBC
TPI1P3
MGC72080
PPIAL4G
Edges from LN(+)
SNORA10
CATSPER2P1
SLC5A10
LYPLA2P1
PMS2CL
UBC
PZP
GATSL2
HIST1H4K
POM121L8P
RNASE10
HSPA1B
RASA4
LOC644165
NCRNA00182
POM121C
SNORA59B
LOC151009
RRP7B
PRHOXNB
LOC391322
FLJ39739
LOC100132247
TPTE
FTHL3
RBM4B
FOXD4L3
RPSAP9
CLEC18C
GCSH
TPI1P3
POTEE
AGAP7
TUBA1A
SLC22A20
FKBP9L
LOC344967
LOC407835
MT1IP
IK
TUBA4B
ATP5EP2
NOMO3
SPDYE2
SNORA6
F8A1
IGLL3
LOC613037
POMZP3
Edges from LN(-)

XPO1 as Hub

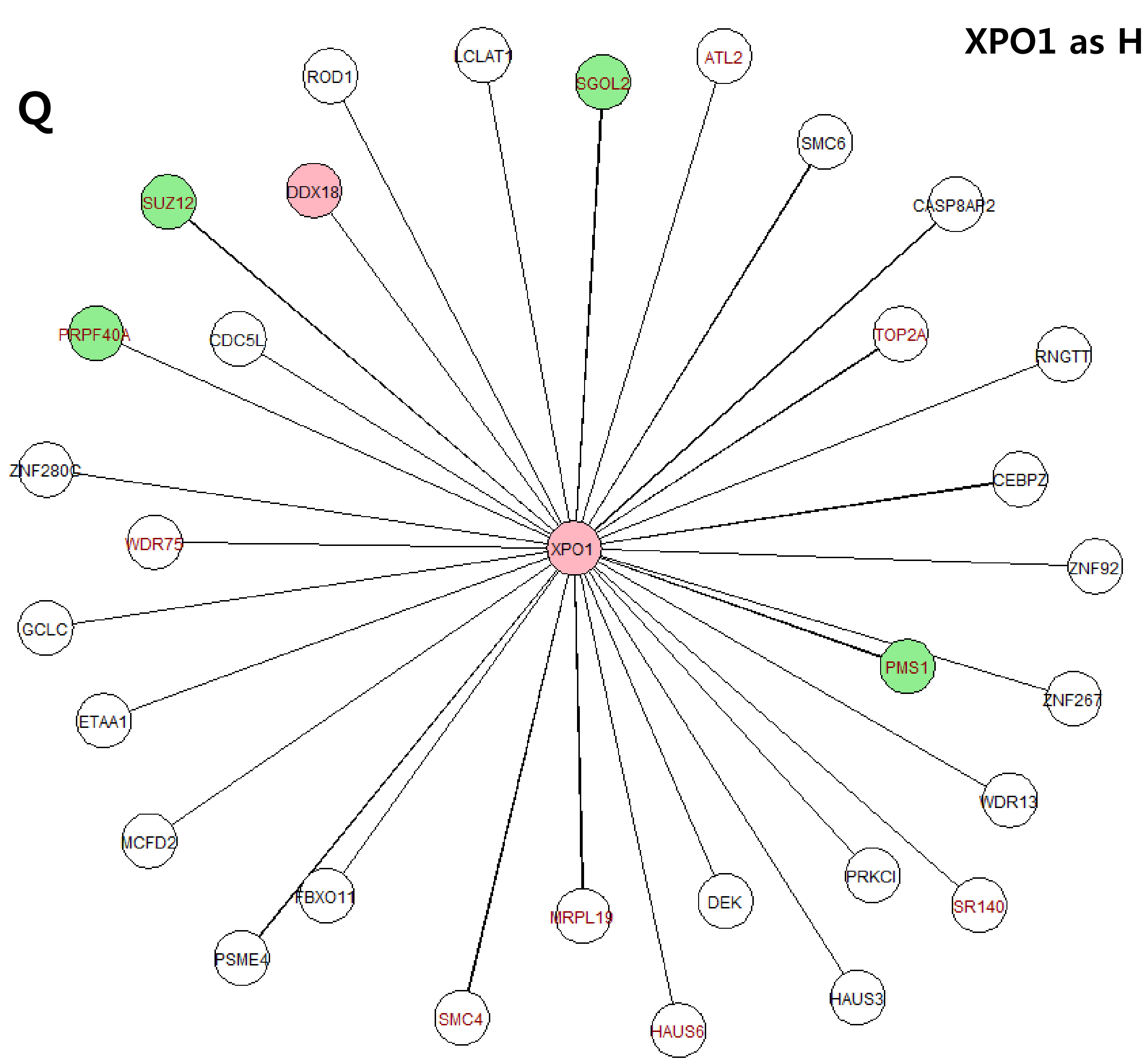

Edges from LN(+)

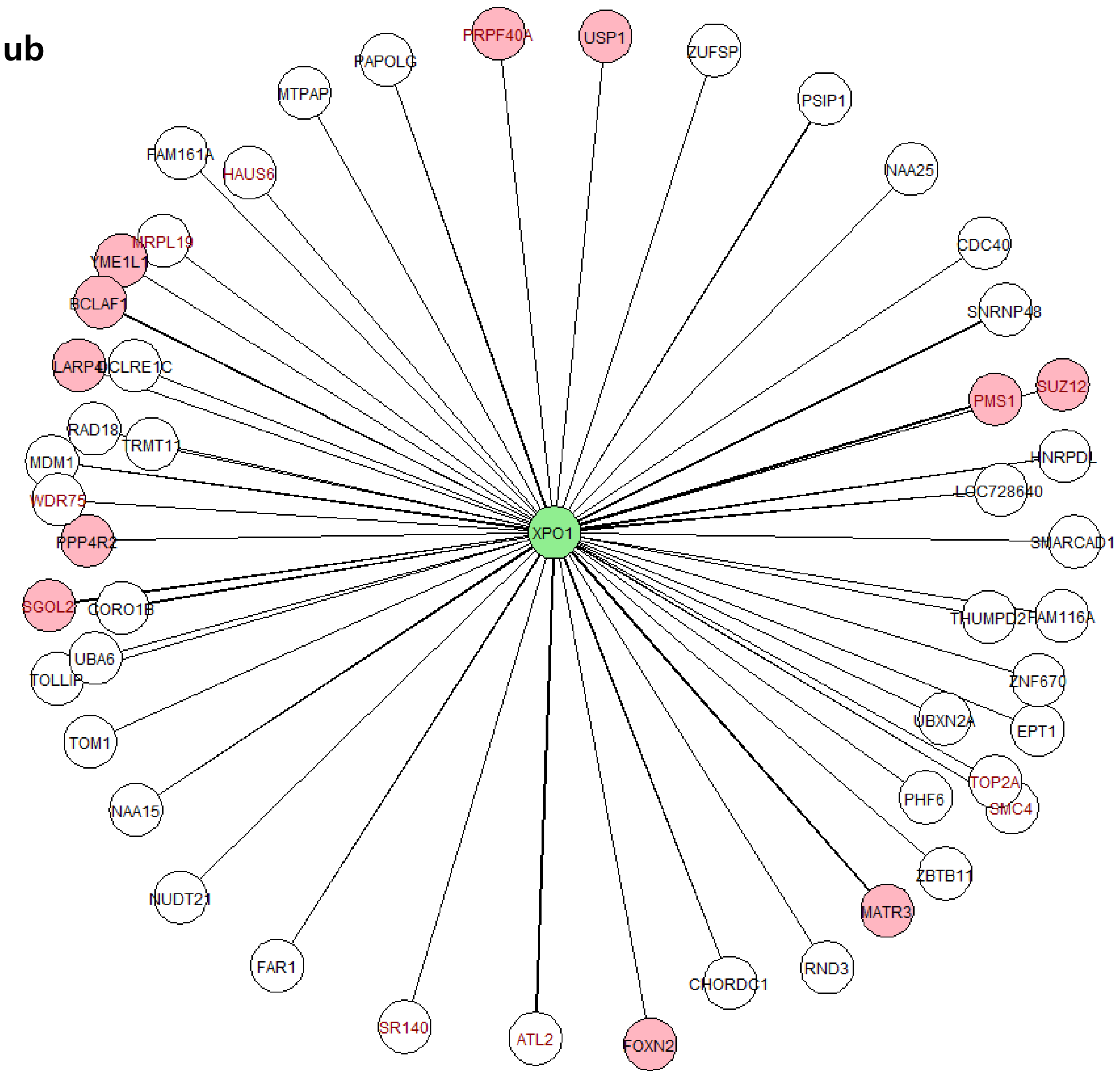

Edges from LN(-)

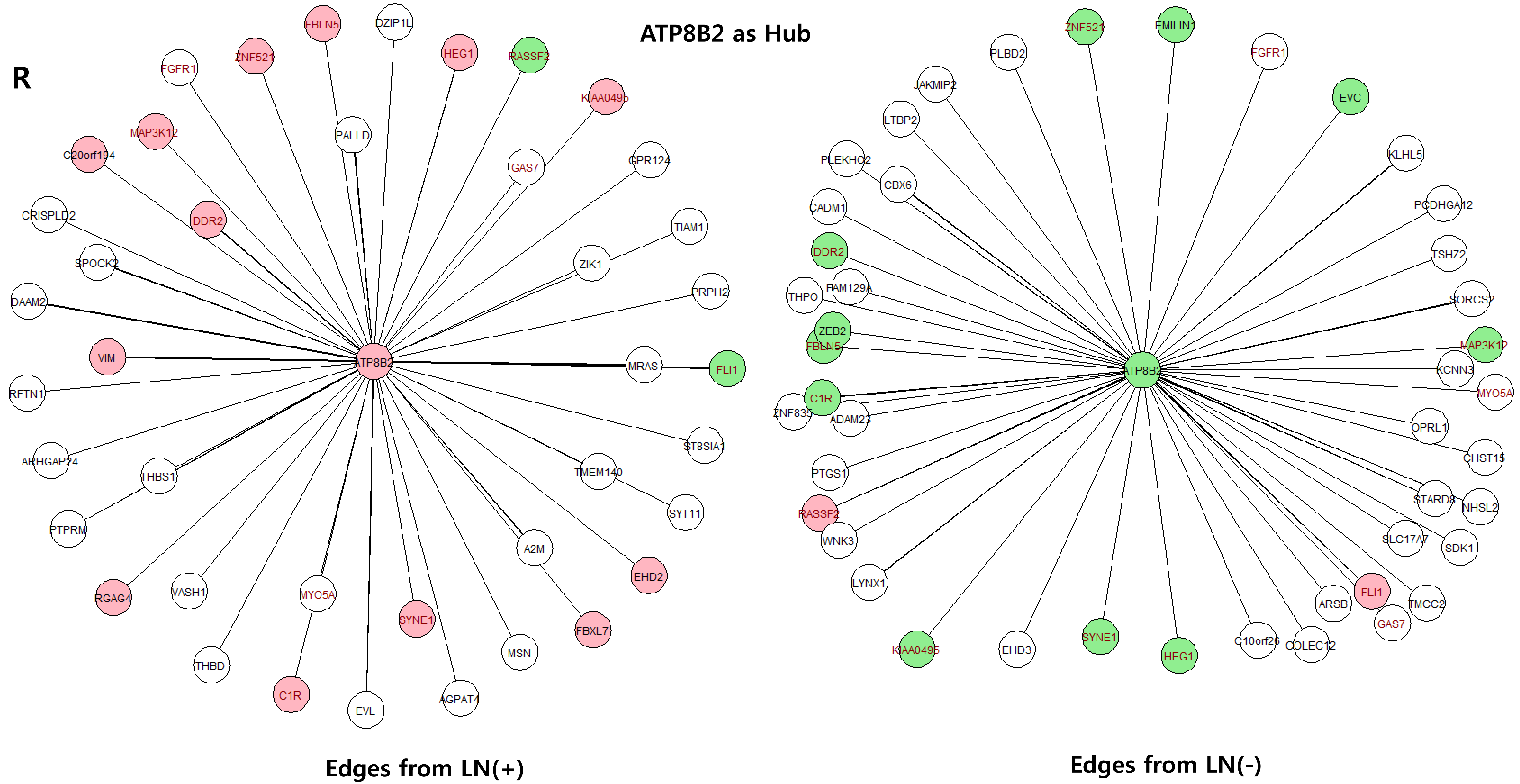

R
ATP8B2 as Hub
ATP8B2
FBLN5
DZIP1L
HEG1
RASSF2
ZNF521
FGFR1
KIAA0495
PALLD
MAP3K12
C20orf194
GAS7
GPR124
DDR2
CRISPLD2
TIAM1
ZIK1
SPOCK2
PRPH2
DAAM2
VIM
MRAS
FLI1
RFTN1
ARHGAP24
ST8SIA1
THBS1
TMEM140
SYT11
PTPRM
A2M
EHD2
RGAG4
VASH1
MYO5A
SYNE1
FBXL7
THBD
MSN
C1R
EVL
AGPAT4
Edges from LN(+)
ZNF521
EMILIN1
PLBD2
FGFR1
JAKMIP2
EVC
LTBP2
KLHL5
PLEKHO2
CBX6
CADM1
PCDHGA12
DDR2
TSHZ2
THPO
FAM129A
SORCS2
ZEB2
FBLN5
MAP3K12
ATP8B2
KCNN3
C1R
MYO5A
ZNF835
ADAM23
OPRL1
CHST15
PTGS1
STARD8
NHSL2
RASSF2
WNK3
SLC17A7
SDK1
LYNX1
FLI1
ARSB
TMCC2
GAS7
KIAA0495
EHD3
SYNE1
HEG1
C10orf26
COLEC12
Edges from LN(-)

TNS1 as Hub

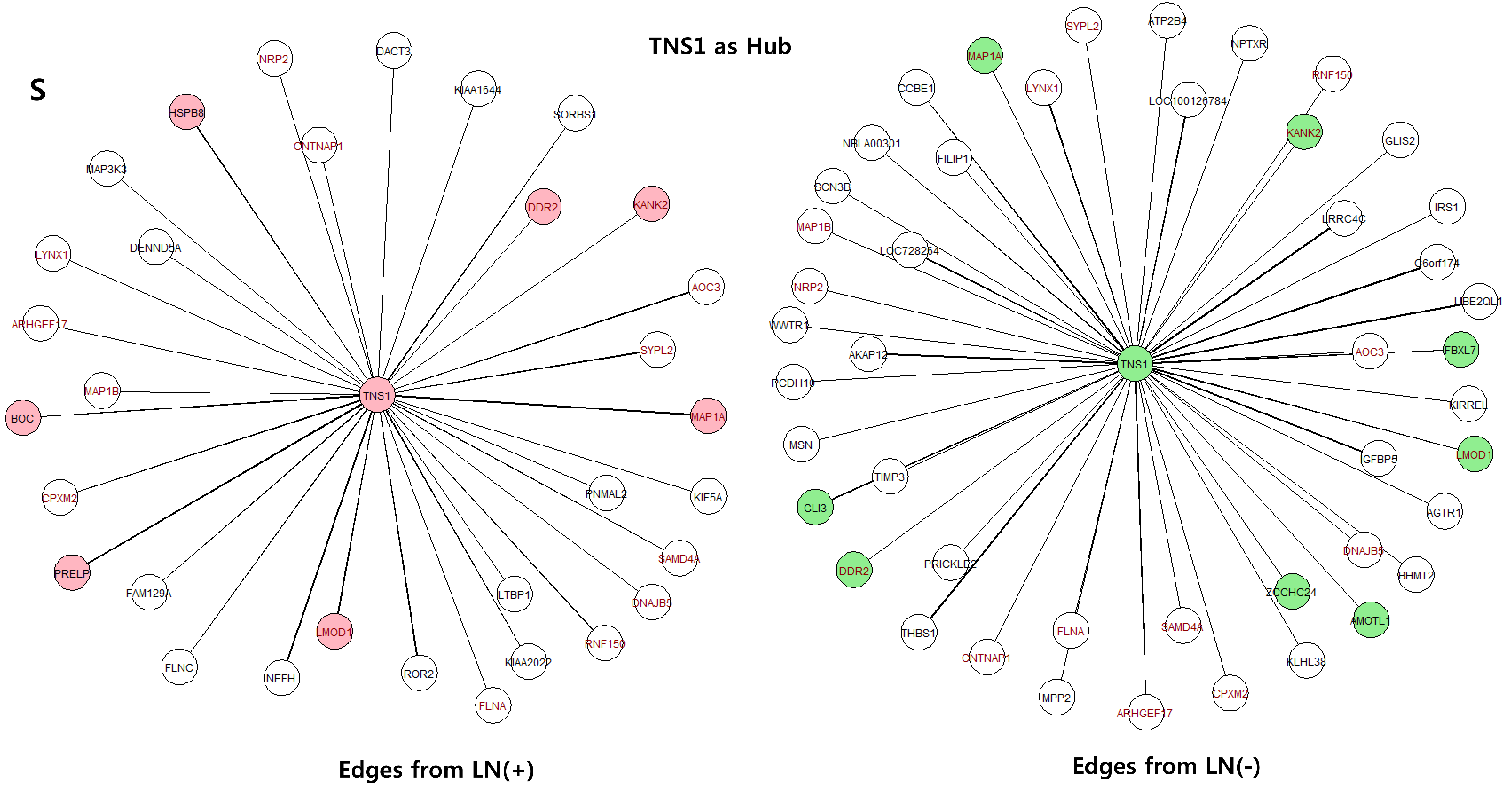
S
TNS1
NRP2
DACT3
KIAA1644
SORBS1
HSPB8
CNTNAP1
MAP3K3
DDR2
KANK2
LYNX1
DENND5A
AOC3
ARHGEF17
SYPL2
MAP1B
BOC
MAP1A
CPXM2
PNMAL2
KIF5A
SAMD4A
PRELP
FAM129A
LTBP1
DNAJB5
LMOD1
RNF150
KIAA2022
FLNC
NEFH
ROR2
FLNA
Edges from LN(+)
SYPL2
ATP2B4
MAP1A
NPTXR
RNF150
CCBE1
LYNX1
LOC100126784
NBLA00301
FILIP1
KANK2
GLIS2
SCN3B
IRS1
LRRC4C
MAP1B
LOC728264
C6orf174
NRP2
UBE2QL1
WWTR1
AKAP12
AOC3
FBXL7
PCDH10
KIRREL
MSN
GFBP5
LMOD1
TIMP3
GLI3
AGTR1
DDR2
PRICKLE2
DNAJB5
BHMT2
ZCCHC24
AMOTL1
THBS1
FLNA
SAMD4A
CNTNAP1
KLHL38
MPP2
CPXM2
ARHGEF17
Edges from LN(-)

VPS26A as Hub

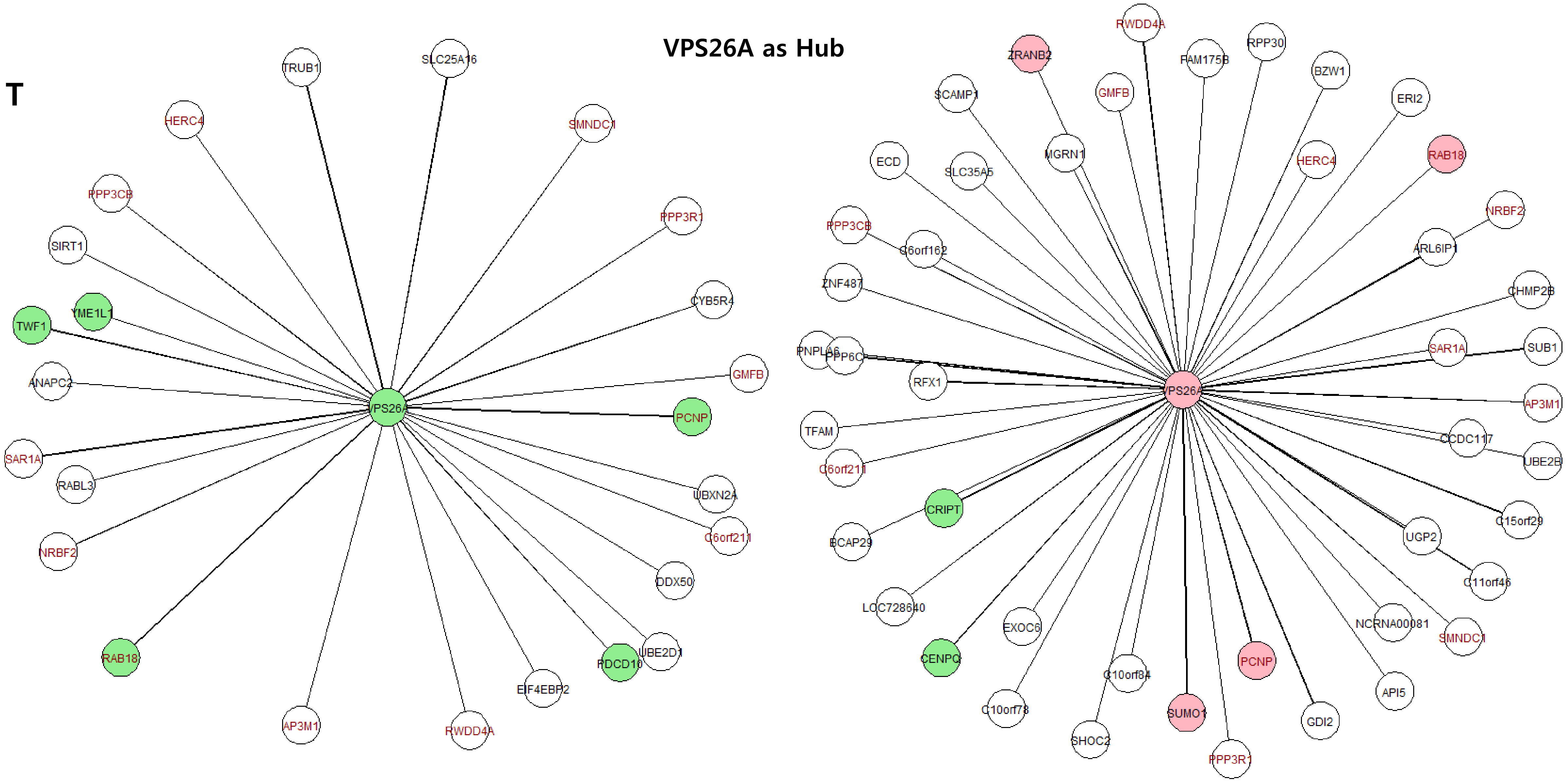

Edges from LN(+)

Edges from LN(-)

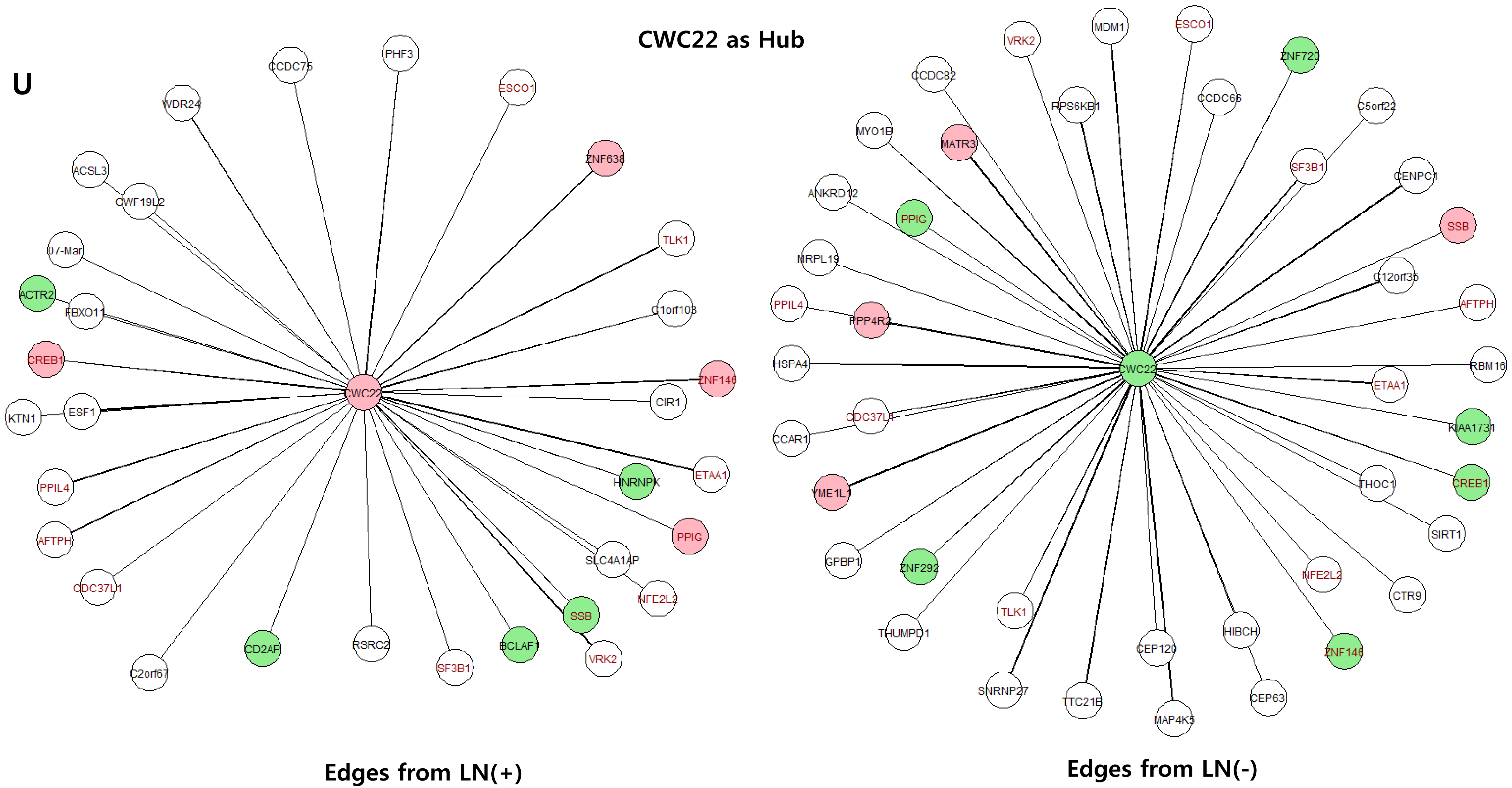
CWC22 as Hub
U
PHF3
CCDC75
ESCO1
WDR24
ZNF638
ACSL3
CWF19L2
TLK1
07-Mar
C1orf103
ACTR2
FBXO11
CREB1
CWC22
ZNF146
CIR1
KTN1
ESF1
ETAA1
HNRNPK
PPIL4
PPIG
AFTPH
SLC4A1AP
CDC37L1
NFE2L2
SSB
BCLAF1
VRK2
CD2AP
RSRC2
SF3B1
C2orf67
Edges from LN(+)
MDM1
ESCO1
VRK2
ZNF720
CCDC82
CCDC66
RPS6KB1
C5orf22
MYO1B
MATR3
SF3B1
ANKRD12
CENPC1
PPIG
SSB
MRPL19
C12orf35
PPIL4
AFTPH
PPP4R2
HSPA4
CWC22
RBM16
ETAA1
CDC37L1
KIAA1731
CCAR1
YME1L1
THOC1
CREB1
SIRT1
GPBP1
ZNF292
NFE2L2
CTR9
TLK1
THUMPD1
HIBCH
CEP120
ZNF146
SNRNP27
TTC21B
CEP63
MAP4K5
Edges from LN(-)

MATR3 as Hub

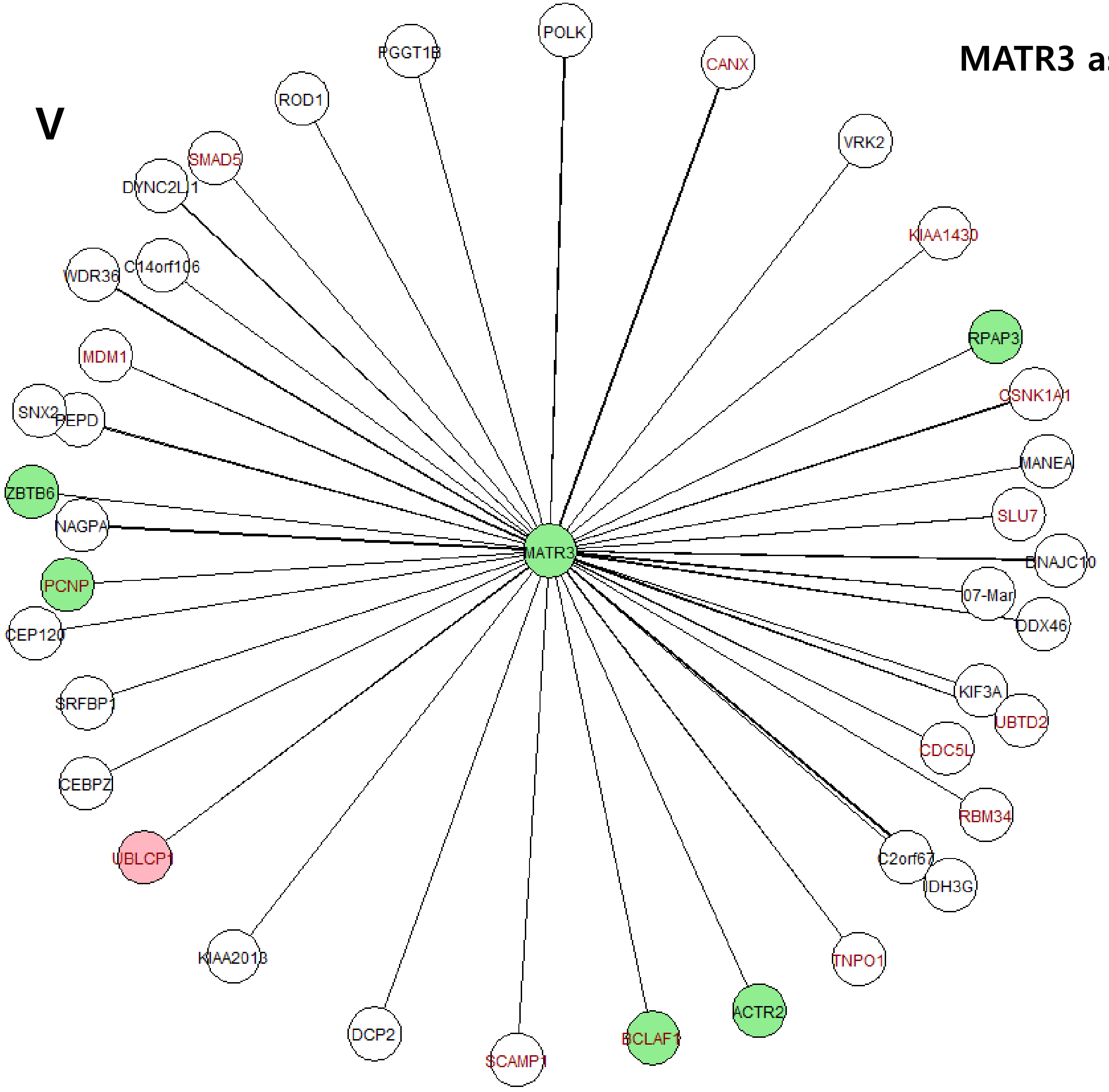

Edges from LN(+)

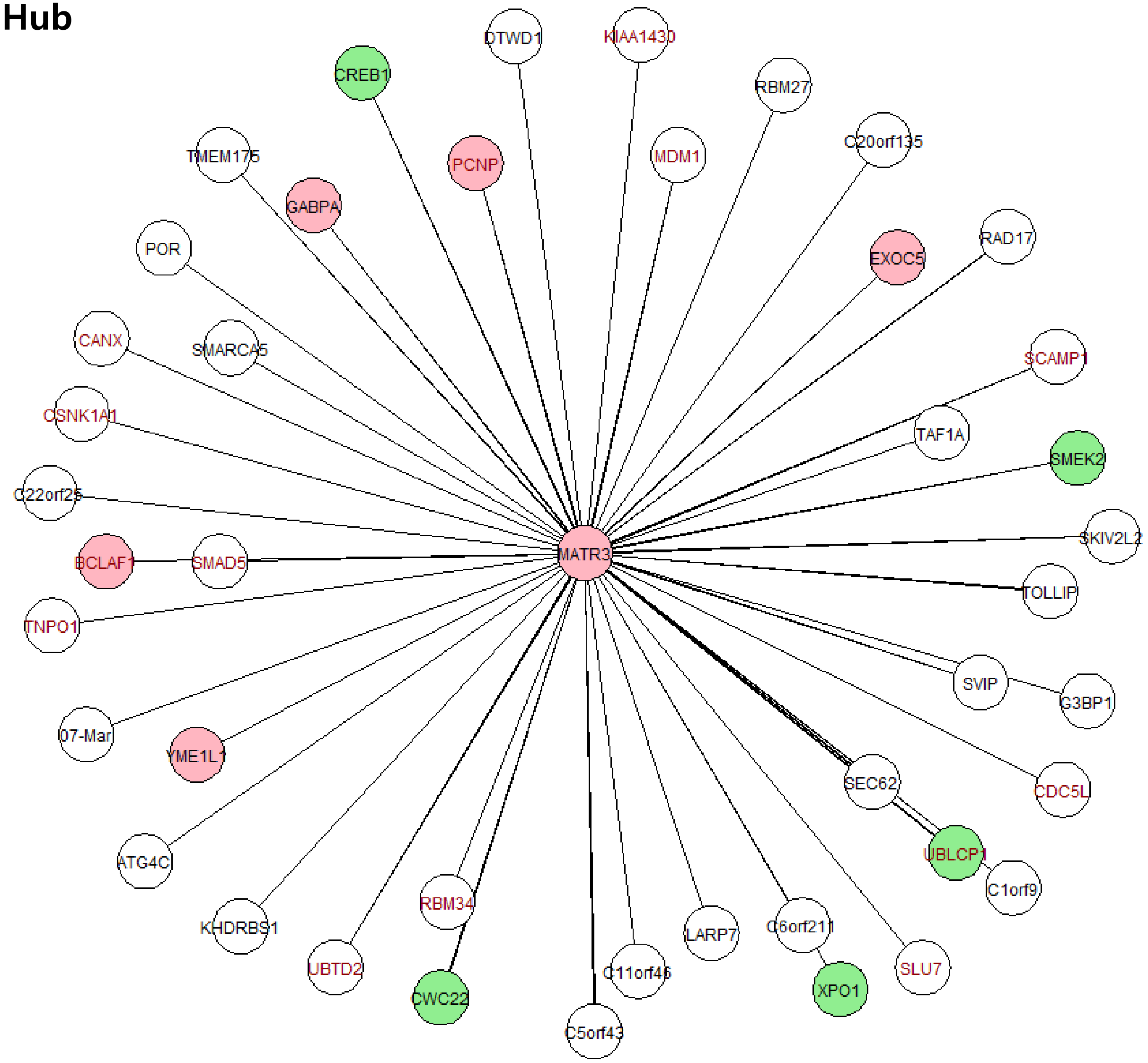

Edges from LN(-)

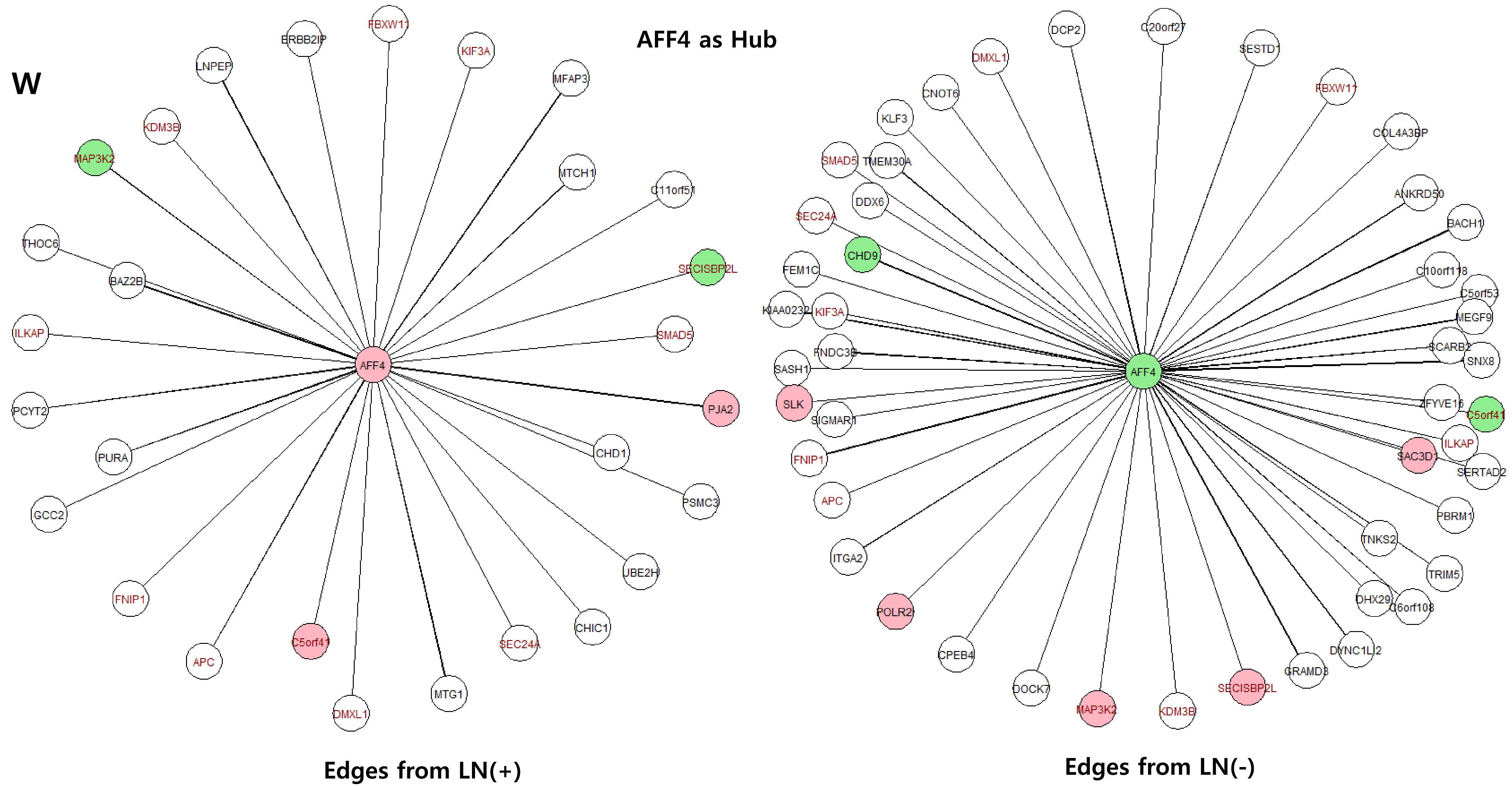
W
AFF4 as Hub
AFF4
FBXW11
ERBB2IP
KIF3A
LNPEP
MFAP3
KDM3B
MAP3K2
MTCH1
C11orf51
THOC6
BAZ2B
SECISBP2L
ILKAP
SMAD5
PCYT2
PJA2
PURA
CHD1
GCC2
PSMC3
UBE2H
FNIP1
CHIC1
APC
C5orf41
SEC24A
MTG1
DMXL1
Edges from LN(+)
DCP2
C20orf27
SESTD1
DMXL1
FBXW11
CNOT6
KLF3
COL4A3BP
SMAD5
TMEM30A
ANKRD50
DDX6
SEC24A
BACH1
CHD9
FEM1C
C10orf118
C5orf53
KIAA0232
KIF3A
MEGF9
SCARB2
FNDC3B
SNX8
SASH1
SLK
ZFYVE16
C5orf41
SIGMAR1
ILKAP
SAC3D1
FNIP1
SERTAD2
APC
PBRM1
TNKS2
ITGA2
TRIM5
POLR2
DHX29
C6orf108
DYNC1LI2
GRAMD3
CPEB4
DOCK7
SECISBP2L
MAP3K2
KDM3B
Edges from LN(-)

X

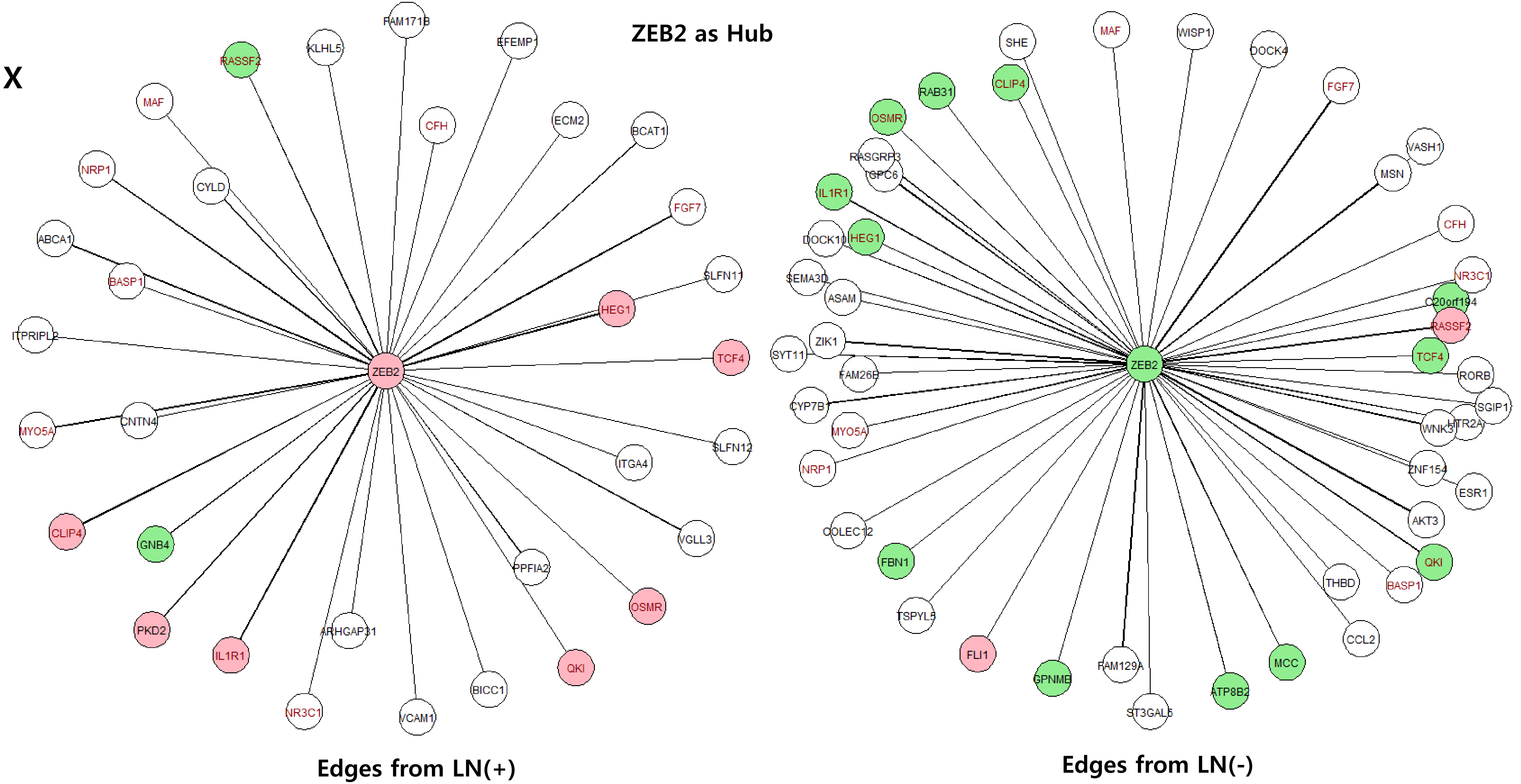

ZEB2 as Hub
ZEB2
FAM171B
KLHL5
RASSF2
EFEMP1
MAF
CFH
ECM2
BCAT1
NRP1
CYLD
FGF7
ABCA1
BASP1
SLFN11
HEG1
ITPRIPL2
TCF4
MYO5A
CNTN4
SLFN12
ITGA4
CLIP4
GNB4
VGLL3
PPFIA2
OSMR
PKD2
ARHGAP31
IL1R1
QKI
BICC1
NR3C1
VCAM1
SHE
WISP1
DOCK4
RAB31
FGF7
OSMR
RASGRP3
GPC6
VASH1
MSN
IL1R1
DOCK10
HEG1
CFH
SEMA3D
ASAM
NR3C1
C20orf194
RASSF2
ZIK1
SYT11
FAM26E
TCF4
RORB
CYP7B1
SGIP1
MYO5A
WNK3
HTR2A
NRP1
ZNF154
ESR1
COLEC12
AKT3
FBN1
QKI
THBD
BASP1
TSPYL5
CCL2
FLI1
FAM129A
MCC
GPNMB
ATP8B2
ST3GAL5
Edges from LN(+)
Edges from LN(-)

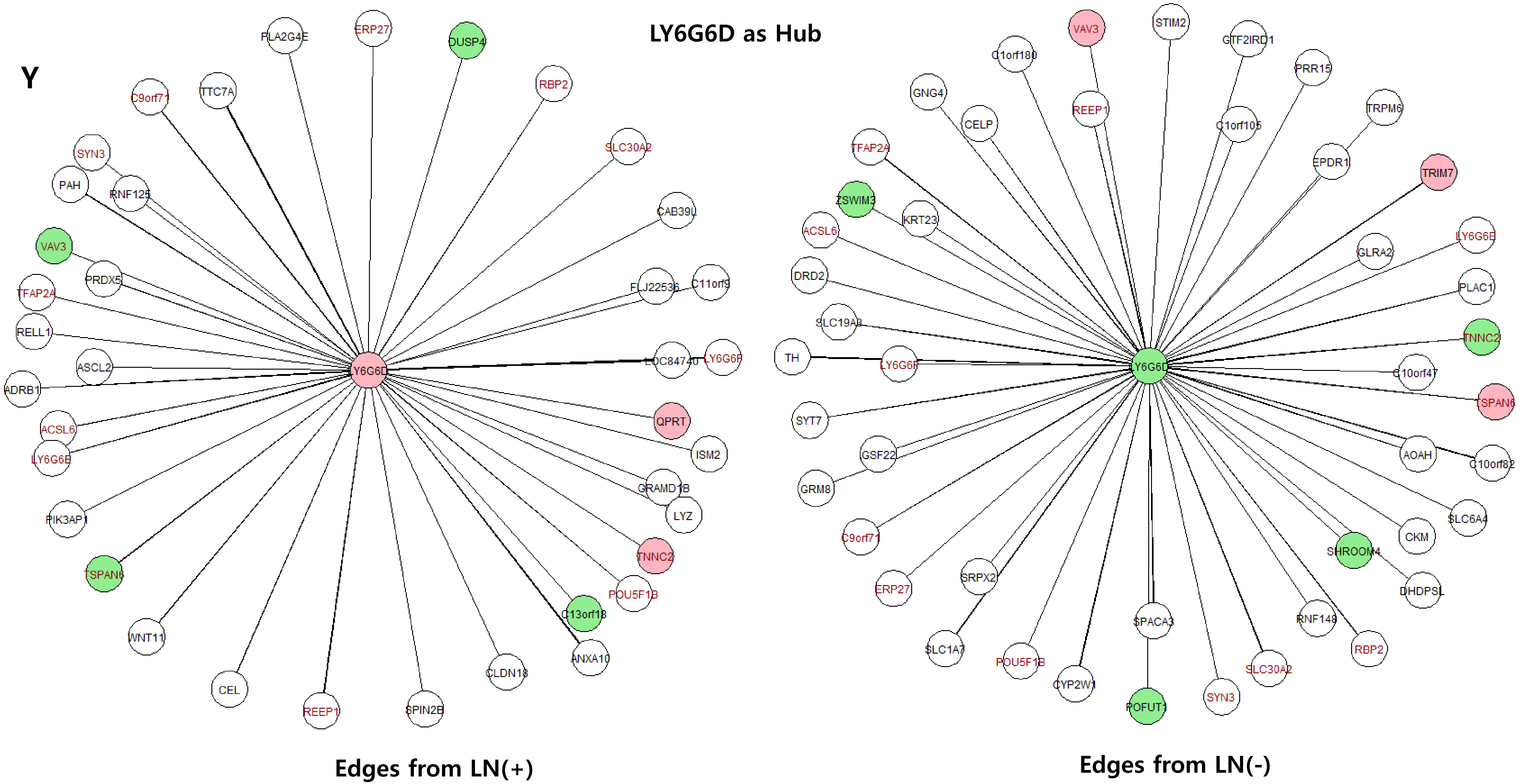

Y
LY6G6D as Hub
LY6G6D
PLA2G4E
ERP27
DUSP4
RBP2
SLC30A2
CAB39L
FLJ22536
C11orf9
LOC84740
LY6G6F
QPRT
ISM2
GRAMD1B
LYZ
TNNC2
POU5F1B
C13orf18
ANXA10
CLDN18
SPIN2B
REEP1
CEL
WNT11
TSPAN6
PIK3AP1
LY6G6E
ACSL6
ADRB1
ASCL2
RELL1
TFAP2A
PRDX5
VAV3
PAH
RNF125
SYN3
C9orf71
TTC7A
Edges from LN(+)
VAV3
STIM2
GTF2IRD1
C1orf180
PRR15
GNG4
REEP1
TRPM6
CELP
C1orf105
EPDR1
TFAP2A
TRIM7
ZSWIM3
KRT23
ACSL6
LY6G6E
GLRA2
DRD2
PLAC1
SLC19A3
TNNC2
TH
LY6G6F
C10orf47
TSPAN6
SYT7
AOAH
C10orf82
GSF22
GRM8
SLC6A4
CKM
C9orf71
SHROOM4
DHDPSL
ERP27
SRPX2
SPACA3
RNF149
SLC1A7
RBP2
POU5F1B
SLC30A2
CYP2W1
SYN3
POFUT1
Edges from LN(-)

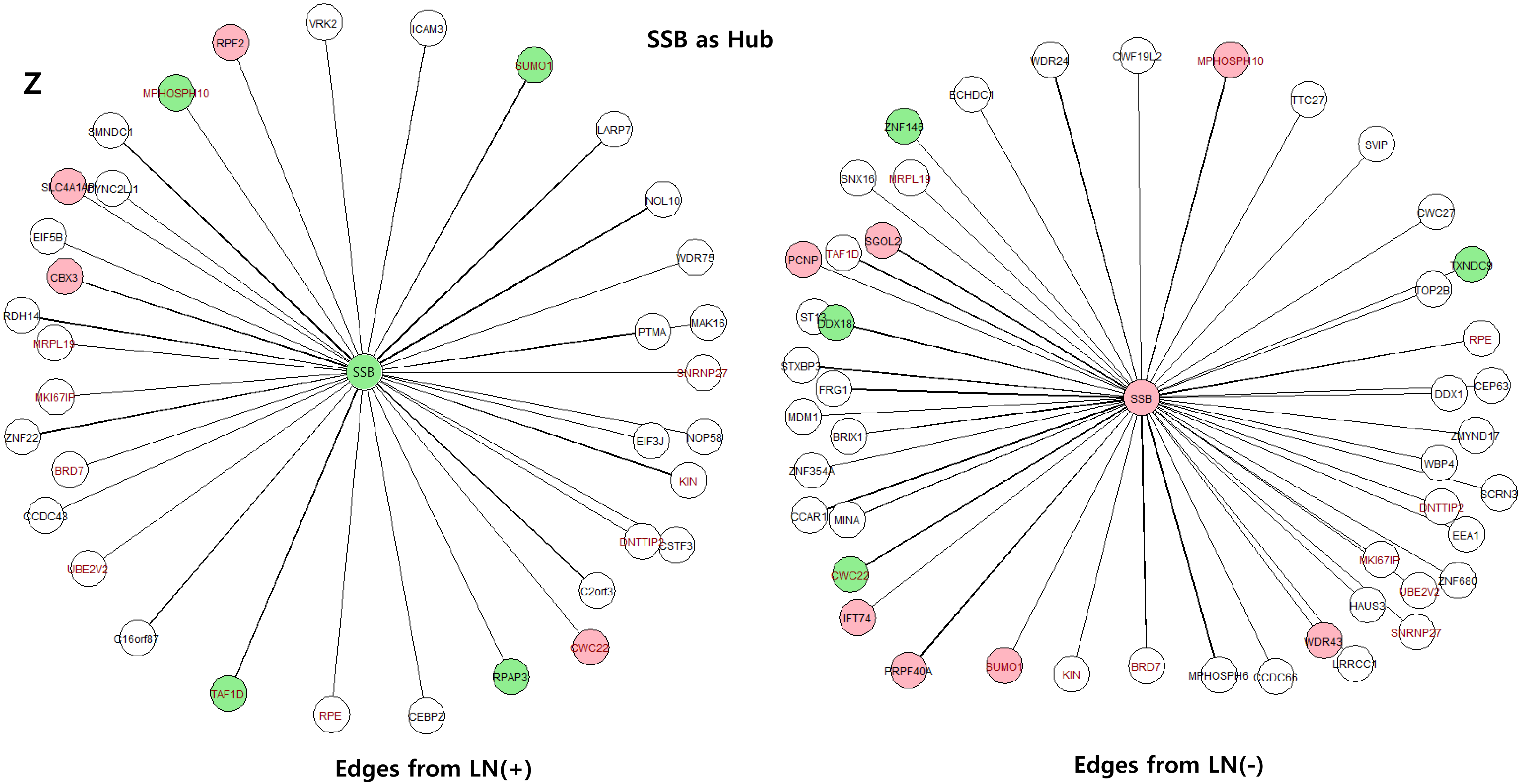
Z
SSB as Hub
VRK2
ICAM3
RPF2
SUMO1
MPHOSPH10
LARP7
SMNDC1
NOL10
SLC4A1AP
DYNC2LI1
EIF5B
WDR75
CBX3
RDH14
PTMA
MAK16
MRPL19
SSB
SNRNP27
MKI67IP
EIF3J
NOP58
ZNF22
BRD7
KIN
CCDC43
DNTTIP2
CSTF3
UBE2V2
C2orf3
C16orf87
CWC22
RPAP3
TAF1D
RPE
CEBPZ
Edges from LN(+)
WDR24
CWF19L2
MPHOSPH10
ECHDC1
TTC27
ZNF146
SVIP
SNX16
MRPL19
CWC27
SGOL2
TAF1D
PCNP
TXNDC9
TOP2B
ST13
DDX18
RPE
STXBP3
DDX1
CEP63
FRG1
SSB
MDM1
ZMYND17
BRIX1
WBP4
ZNF354A
SCRN3
DNTTIP2
CCAR1
MINA
EEA1
MKI67IP
ZNF680
CWC22
UBE2V2
HAUS3
IFT74
SNRNP27
WDR43
LRRCC1
PRPF40A
SUMO1
KIN
BRD7
MPHOSPH6
CCDC66
Edges from LN(-)

Supplementary Figure 4. Representative 22 of Hub of hub gene with its edge genes calculated by the degree of centrality analysis from the LN(+) and LN(-) groups (addition to Figure 2). Green fill: downregulated genes in the DEG analysis, Red fill: upregulated genes in the DEG analysis, Red font: common genes in both groups, Edge width: coefficient power

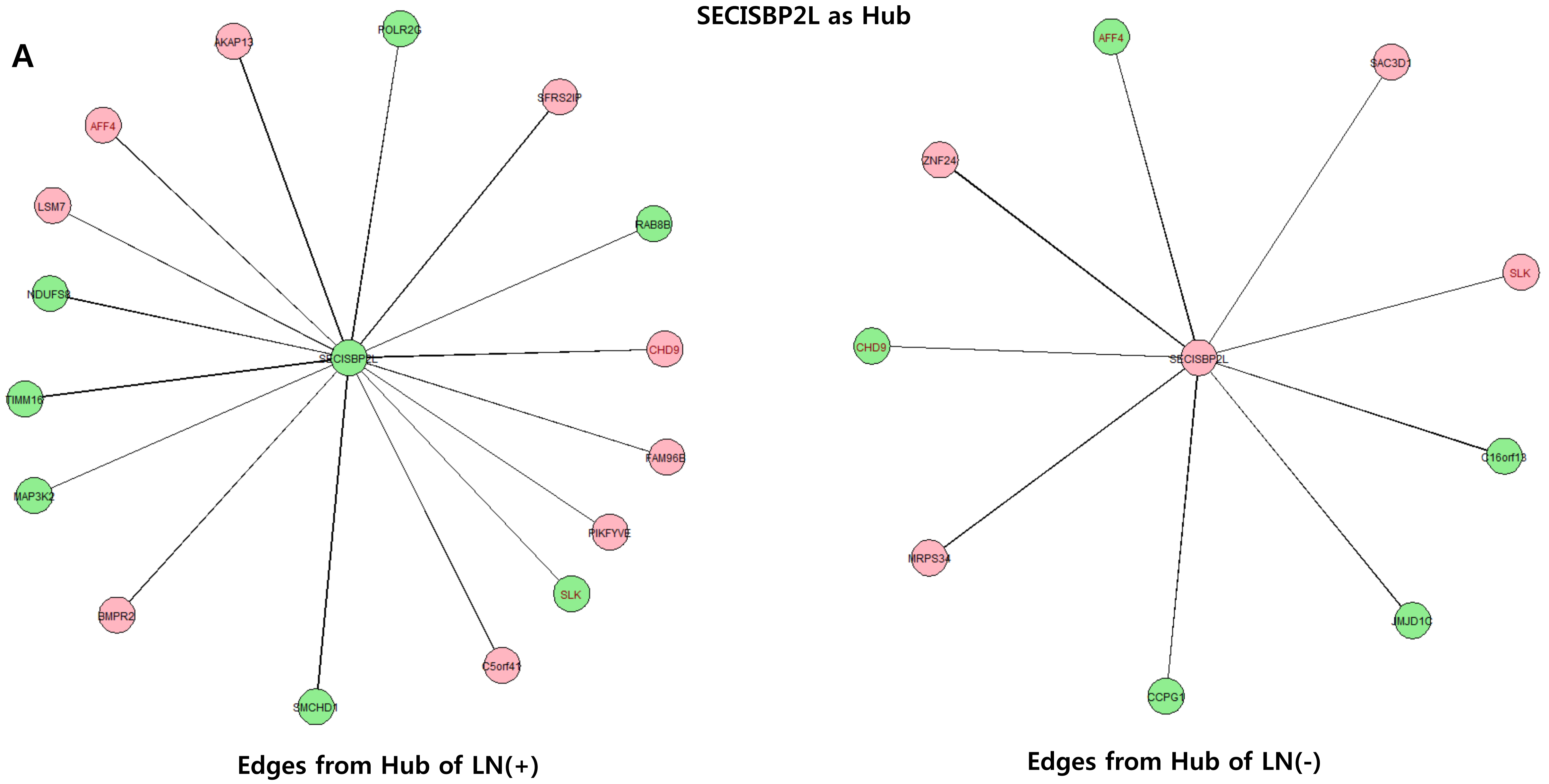

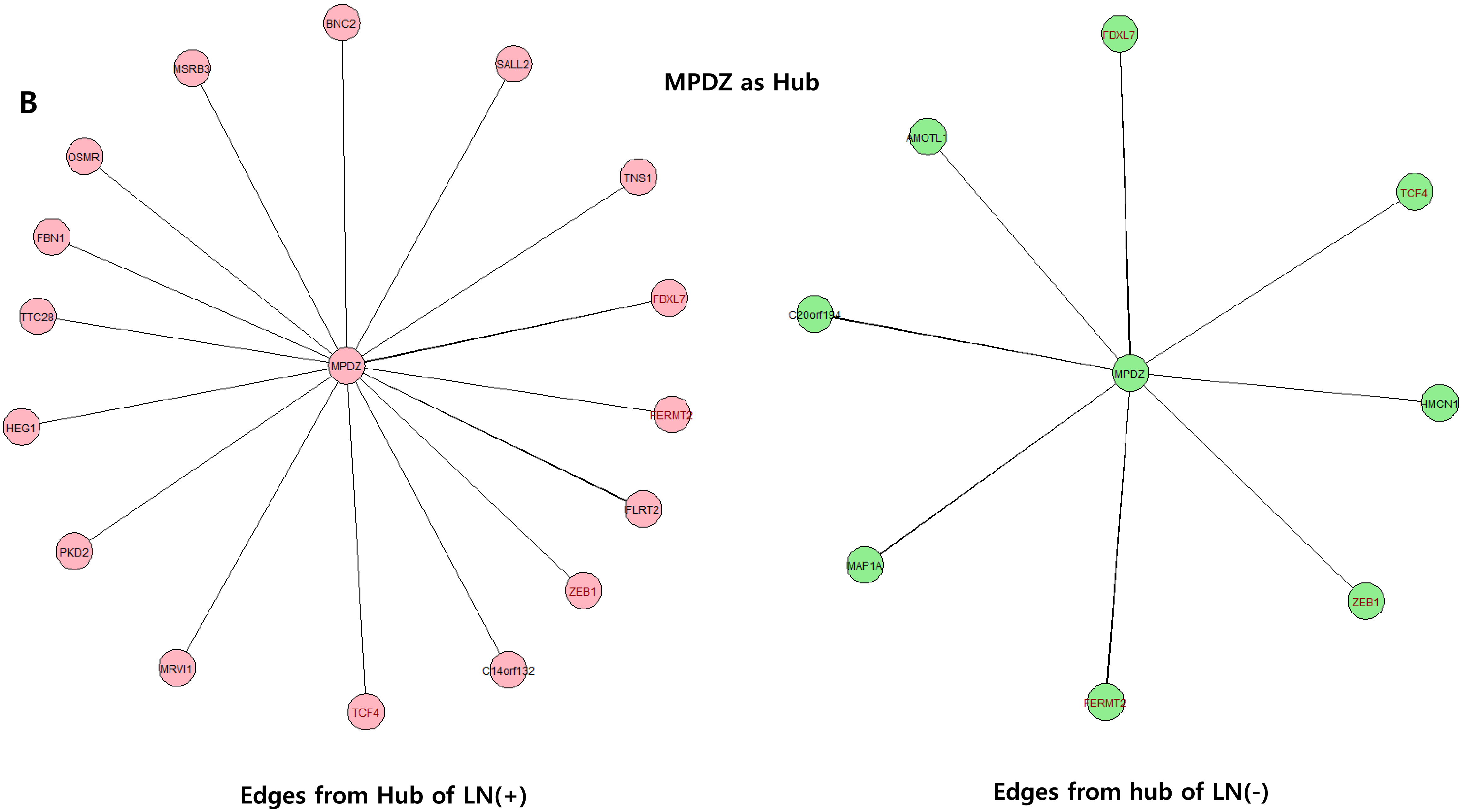
B
MPDZ as Hub
BNC2
MSRB3
SALL2
OSMR
TNS1
FBN1
FBXL7
TTC28
MPDZ
FERMT2
HEG1
FLRT2
PKD2
ZEB1
MRVI1
C14orf132
TCF4
Edges from Hub of LN(+)
FBXL7
AMOTL1
TCF4
C20orf194
MPDZ
HMCN1
MAP1A
ZEB1
FERMT2
Edges from hub of LN(-)

TCF4 as Hub

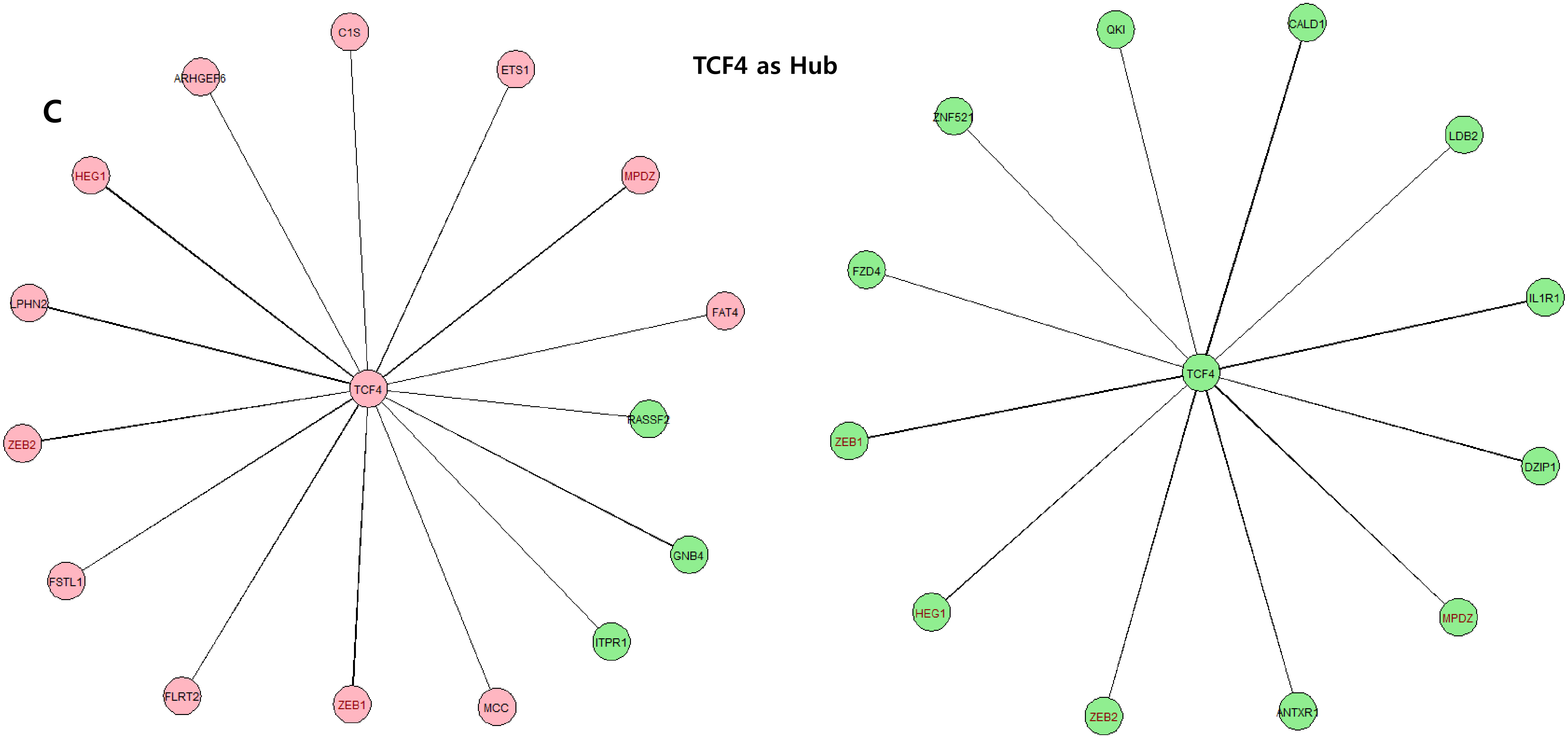

Edges from Hub of LN(+)

Edges from Hub of LN(-)

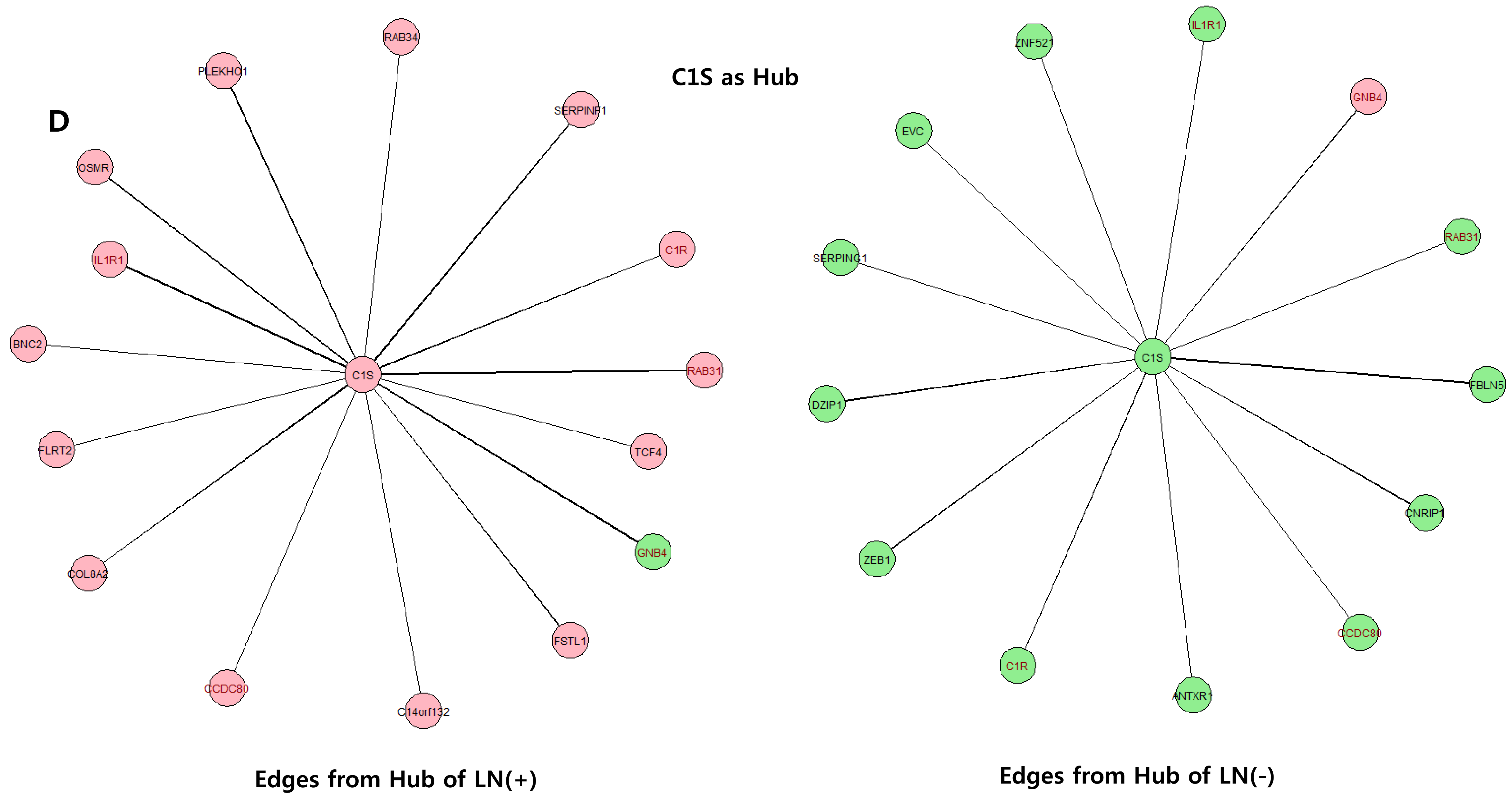

D
C1S as Hub
C1S
RAB34
PLEKHO1
SERPINF1
OSMR
C1R
IL1R1
BNC2
RAB31
FLRT2
TCF4
GNB4
COL8A2
FSTL1
CCDC80
C14orf132
Edges from Hub of LN(+)
ZNF521
IL1R1
GNB4
EVC
RAB31
SERPING1
FBLN5
DZIP1
CNRIP1
ZEB1
CCDC80
C1R
ANTXR1
Edges from Hub of LN(-)

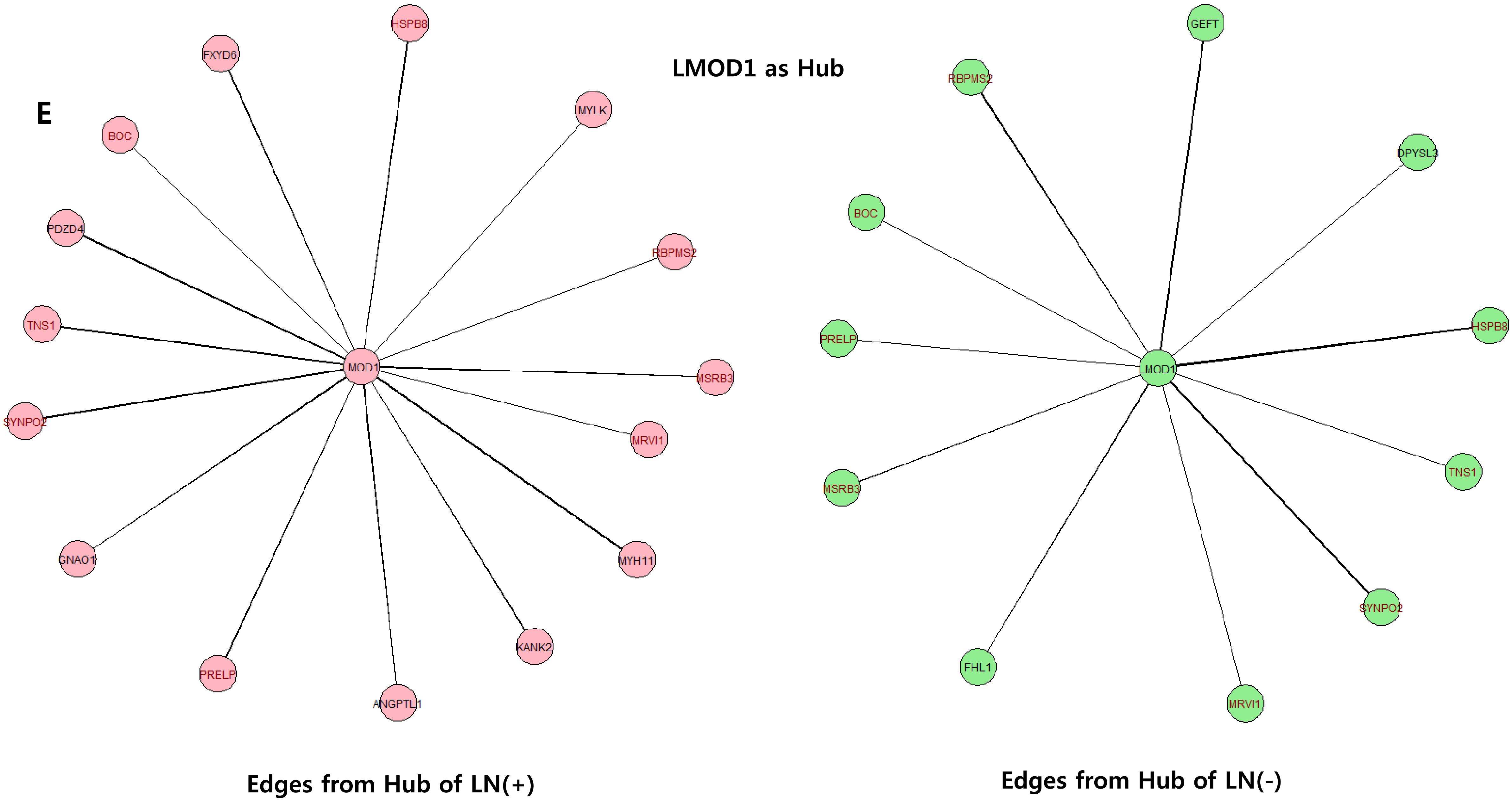

E
LMOD1 as Hub
HSPB8
FXYD6
MYLK
BOC
PDZD4
RBPMS2
TNS1
LMOD1
MSRB3
SYNPO2
MRVI1
GNAO1
MYH11
KANK2
PRELP
ANGPTL1
Edges from Hub of LN(+)
GEFT
RBPMS2
DPYSL3
BOC
HSPB8
PRELP
LMOD1
MSRB3
TNS1
SYNPO2
FHL1
MRVI1
Edges from Hub of LN(-)

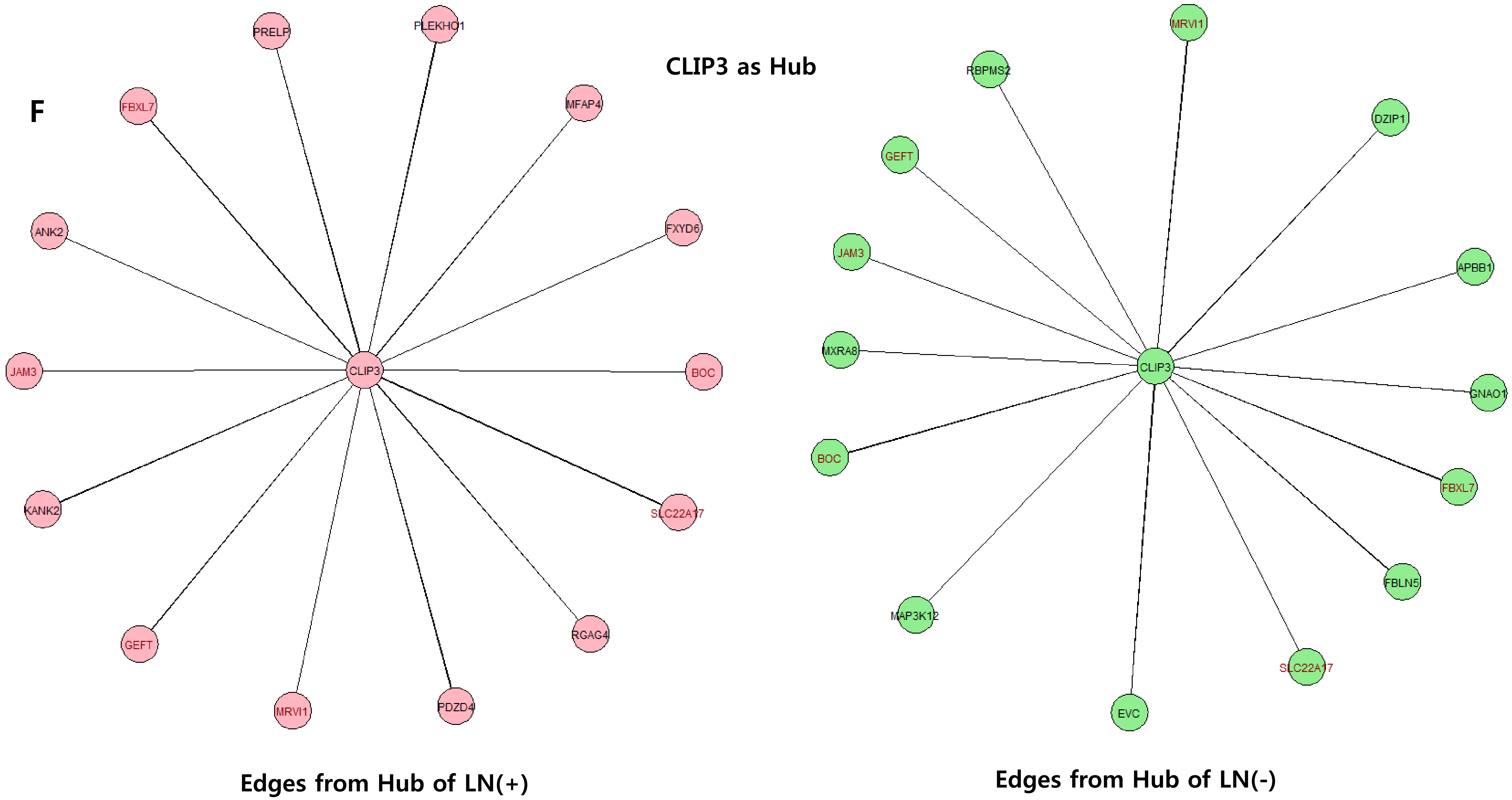

F
CLIP3 as Hub
PRELP
PLEKHO1
FBXL7
MFAP4
ANK2
FXYD6
JAM3
CLIP3
BOC
KANK2
SLC22A17
GEFT
RGAG4
MRVI1
PDZD4
Edges from Hub of LN(+)
MRVI1
RBPMS2
DZIP1
GEFT
APBB1
JAM3
MXRA8
CLIP3
GNAO1
BOC
FBXL7
FBLN5
MAP3K12
SLC22A17
EVC
Edges from Hub of LN(-)

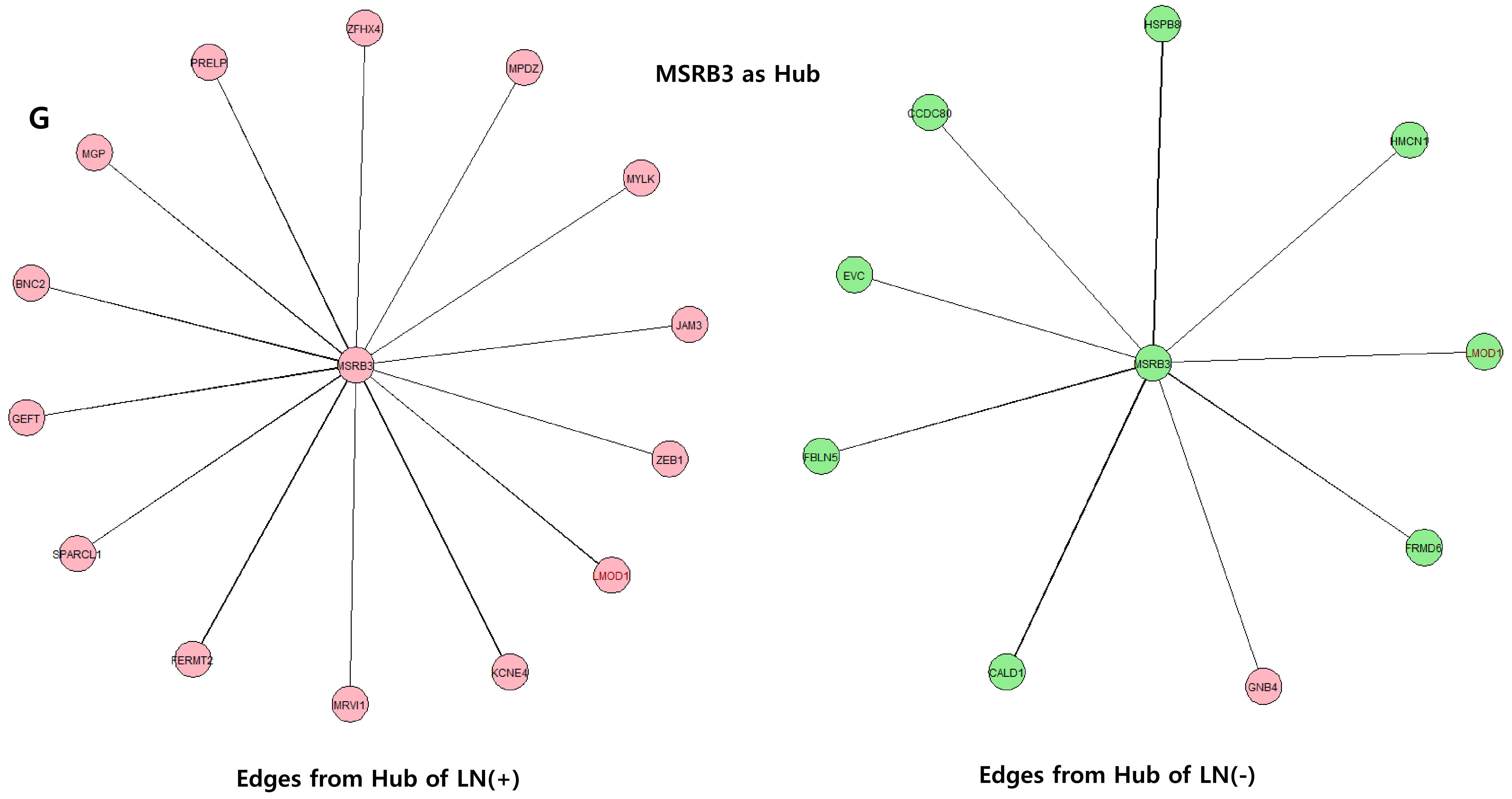

G
MSRB3 as Hub
ZFHX4
PRELP
MPDZ
MGP
MYLK
BNC2
JAM3
MSRB3
GEFT
ZEB1
SPARCL1
LMOD1
FERMT2
KCNE4
MRVI1
Edges from Hub of LN(+)
HSPB8
CCDC80
HMCN1
EVC
LMOD1
MSRB3
FBLN5
FRMD6
CALD1
GNB4
Edges from Hub of LN(-)

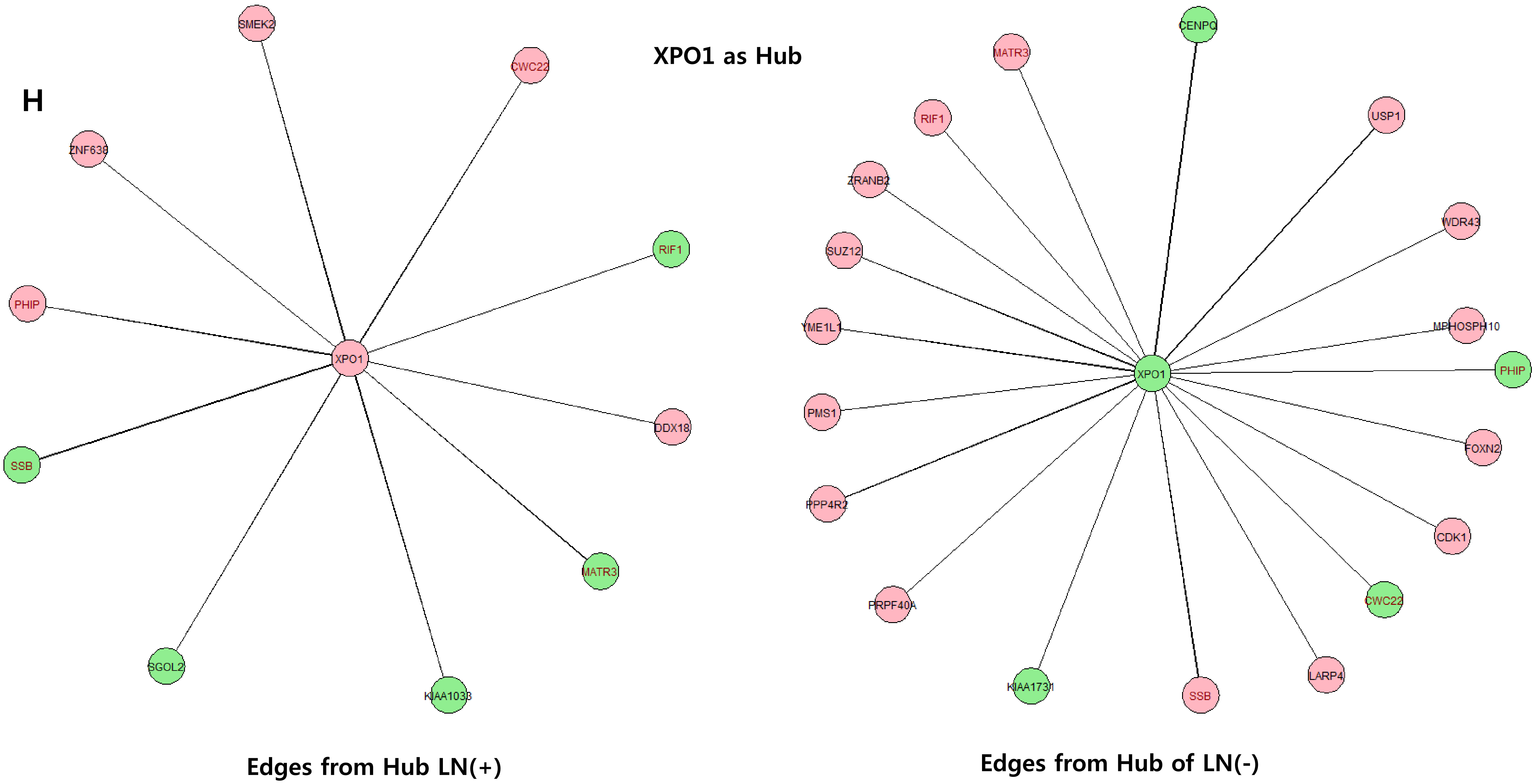
H
XPO1 as Hub
SMEK2
CWC22
ZNF638
RIF1
PHIP
XPO1
DDX18
SSB
MATR3
SGOL2
KIAA1033
Edges from Hub LN(+)
CENPC
MATR3
RIF1
USP1
ZRANB2
WDR43
SUZ12
YME1L1
MPHOSPH10
XPO1
PHIP
PMS1
FOXN2
PPP4R2
CDK1
PRPF40A
CWC22
KIAA1731
SSB
LARP4
Edges from Hub of LN(-)

I

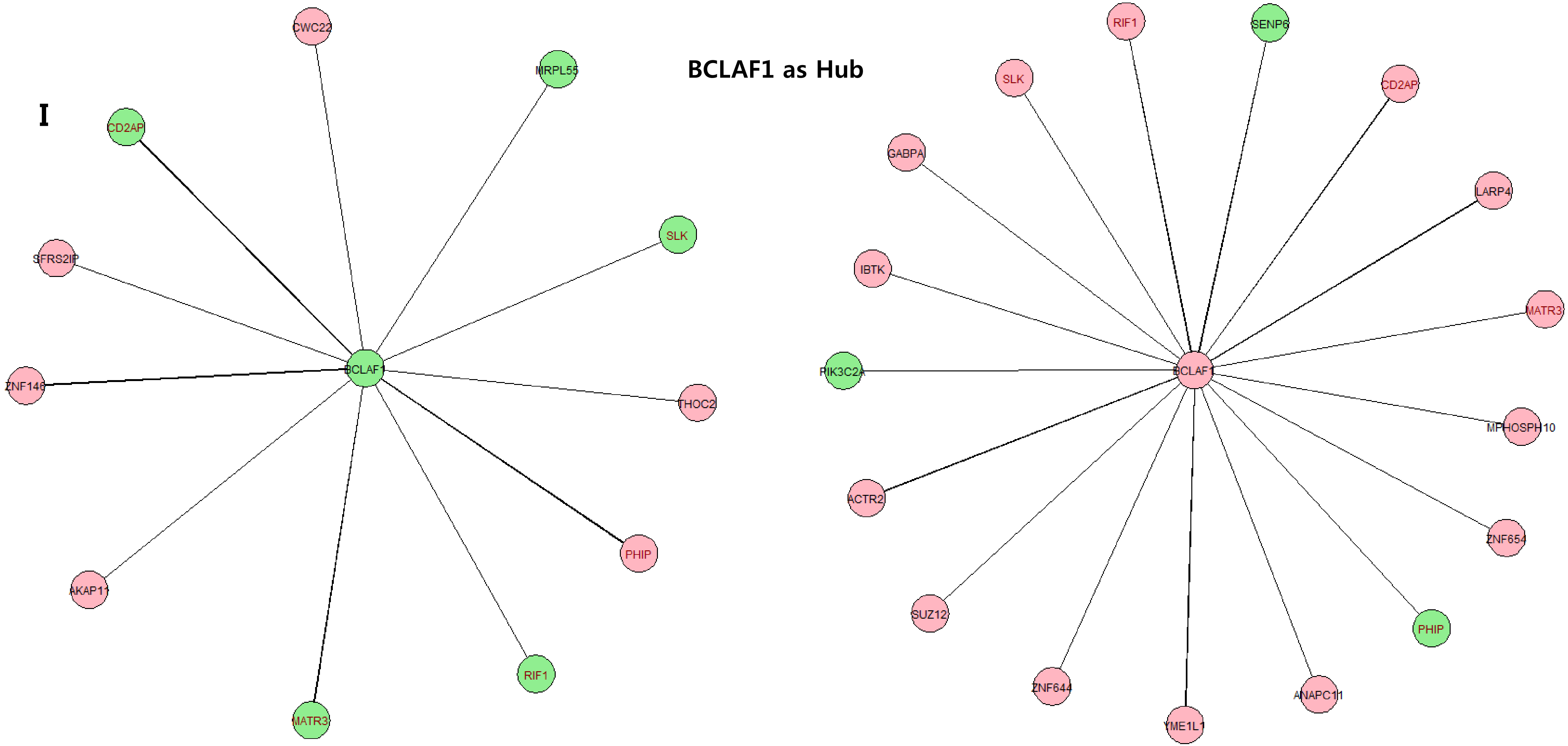

Edges from Hub of LN(+)

Edges from Hub of LN(-)

TNS1 as Hub

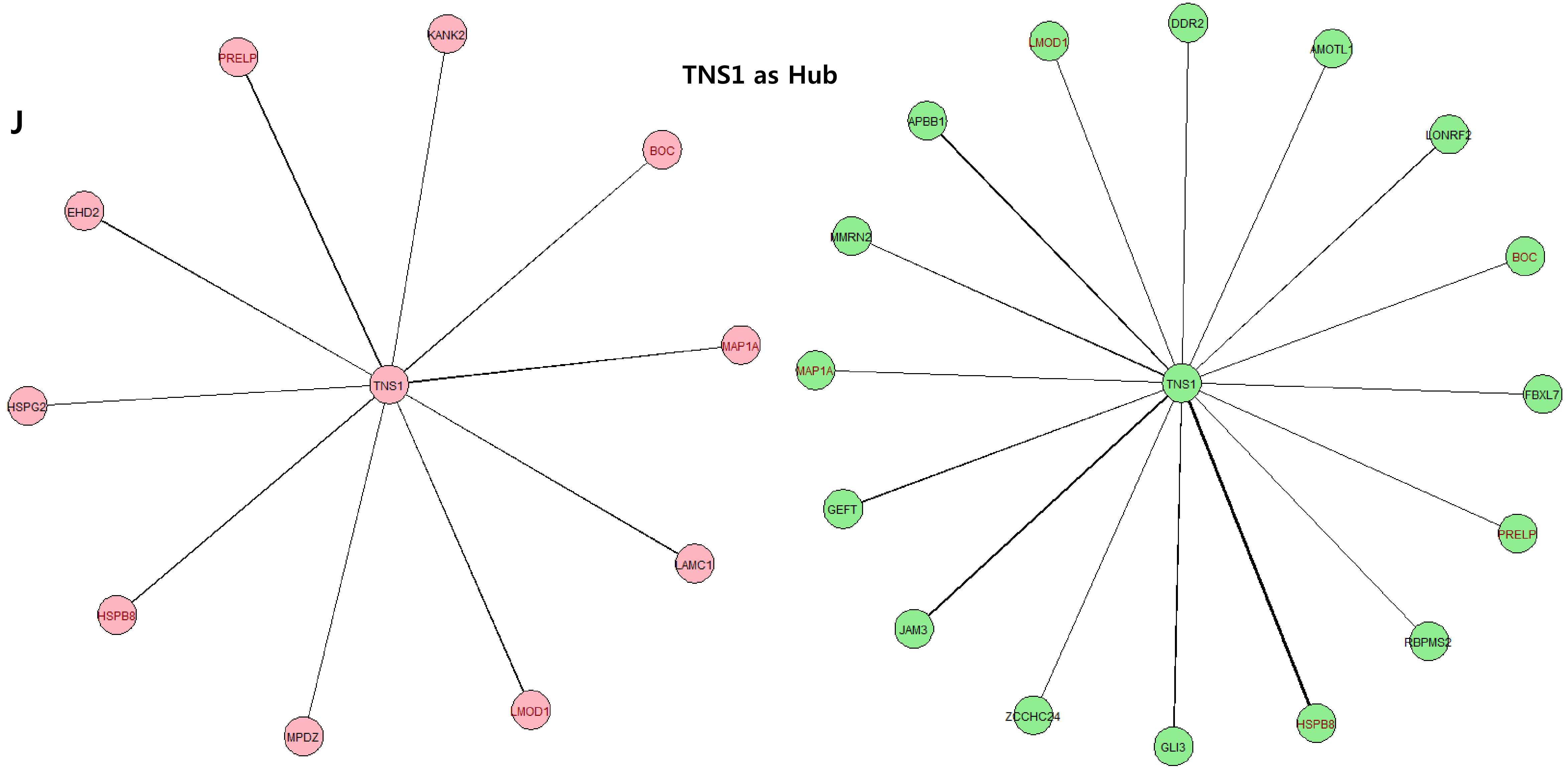

Edges from Hub of LN(+)

Edges from Hub of LN(-)

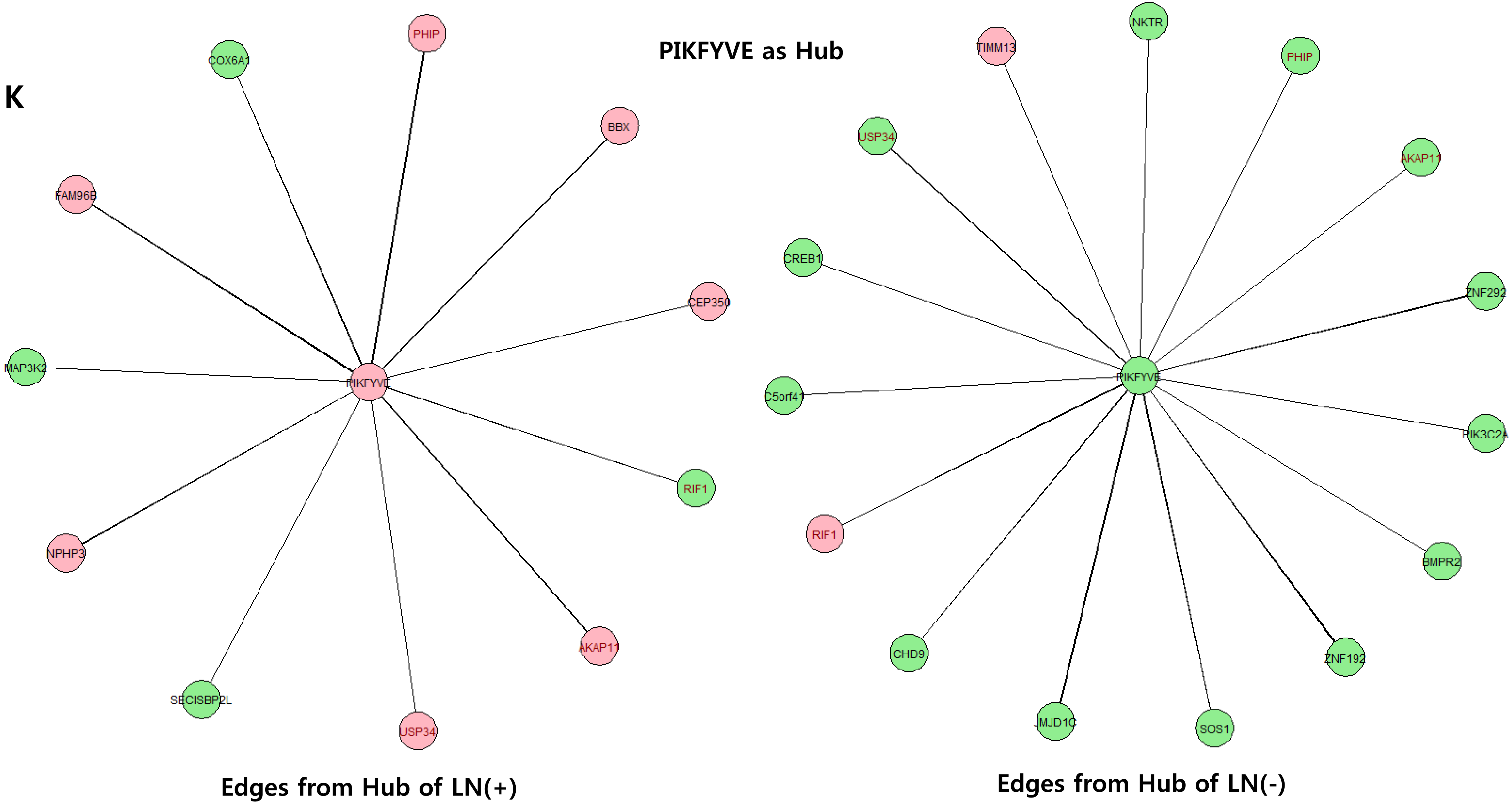
K
PIKFYVE as Hub
PHIP
COX6A1
BBX
FAM96B
CEP350
MAP3K2
PIKFYVE
RIF1
NPHP3
AKAP11
SECISBP2L
USP34
Edges from Hub of LN(+)
NKTR
TIMM13
PHIP
USP34
AKAP11
CREB1
ZNF292
C5orf41
PIKFYVE
PIK3C2A
RIF1
BMPR2
CHD9
ZNF192
JMJD1C
SOS1
Edges from Hub of LN(-)

L

CWC22 as Hub

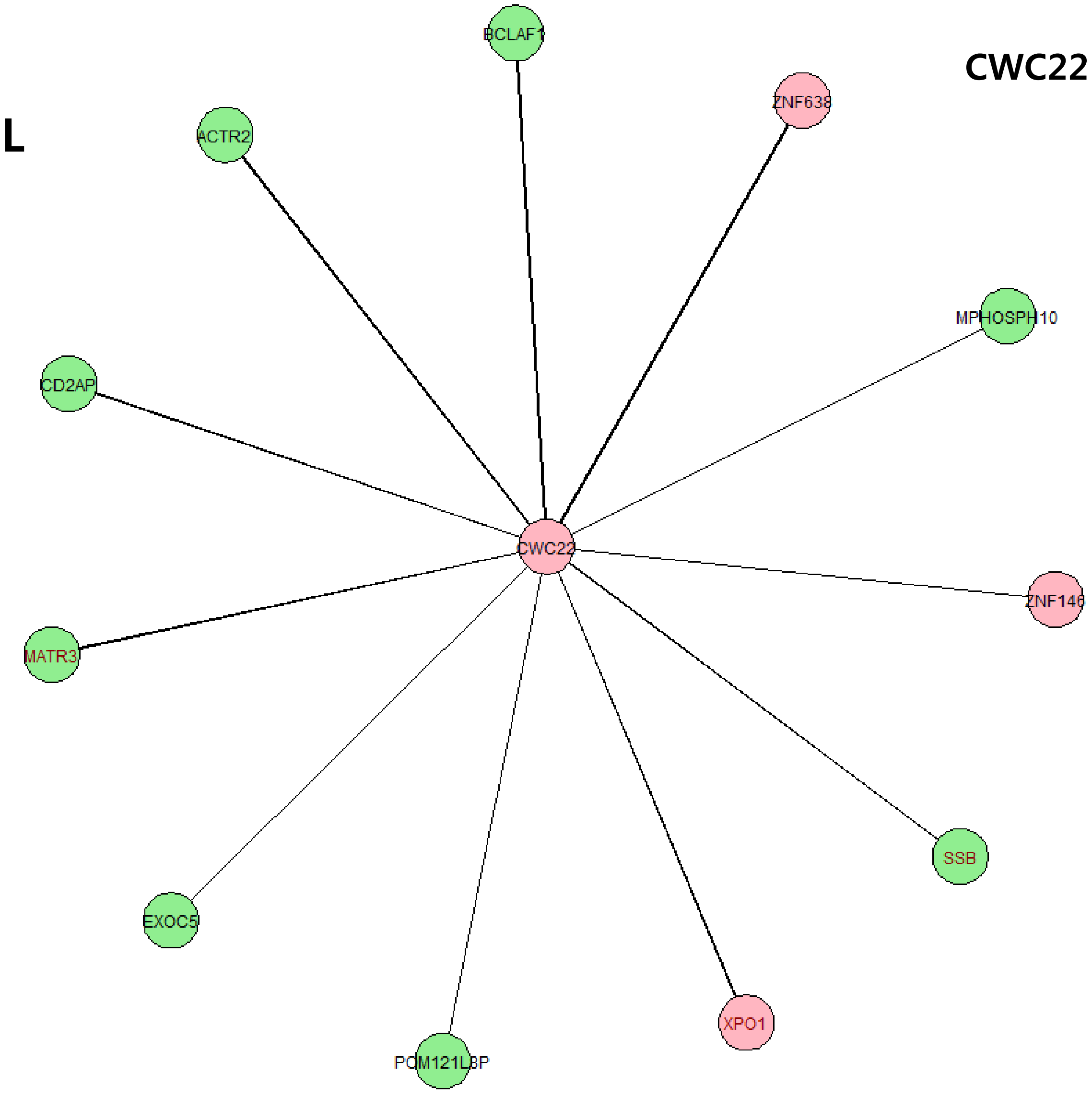

Edges from Hub of LN(+)

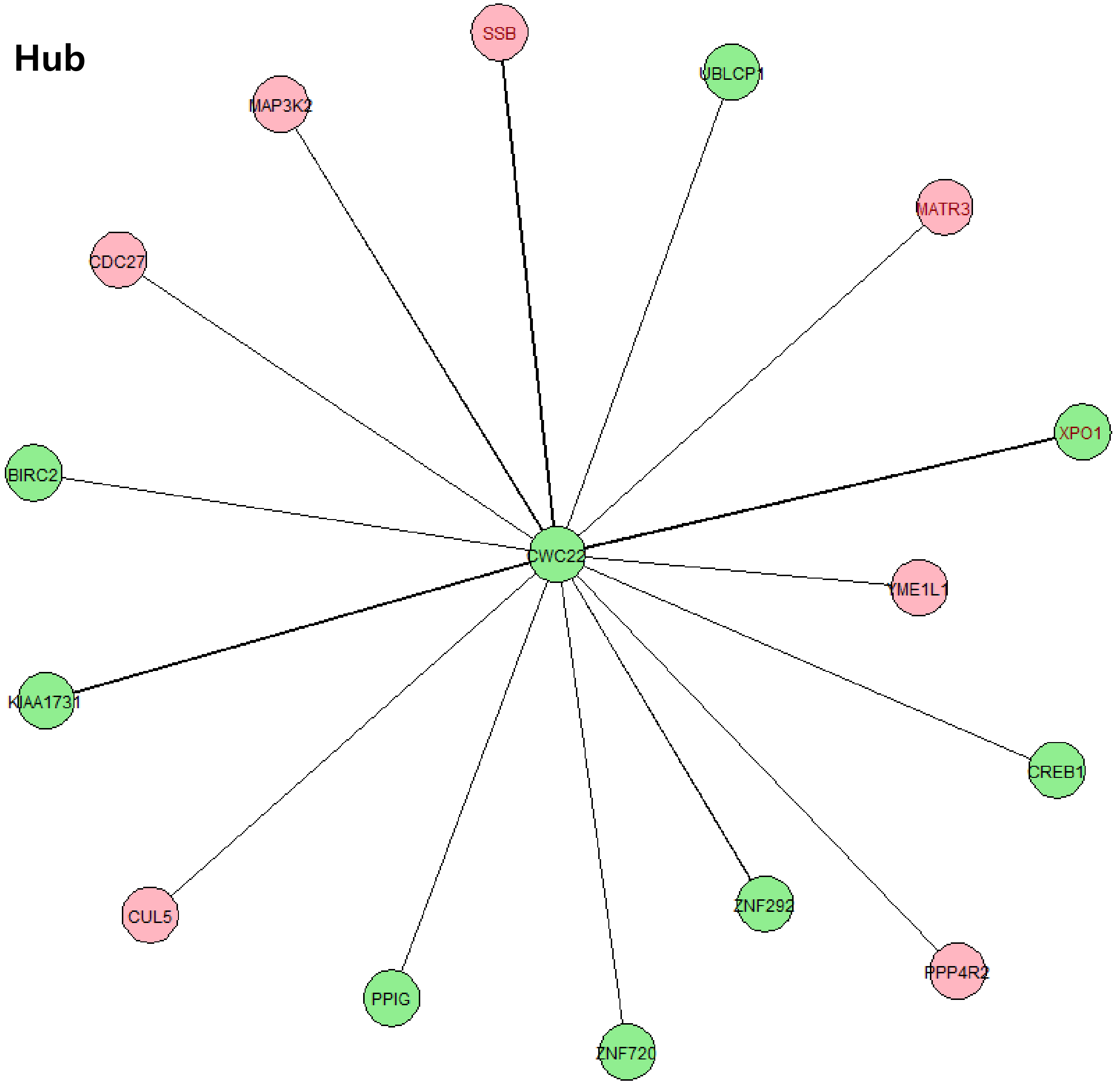

Edges from Hub of LN(-)

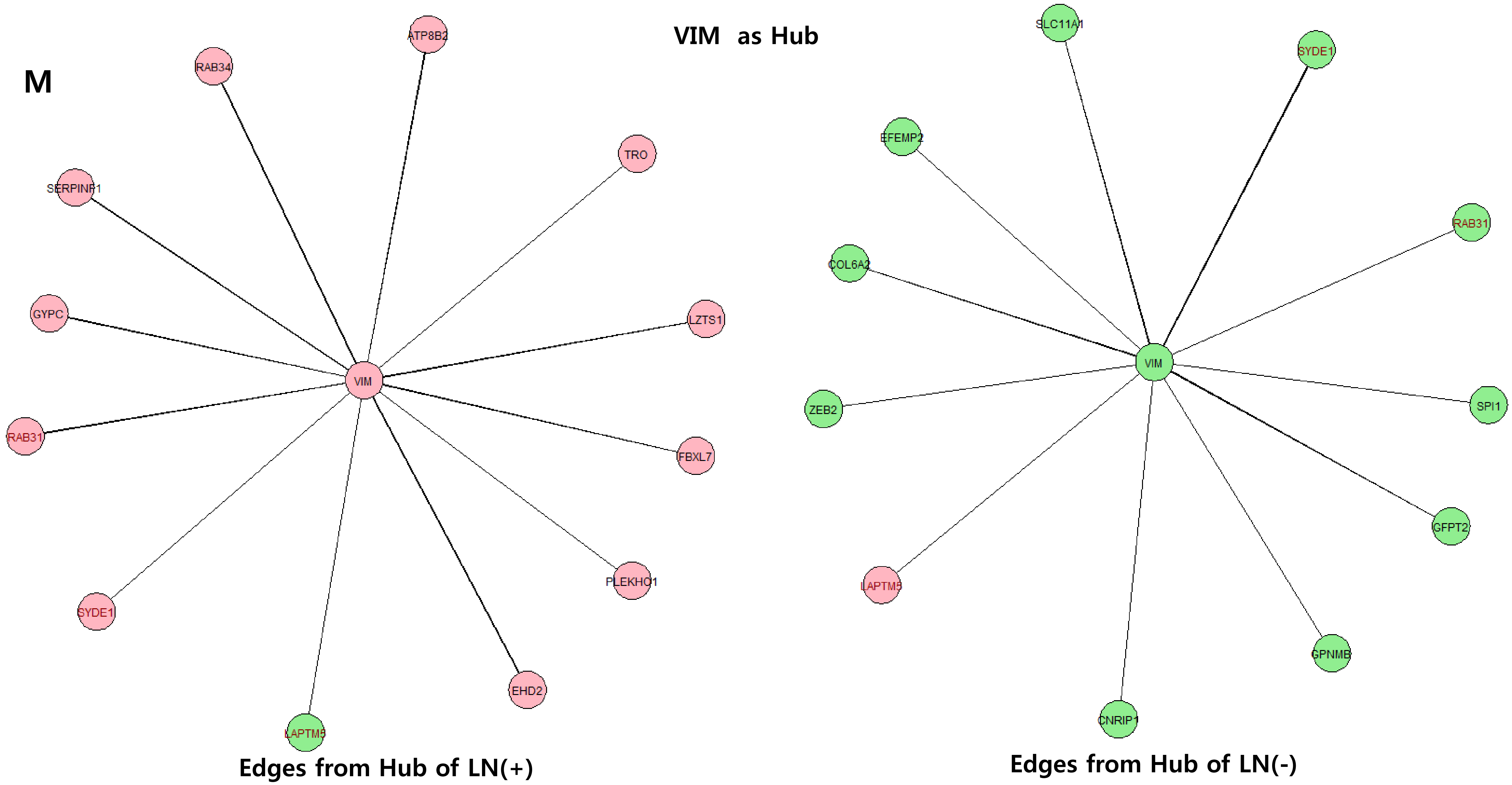
M
VIM as Hub
ATP8B2
RAB34
TRO
SERPINF1
GYPC
LZTS1
VIM
RAB31
FBXL7
PLEKHO1
SYDE1
EHD2
LAPTM5
Edges from Hub of LN(+)
SLC11A1
SYDE1
EFEMP2
RAB31
COL6A2
VIM
ZEB2
SPI1
GFPT2
LAPTM5
GPNMB
CNRIP1
Edges from Hub of LN(-)

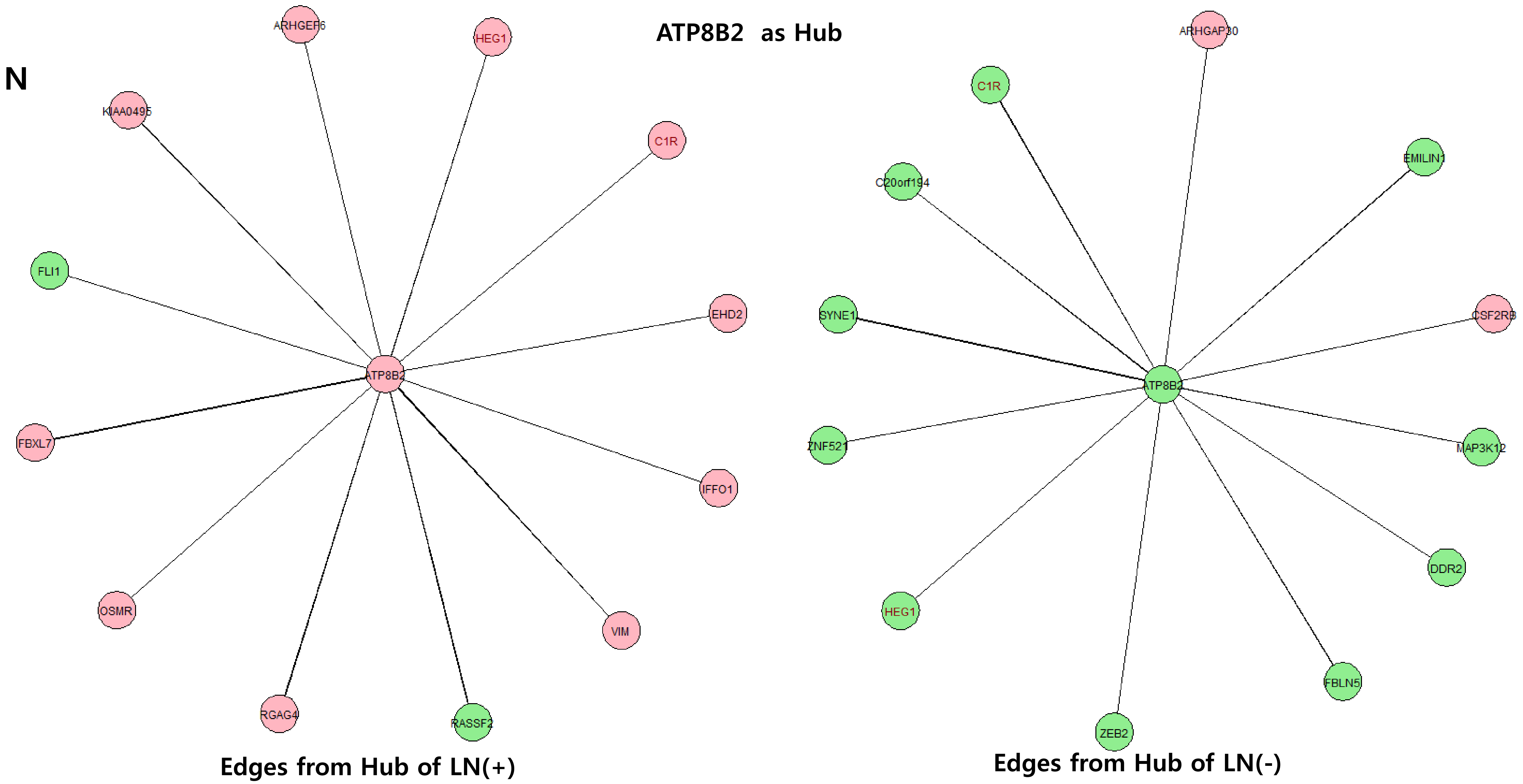
ATP8B2 as Hub
N
ARHGEF6
HEG1
KIAA0495
C1R
FLI1
EHD2
ATP8B2
FBXL7
IFFO1
OSMR
VIM
RGAG4
RASSF2
Edges from Hub of LN(+)
ARHGAP30
C1R
EMILIN1
C20orf194
SYNE1
CSF2RB
ATP8B2
ZNF521
MAP3K12
DDR2
HEG1
FBLN5
ZEB2
Edges from Hub of LN(-)

O

VPS26A as Hub

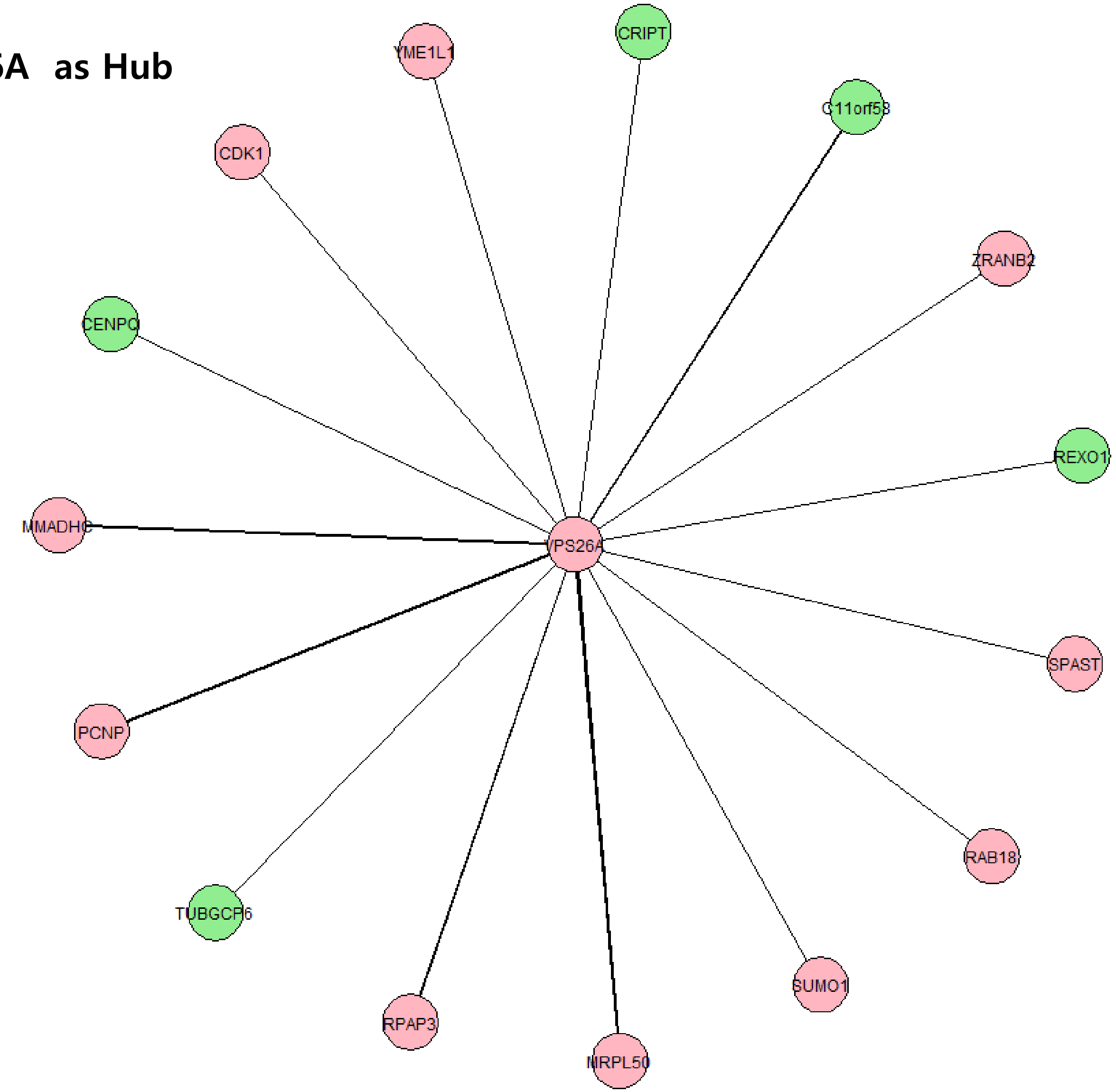

* Edges from Hub of LN(+)

Edges from Hub of LN(-)

*: VPS26A was not identified as hub of hub gene in LN(+) group

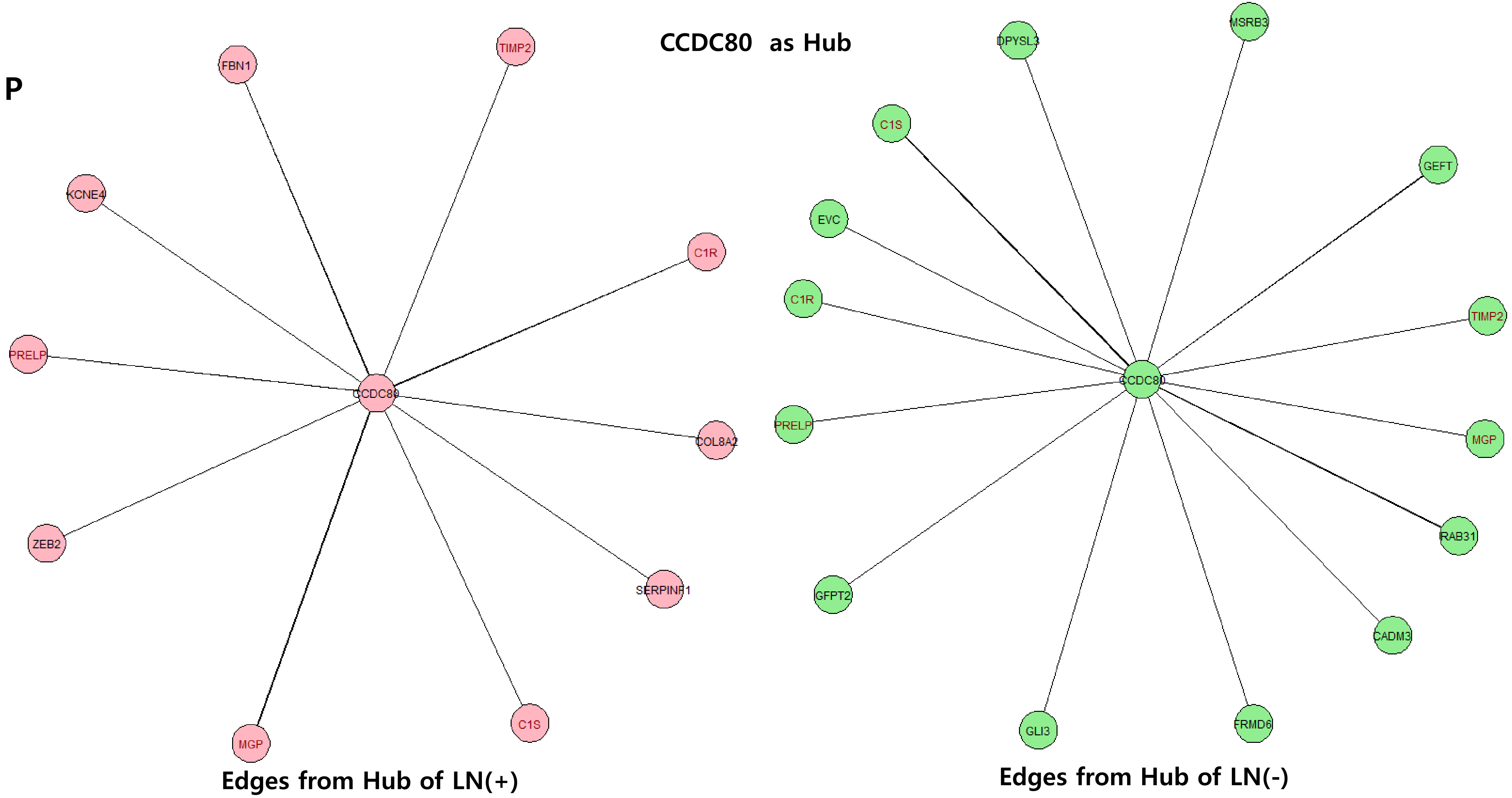

P
CCDC80 as Hub
CCDC80
FBN1
TIMP2
KCNE4
C1R
PRELP
COL8A2
ZEB2
SERPINF1
MGP
C1S
Edges from Hub of LN(+)
CCDC80
DPYSL3
MSRB3
C1S
GEFT
EVC
C1R
TIMP2
PRELP
MGP
RAB31
GFPT2
CADM3
GLI3
FRMD6
Edges from Hub of LN(-)

MATR3 as Hub

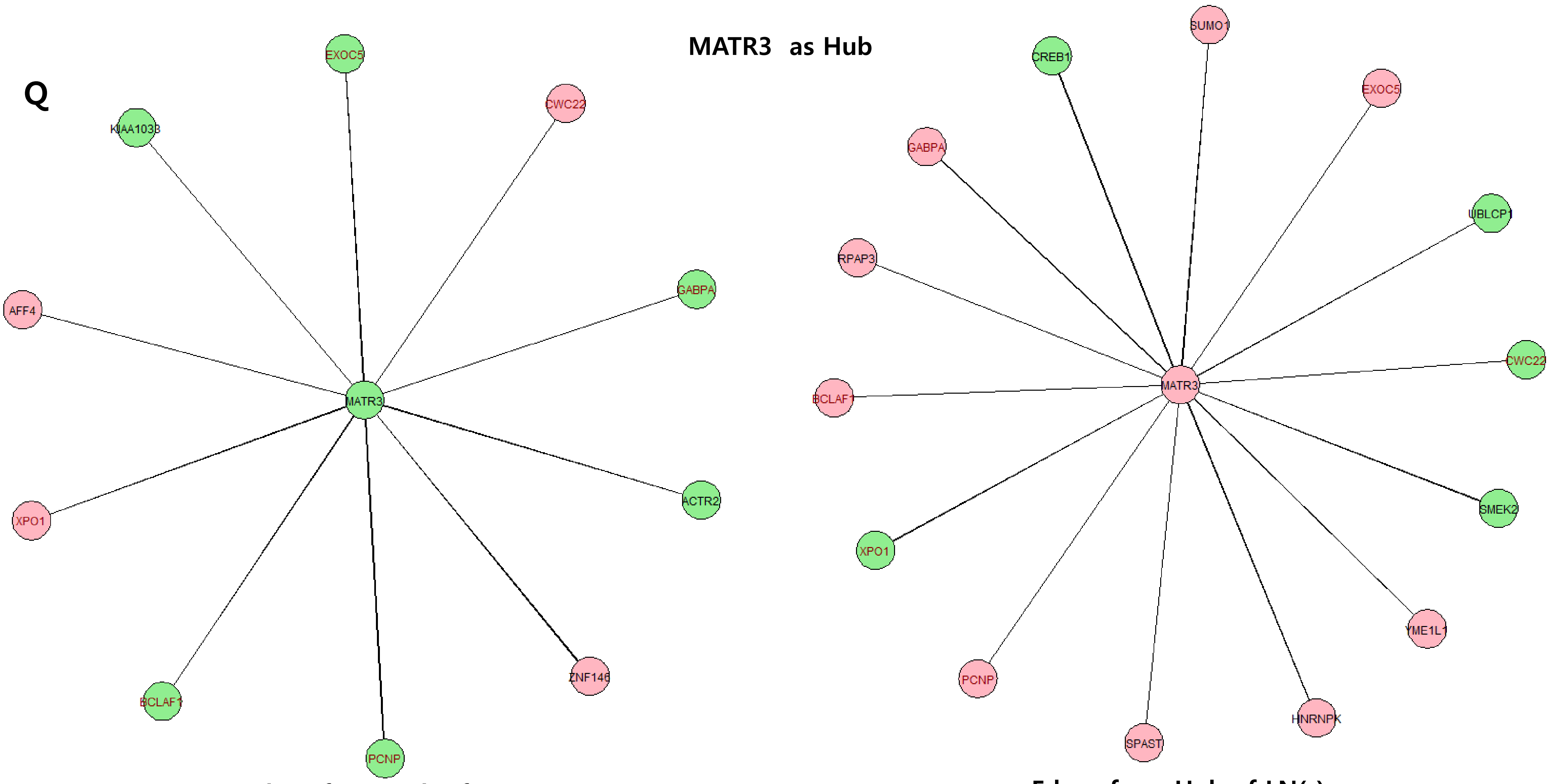

Edges from Hub of LN(+)

Edges from Hub of LN(-)

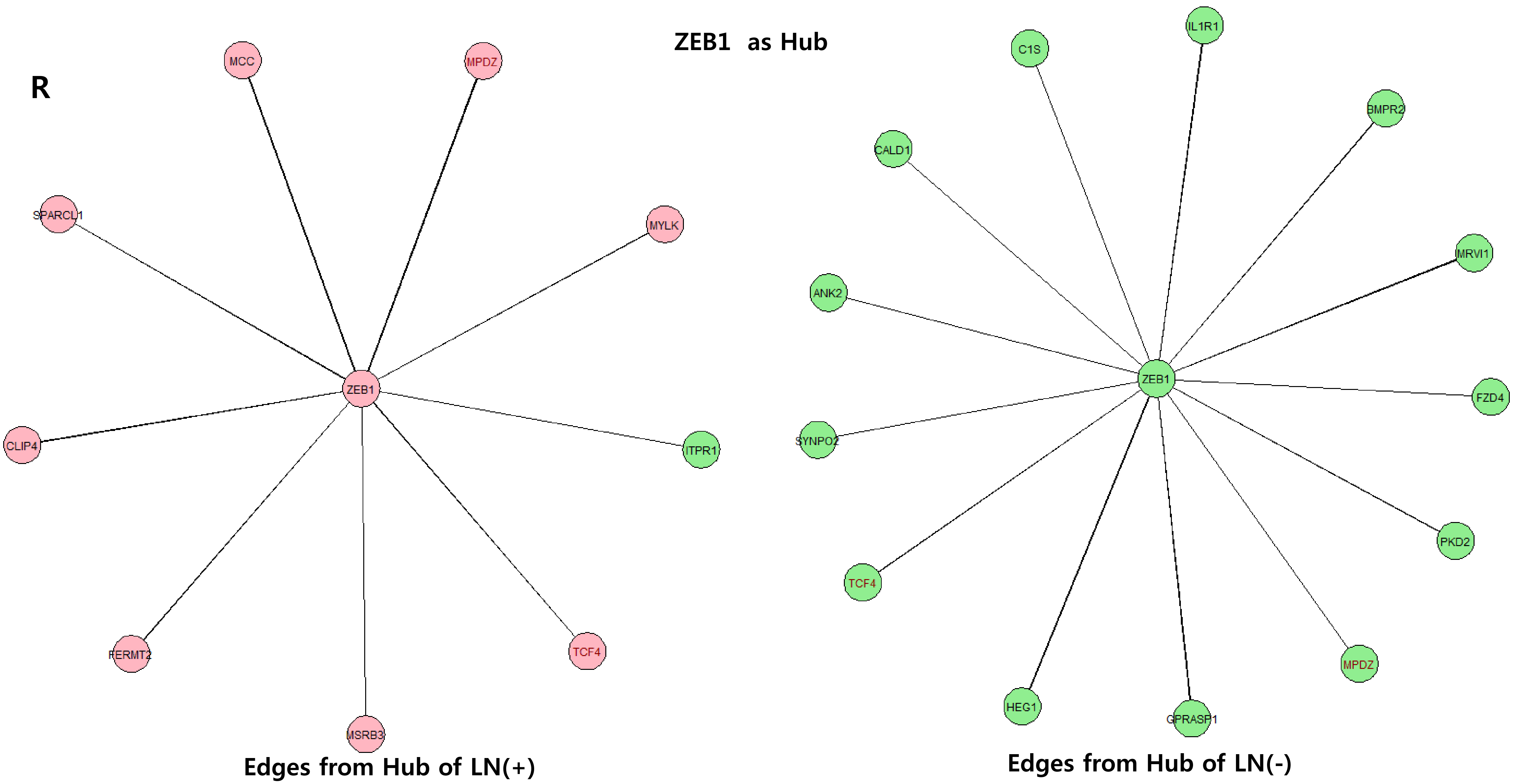

ZEB1 as Hub
R
MCC
MPDZ
SPARCL1
MYLK
ZEB1
CLIP4
ITPR1
FERMT2
TCF4
MSRB3
Edges from Hub of LN(+)
IL1R1
C1S
BMPR2
CALD1
MRVI1
ANK2
ZEB1
FZD4
SYNPO2
PKD2
TCF4
MPDZ
HEG1
GPRASP1
Edges from Hub of LN(-)

S

AFF4 as Hub

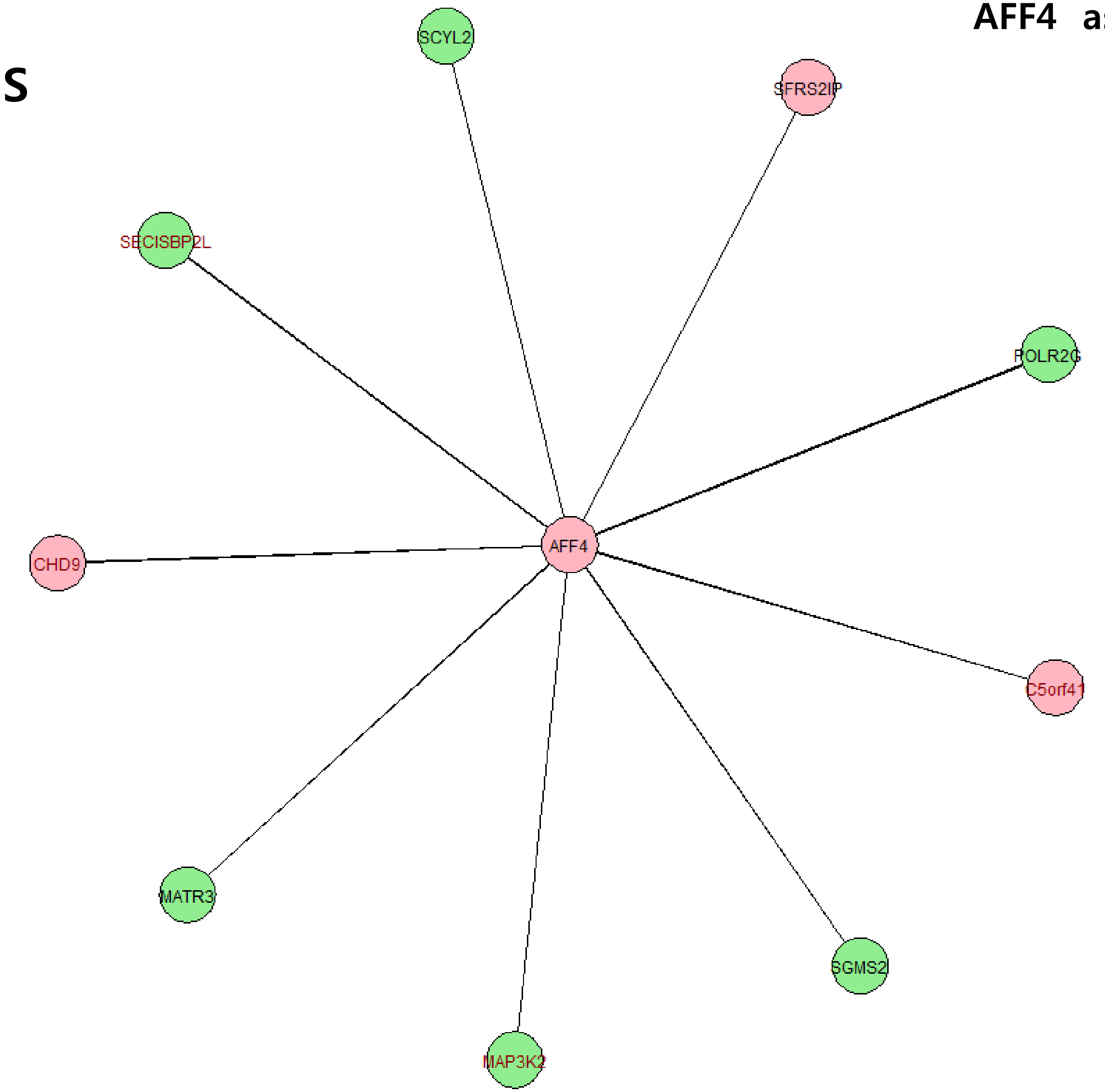

Edges from Hub of LN(+)

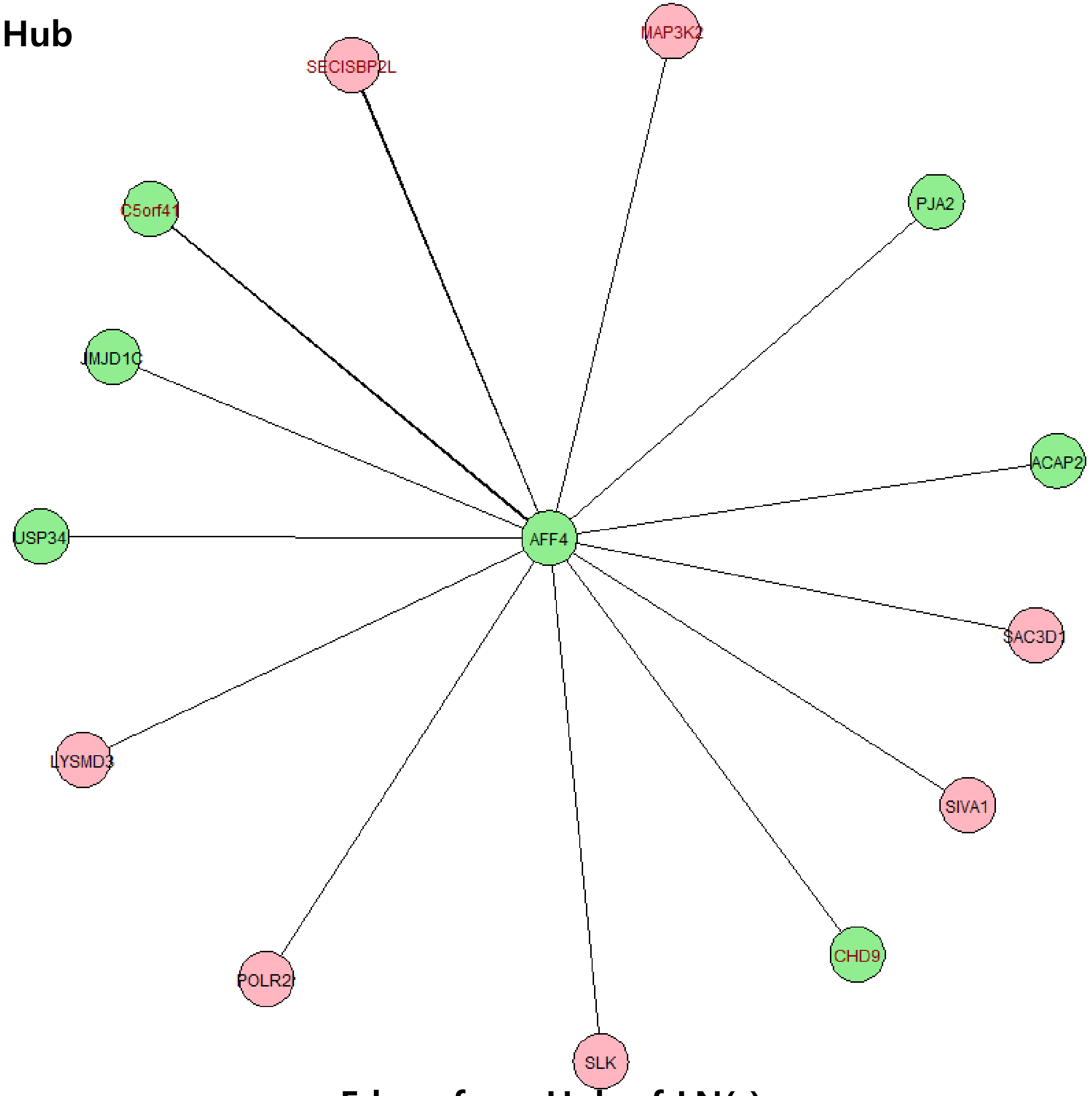

Edges from Hub of LN(-)

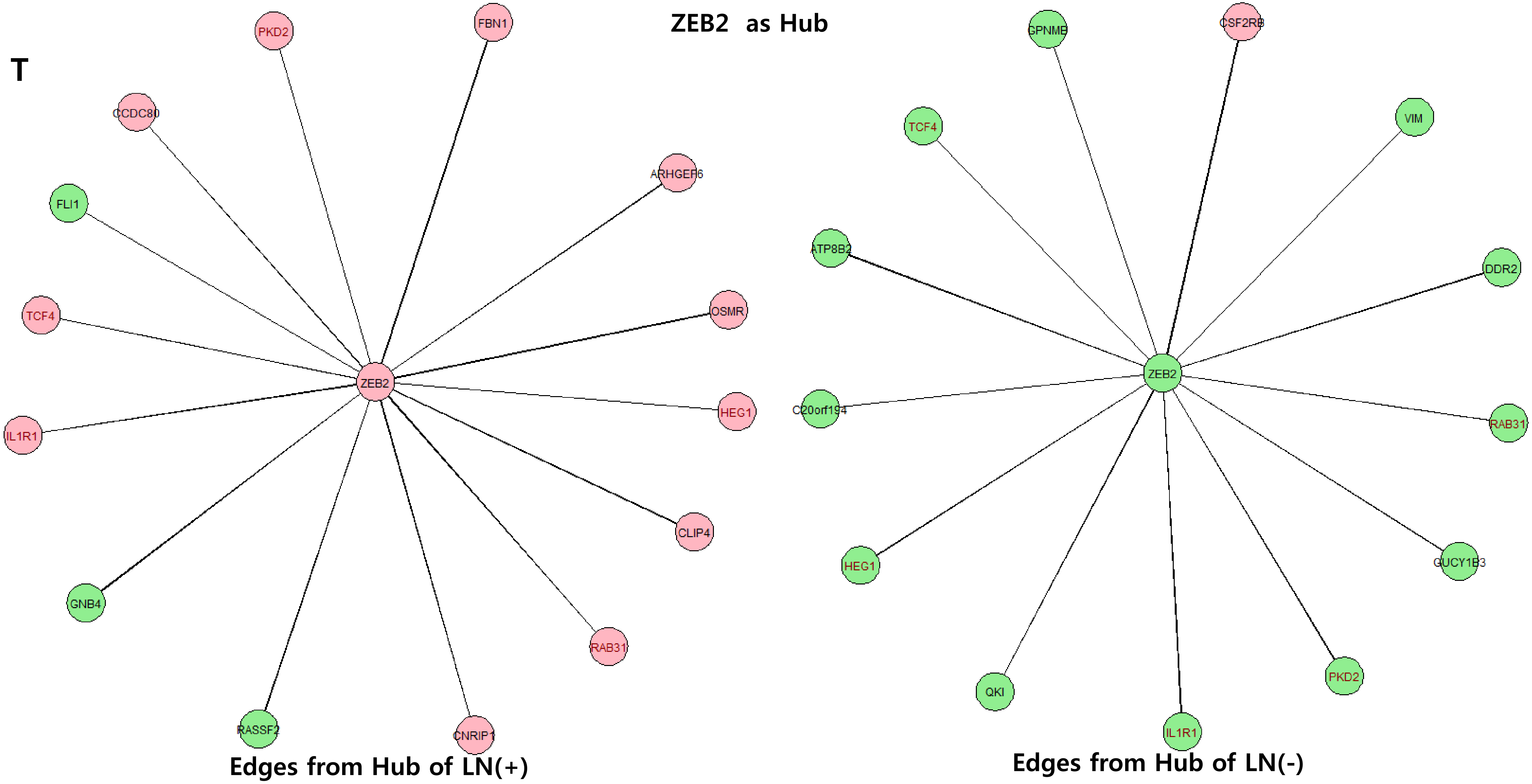
ZEB2 as Hub
T
PKD2
FBN1
CCDC80
ARHGEF6
FLI1
OSMR
TCF4
ZEB2
HEG1
IL1R1
CLIP4
GNB4
RAB31
RASSF2
CNRIP1
Edges from Hub of LN(+)
GPNMB
CSF2RB
TCF4
VIM
ATP8B2
DDR2
ZEB2
C20orf194
RAB31
HEG1
GUCY1B3
QKI
PKD2
IL1R1
Edges from Hub of LN(-)

LY6G6D as Hub

U

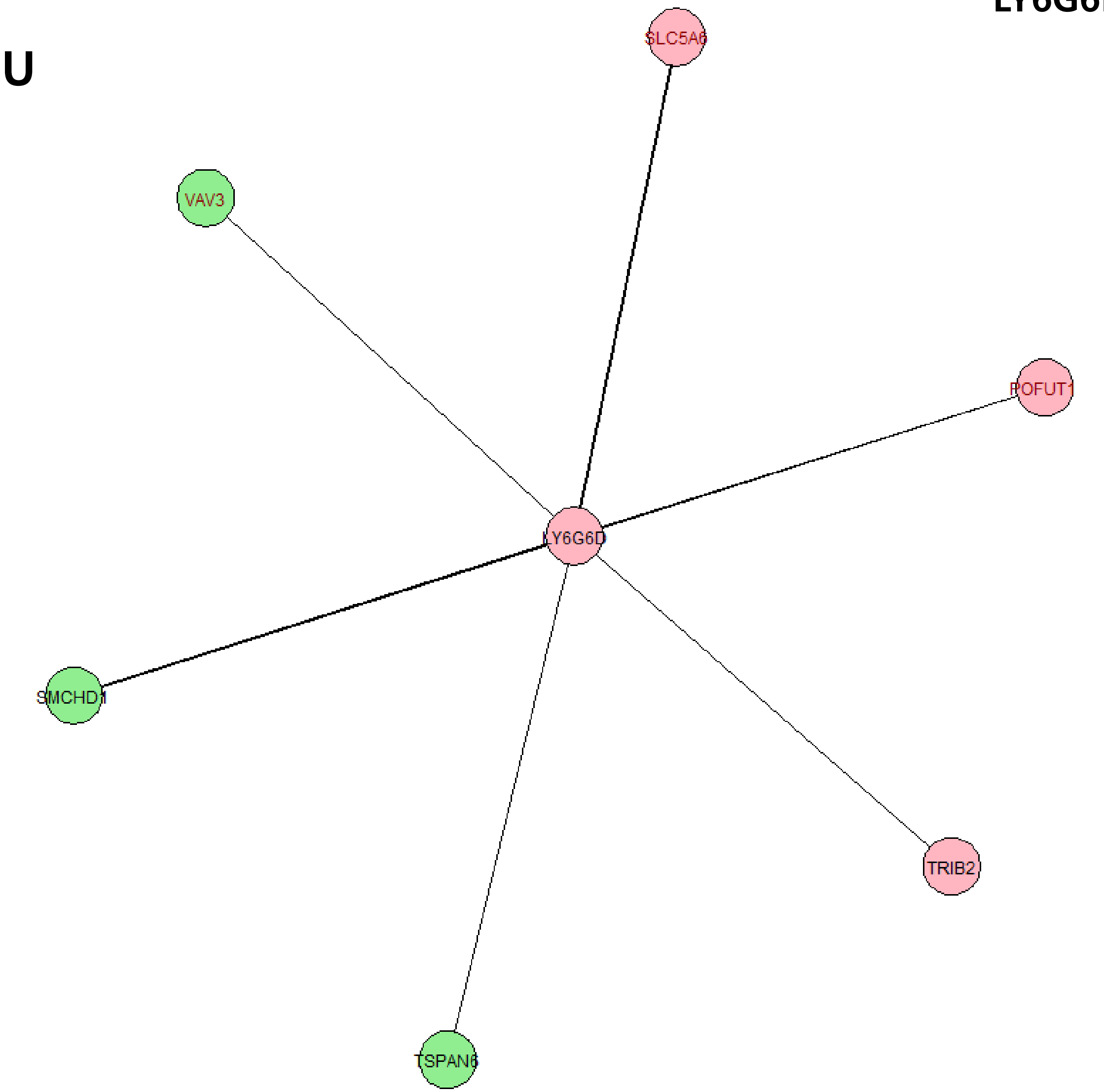

Edges from Hub of LN(+)

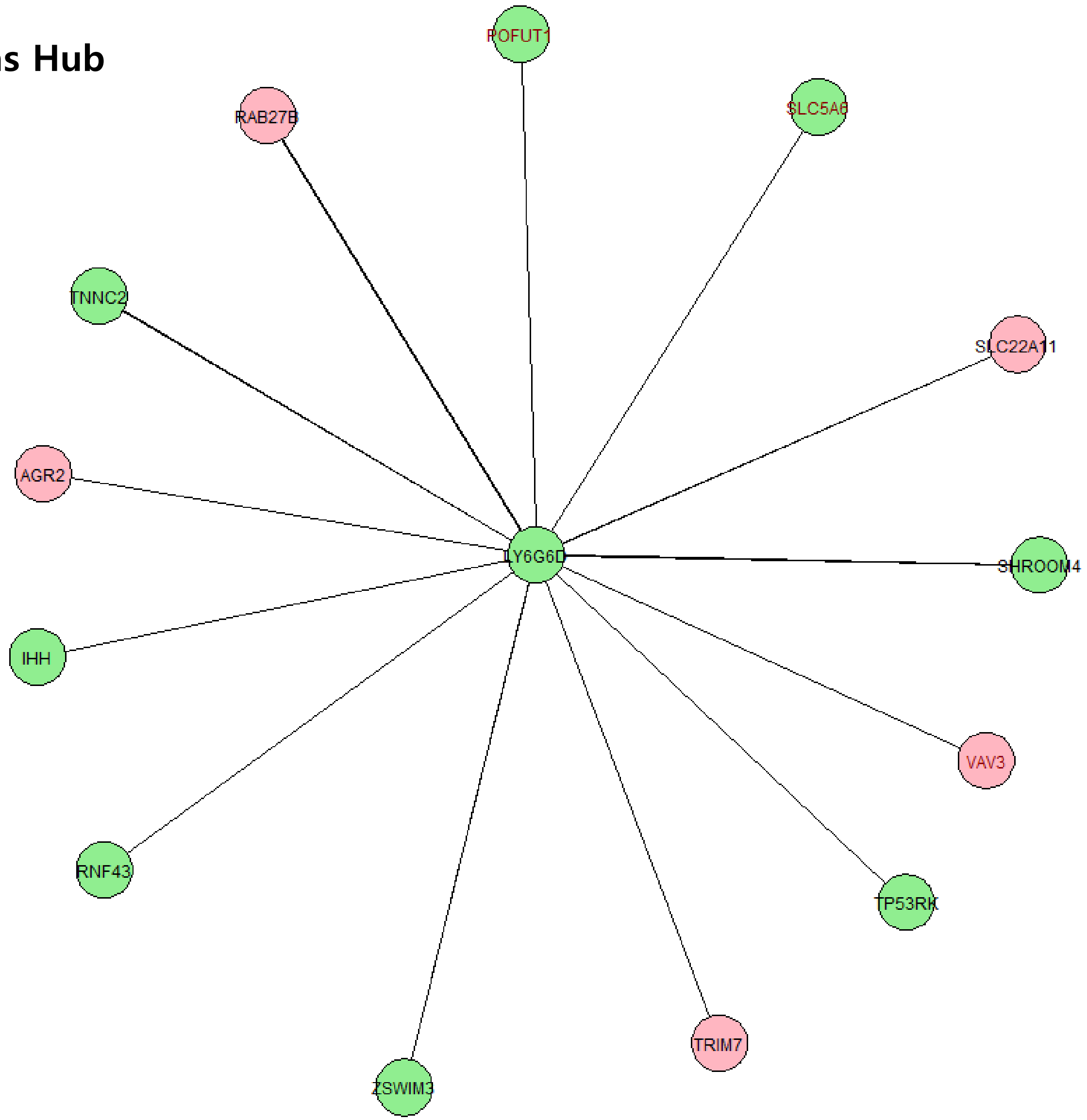

Edges from Hub of LN(-)

SSB as Hub

V

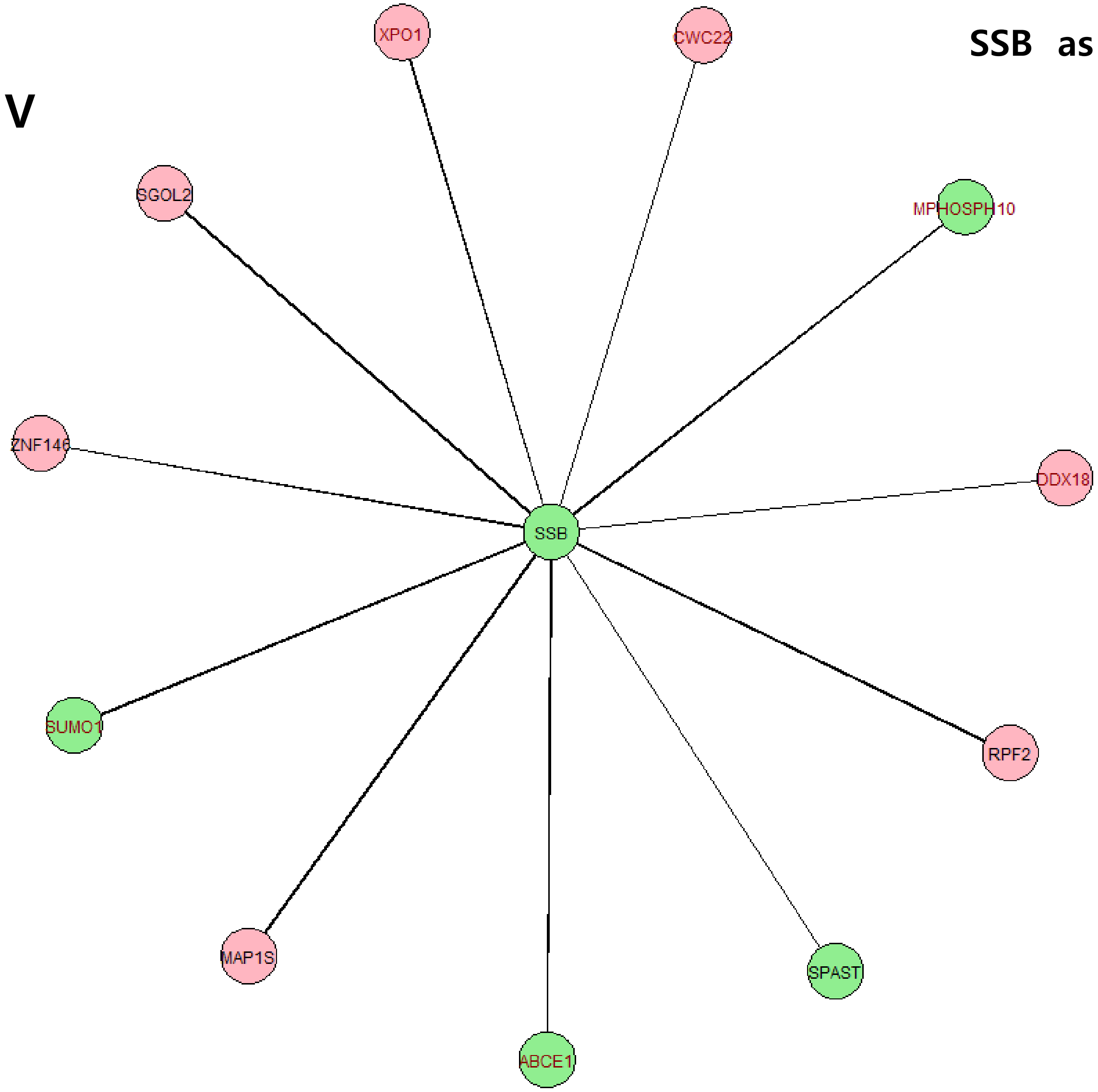

Edges from Hub of LN(+)

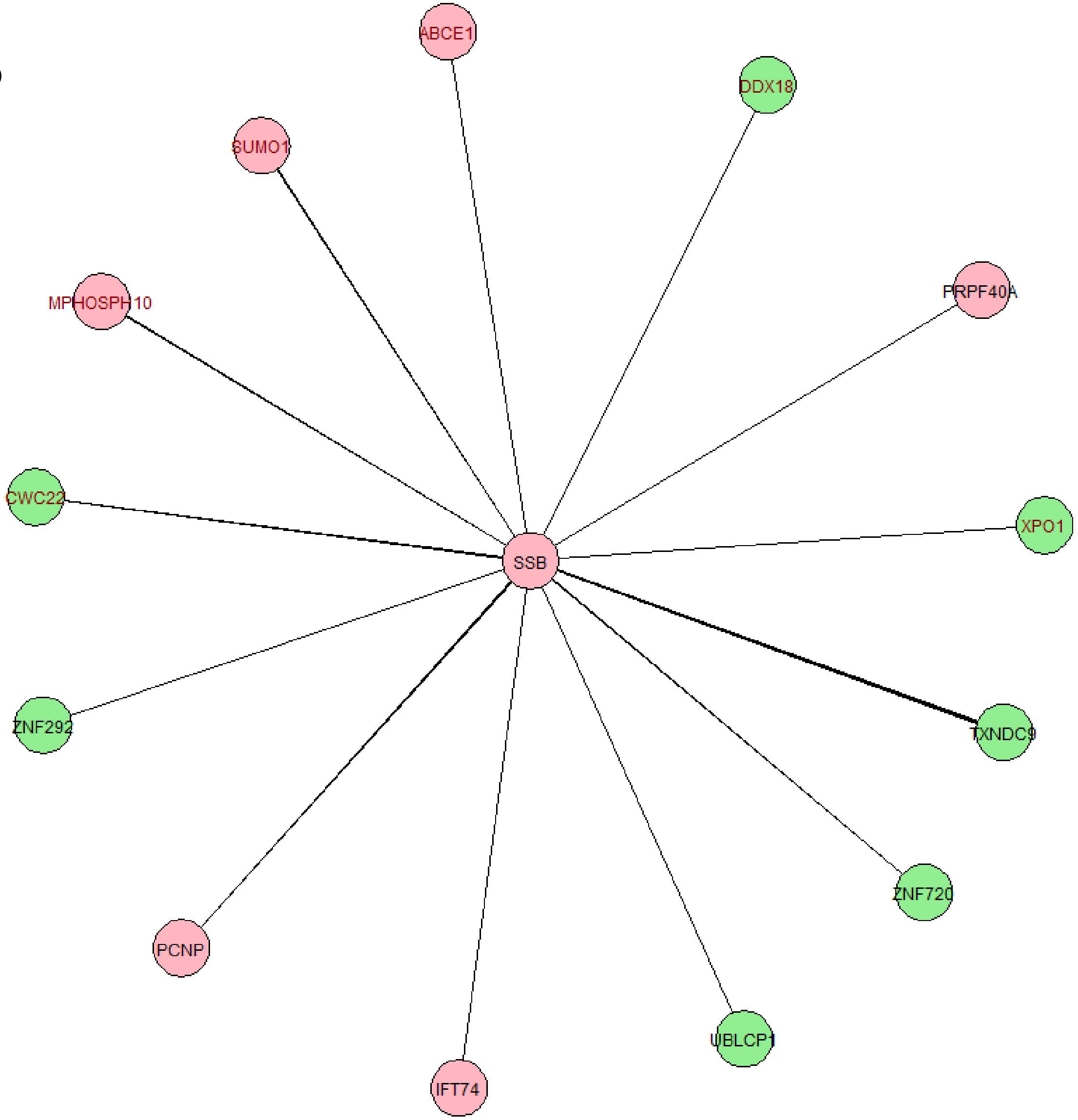

Edges from Hub of LN(-)

Supplementary Figure 5. Degree of centrality analysis of the top 353 hub genes in the LN(-) group. A. Three hundred and fifty-three hub genes identified in the LN(-) group B. Three hundred and fifty-three hub genes identified in the LN(+) group. The location of each gene in A and B is identical. Green fill: downregulated genes in the DEG analysis, Red fill: upregulated genes in the DEG analysis, Red font: common genes in both groups, Edge width: coefficient power

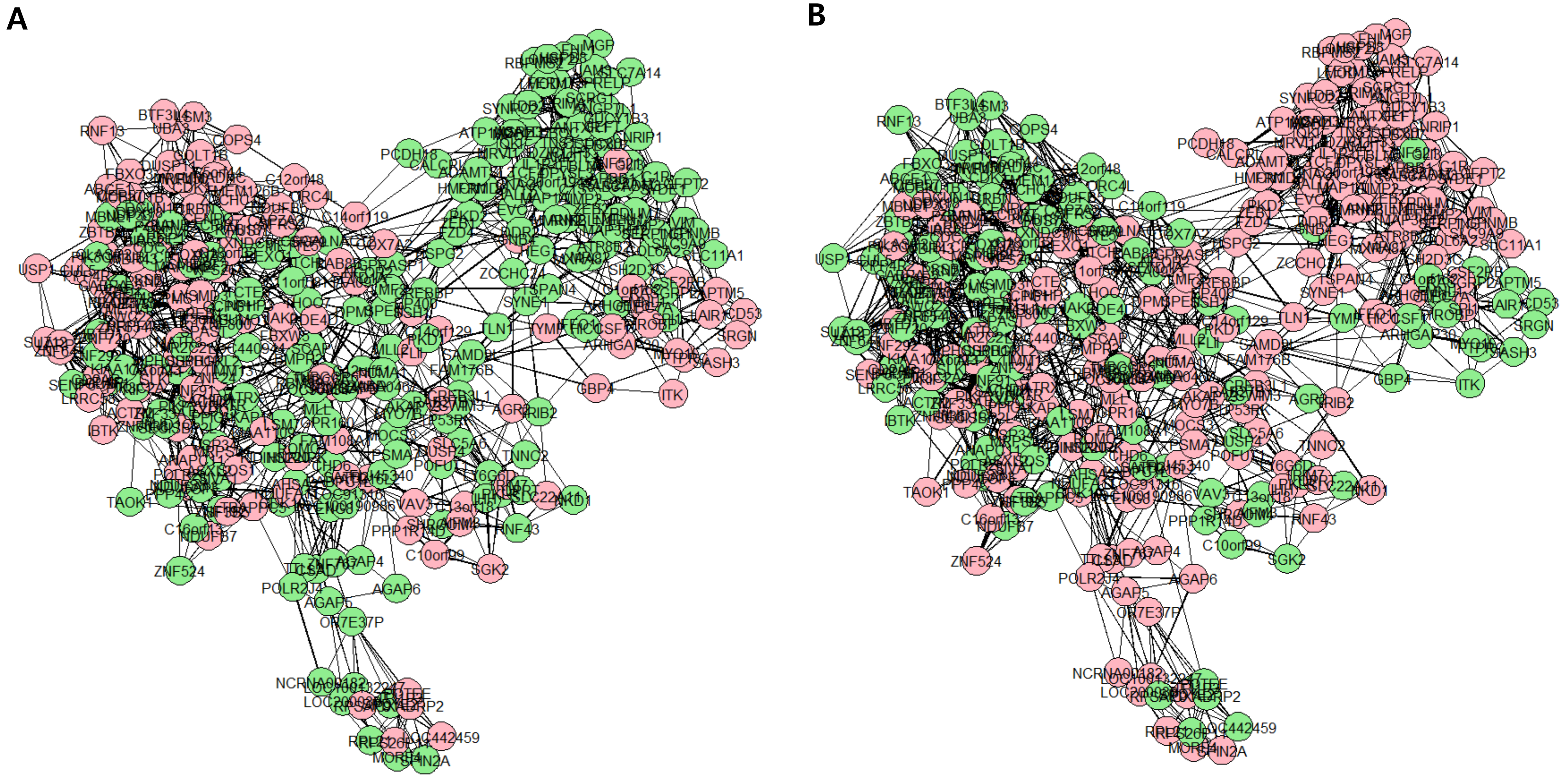

Supplementary Figure 6. Degree of centrality analysis of 127 common hub genes in the LN(+) and LN(-) group. A. One hundred and twenty-seven common hub genes identified in LN(+) B. One hundred and twenty-seven common hub genes identified in LN(-). Green fill: down-regulated genes in the DEG analysis, Red fill: up-regulated genes in the DEG analysis, Red font: common genes in both groups, Edge width: coefficient power.

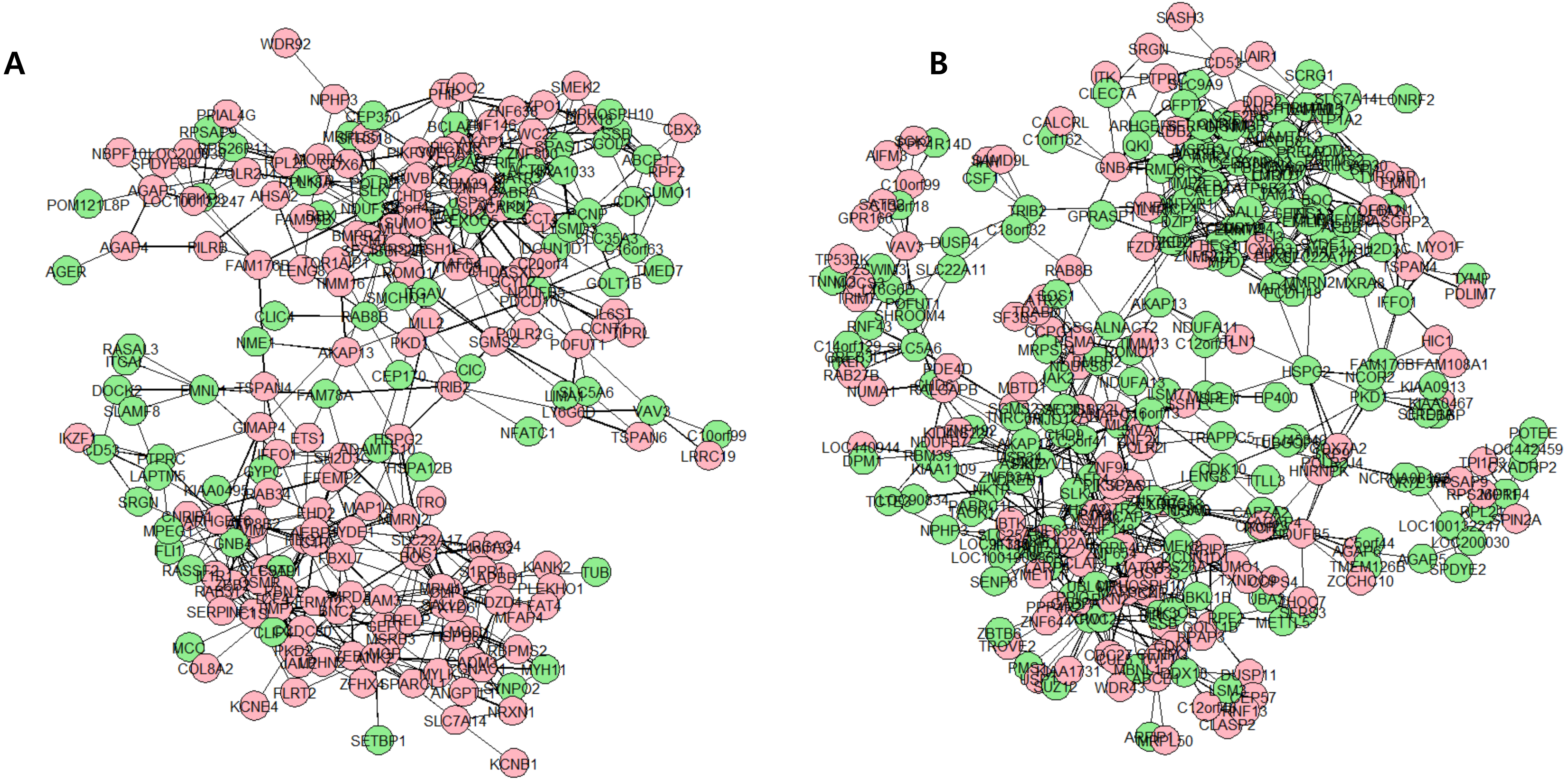

Supplementary Figure 7. Kaplan-Meier survival curve of the selected hub genes (addition to Figure 5). A. DEG/LN(+)/LN(-) B. DEG/LN(+) C. DEG/LN(-) D. Selected hub of hub genes with a high degree

A

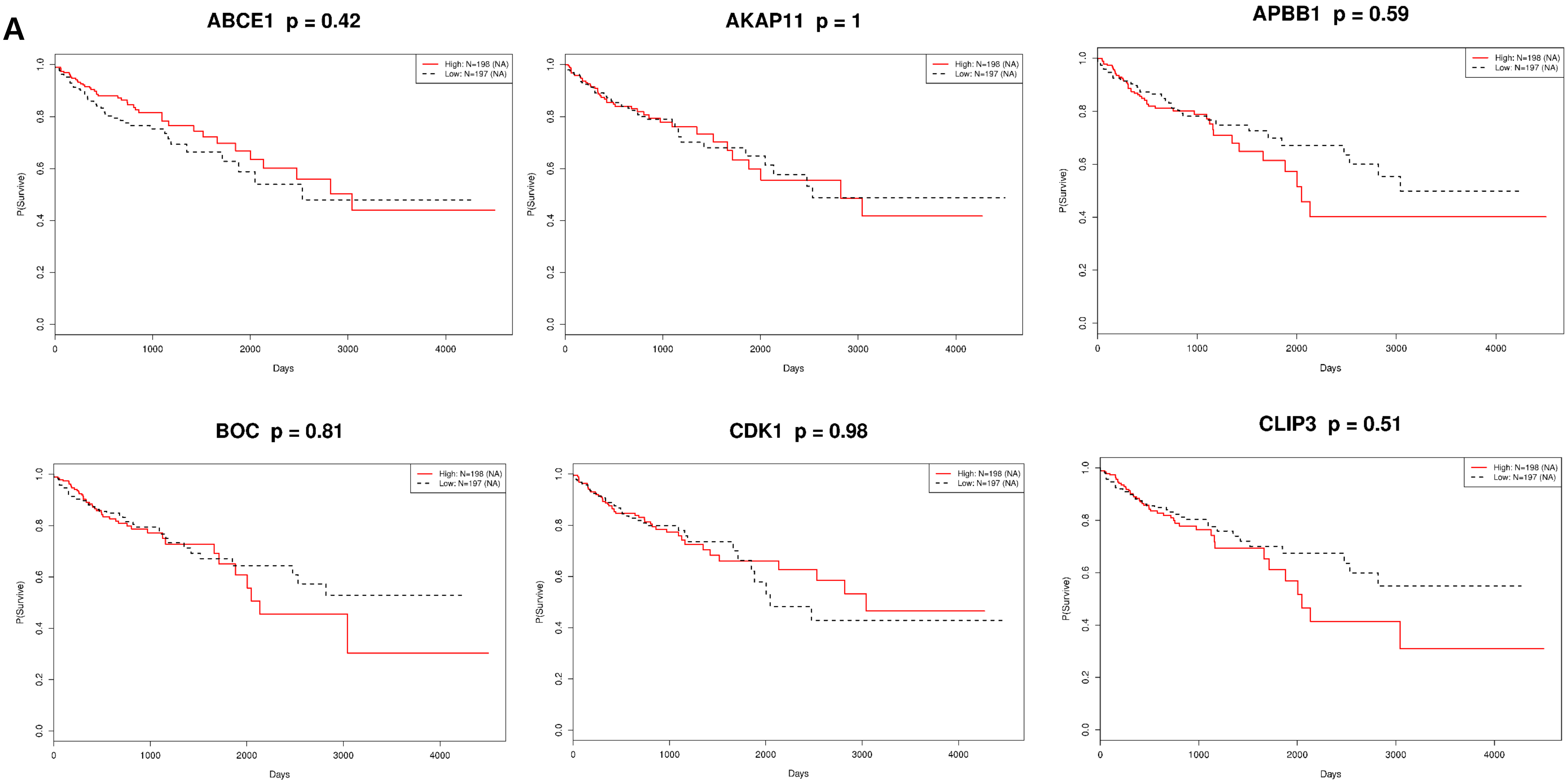

A

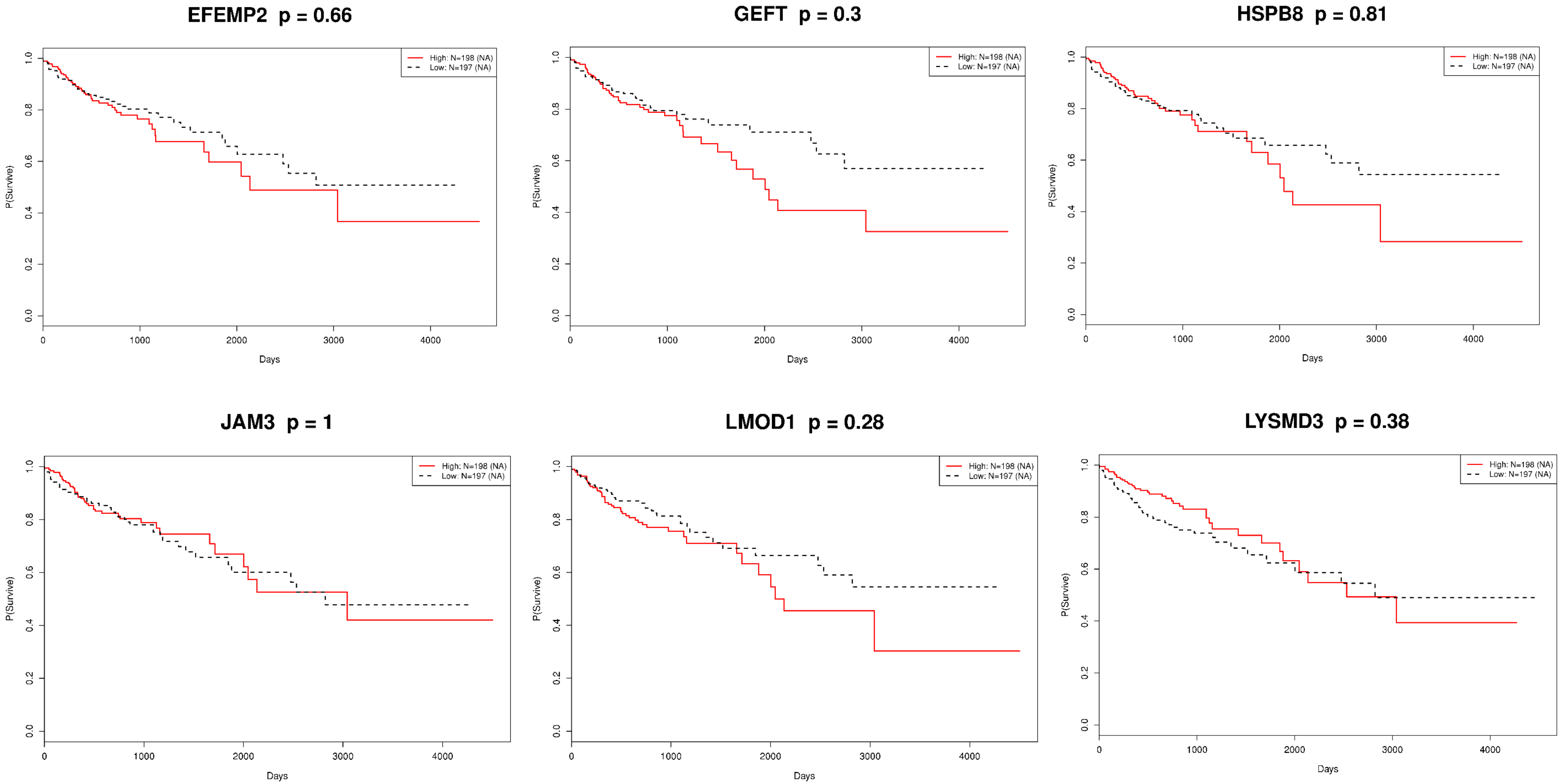
EFEMP2 p = 0.66
GEFT p = 0.3
HSPB8 p = 0.81
JAM3 p = 1
LMOD1 p = 0.28
LYSMD3 p = 0.38
High: N=198 (NA)
Low: N=197 (NA)
P(Survive)
Days
0.0
0.2
0.4
0.6
0.8
1.0
0
1000
2000
3000
4000

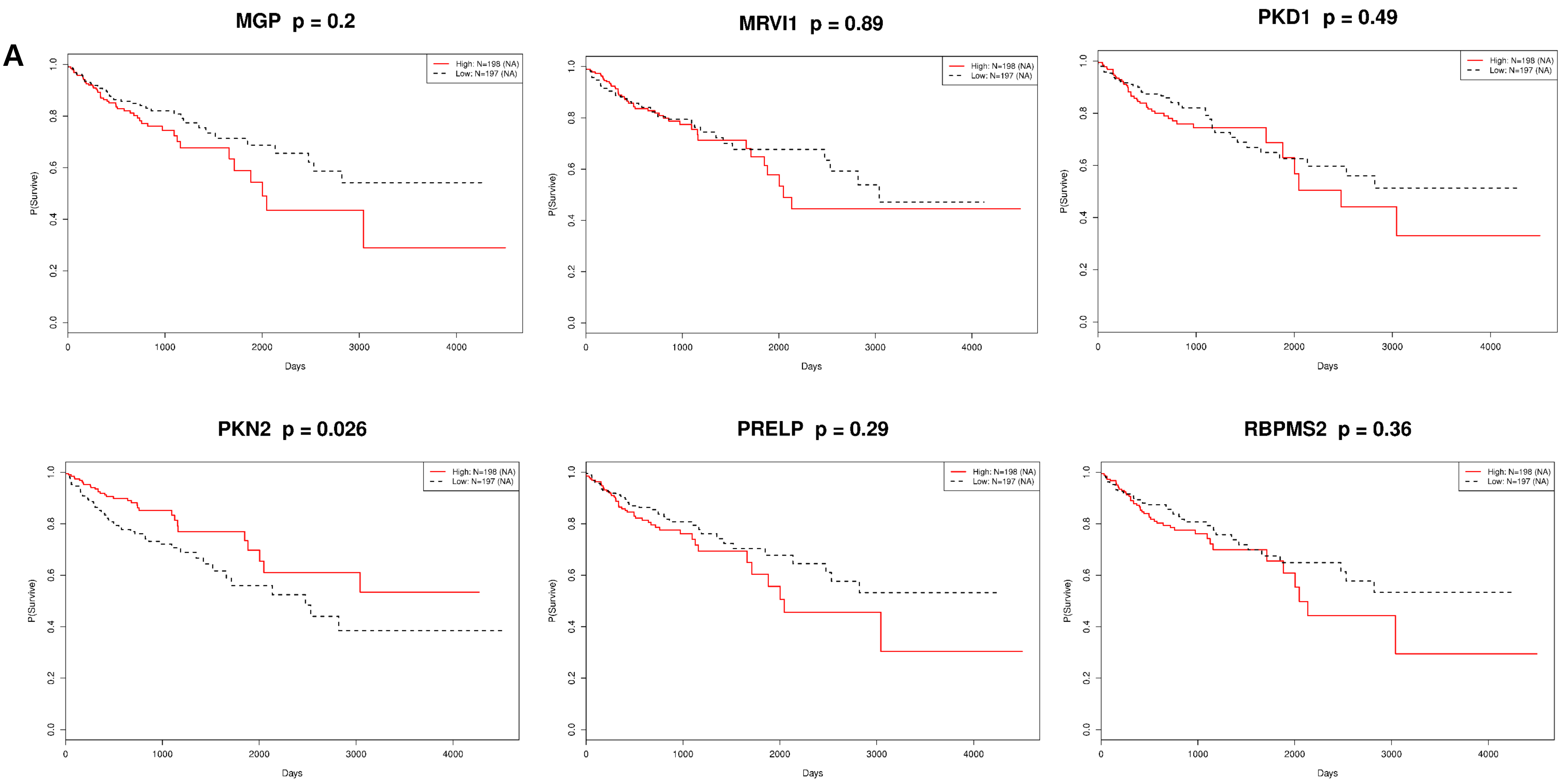
A
MGP p = 0.2
MRVI1 p = 0.89
PKD1 p = 0.49
PKN2 p = 0.026
PRELP p = 0.29
RBPMS2 p = 0.36
High: N=198 (NA)
Low: N=197 (NA)
P(Survive)
Days

A

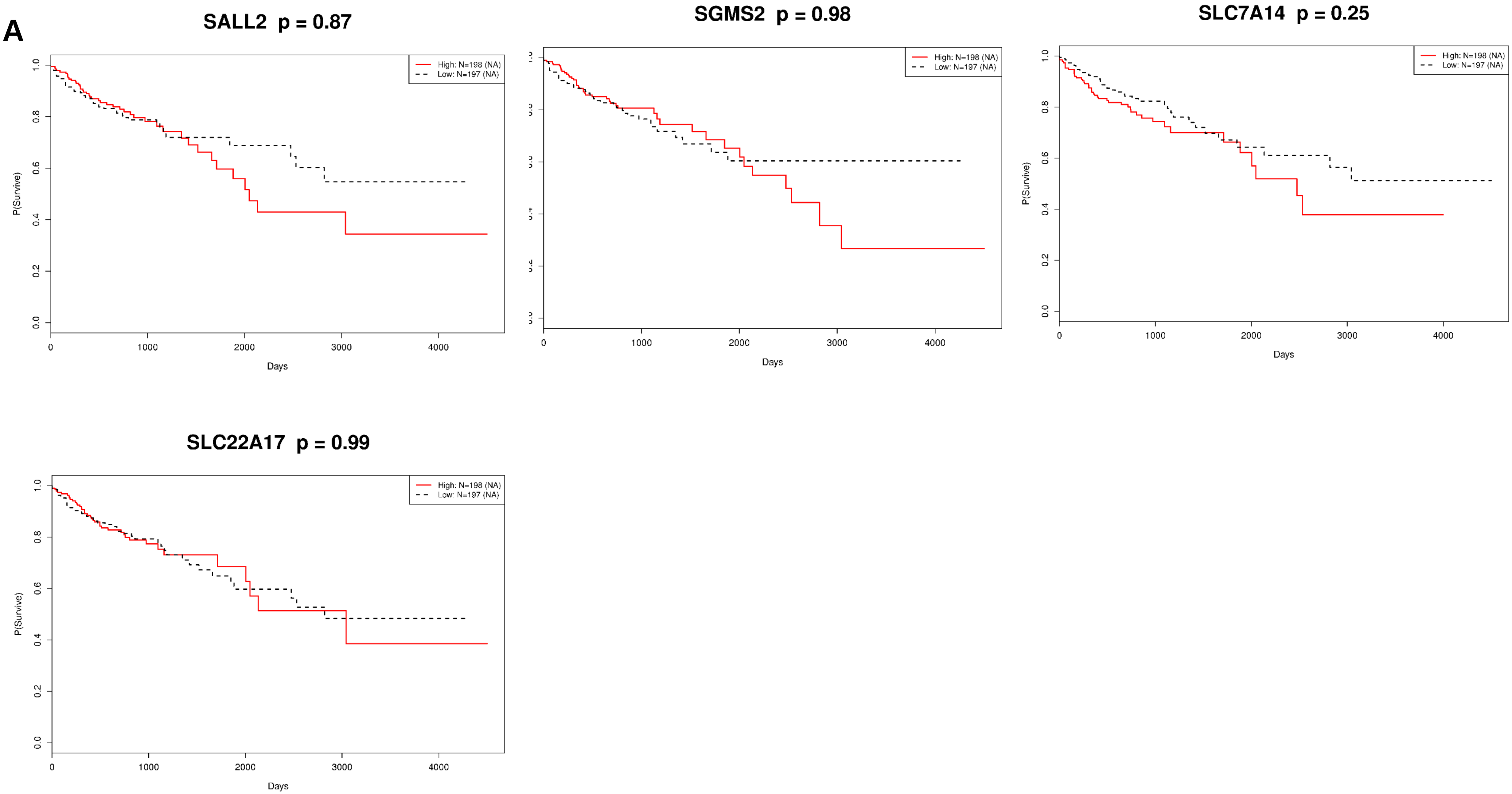

SALL2 p = 0.87
SGMS2 p = 0.98
SLC7A14 p = 0.25
SLC22A17 p = 0.99
High: N=198 (NA)
Low: N=197 (NA)
P(Survive)
Days
0
1000
2000
3000
4000
0.0
0.2
0.4
0.6
0.8
1.0

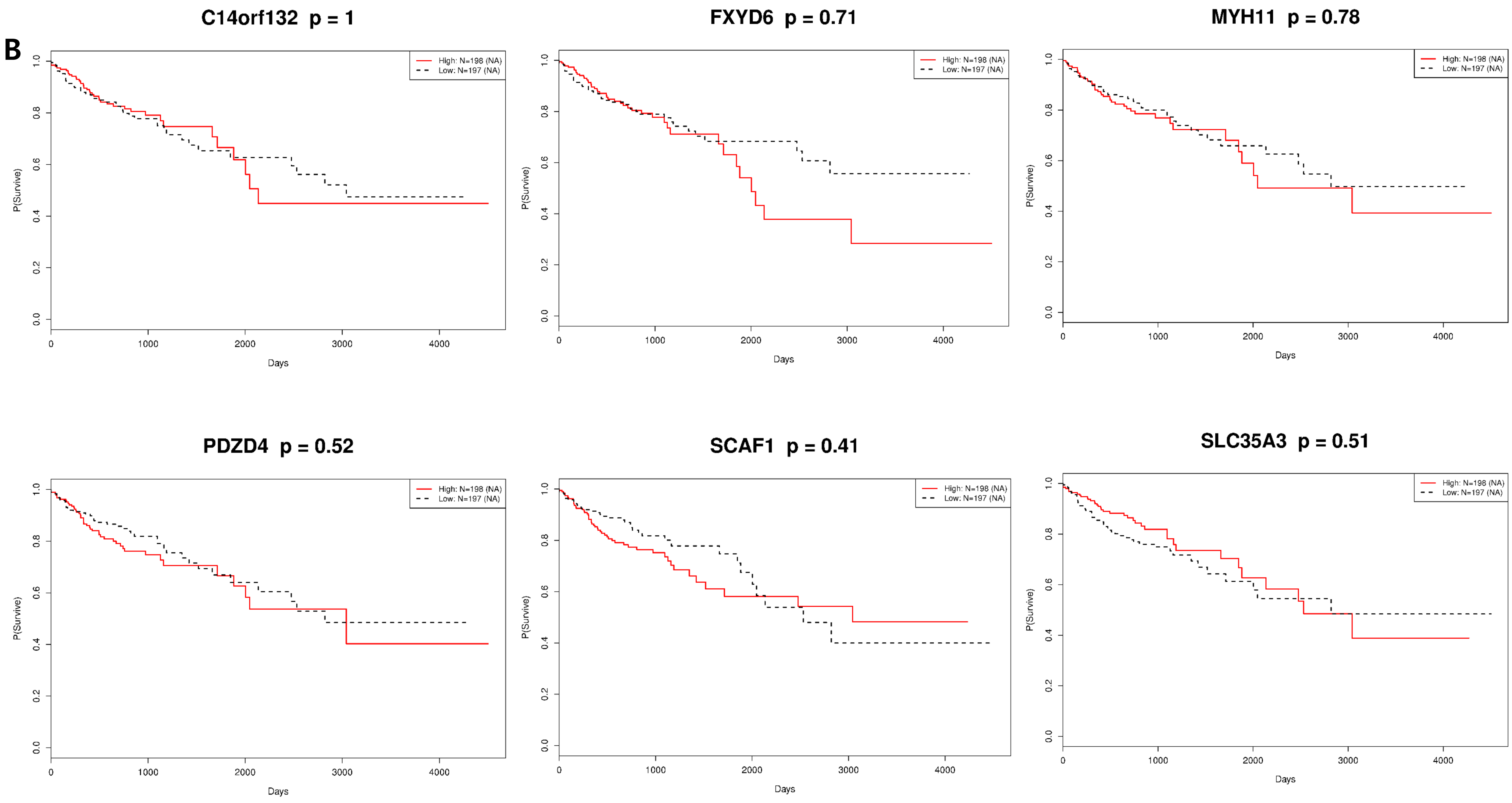

B
C14orf132 p = 1
FXYD6 p = 0.71
MYH11 p = 0.78
PDZD4 p = 0.52
SCAF1 p = 0.41
SLC35A3 p = 0.51
High: N=198 (NA)
Low: N=197 (NA)
P(Survive)
Days

B

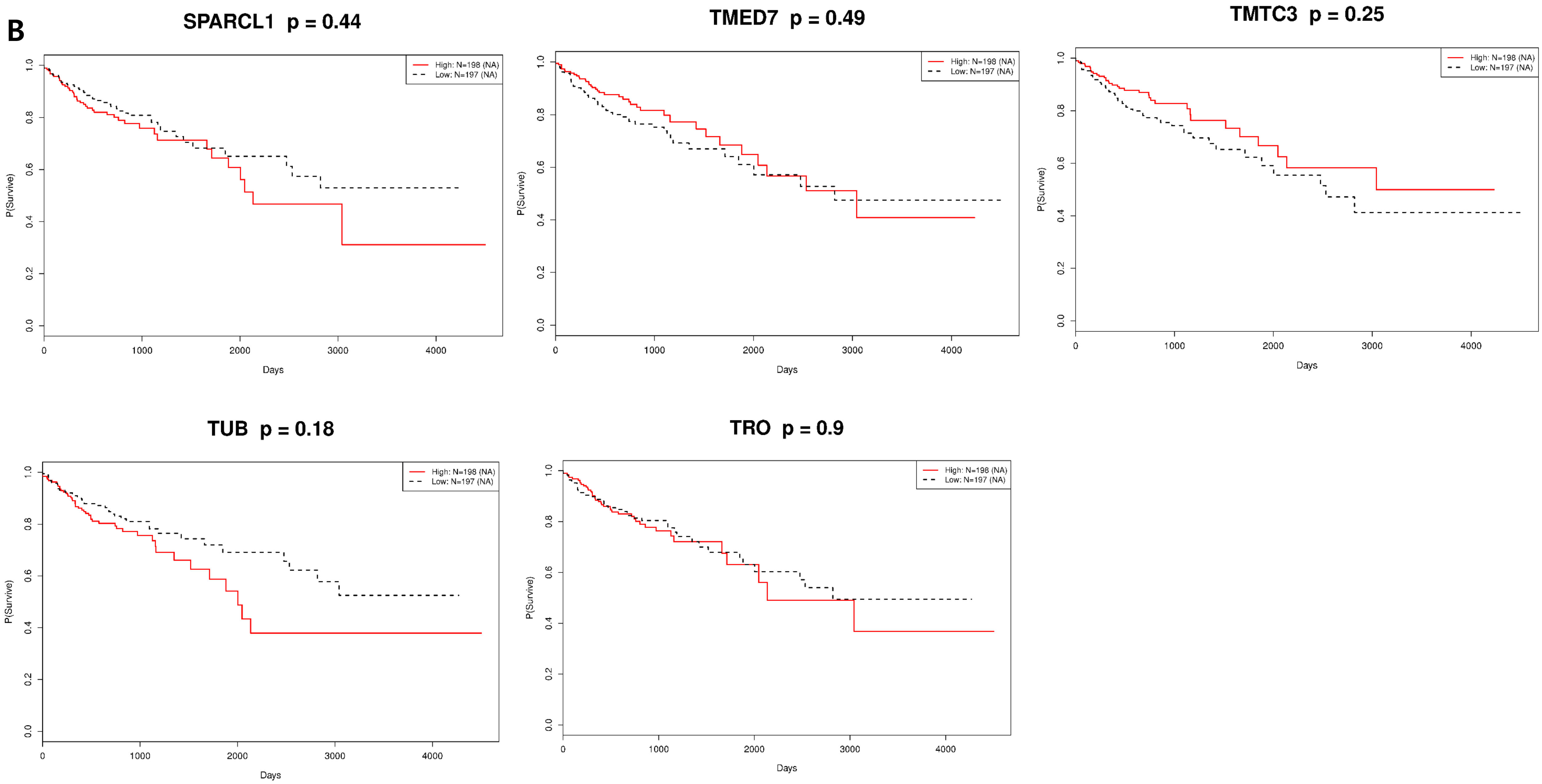
SPARCL1 p = 0.44
TMED7 p = 0.49
TMTC3 p = 0.25
TUB p = 0.18
TRO p = 0.9
High: N=198 (NA)
Low: N=197 (NA)
P(Survive)
Days

C

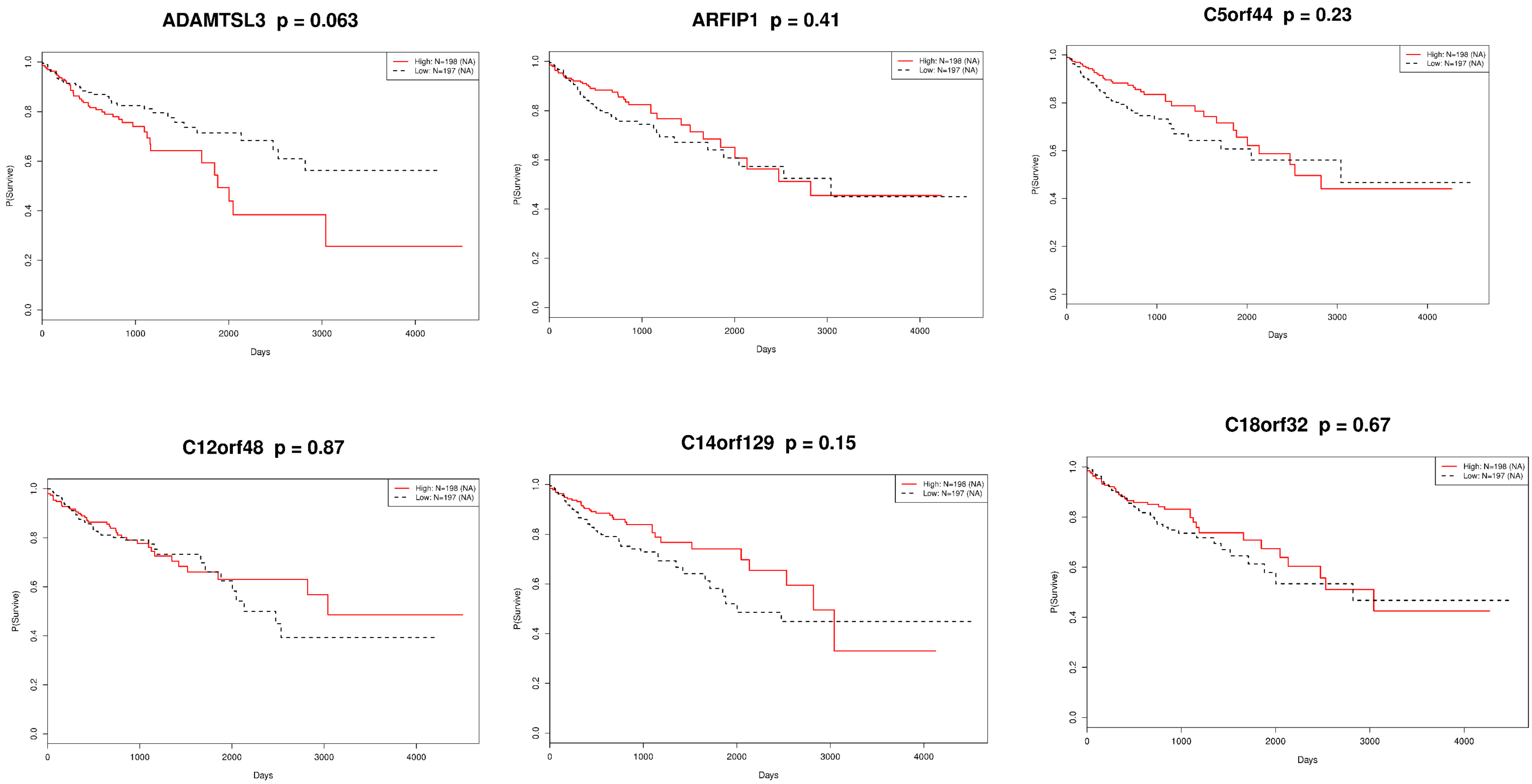

ADAMTSL3 p = 0.063
ARFIP1 p = 0.41
C5orf44 p = 0.23
C12orf48 p = 0.87
C14orf129 p = 0.15
C18orf32 p = 0.67
High: N=198 (NA)
Low: N=197 (NA)
P(Survive)
Days
0.0
0.2
0.4
0.6
0.8
1.0
0
1000
2000
3000
4000

C

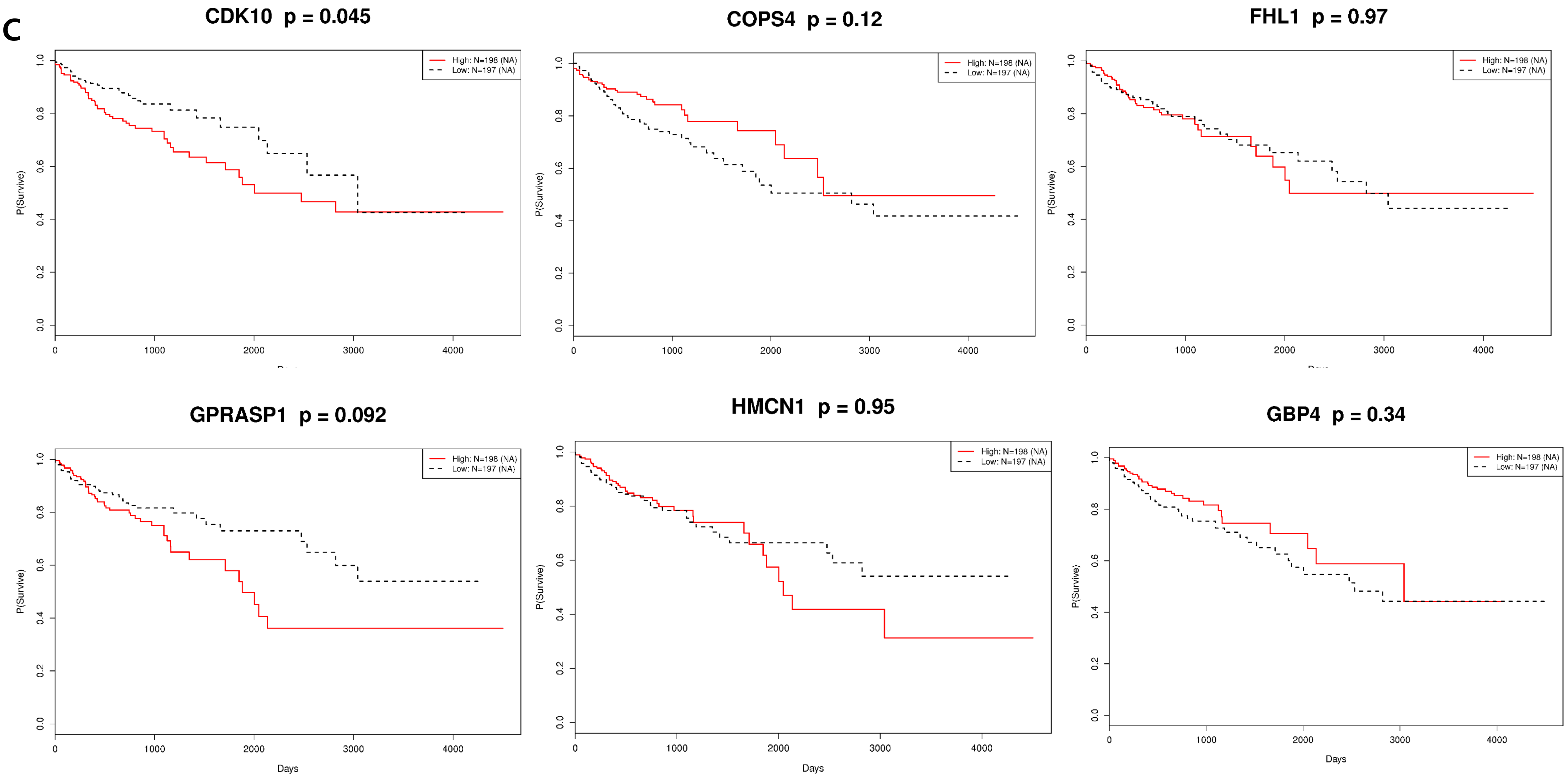
CDK10 p = 0.045
COPS4 p = 0.12
FHL1 p = 0.97
GPRASP1 p = 0.092
HMCN1 p = 0.95
GBP4 p = 0.34
High: N=198 (NA)
Low: N=197 (NA)
P(Survive)
Days

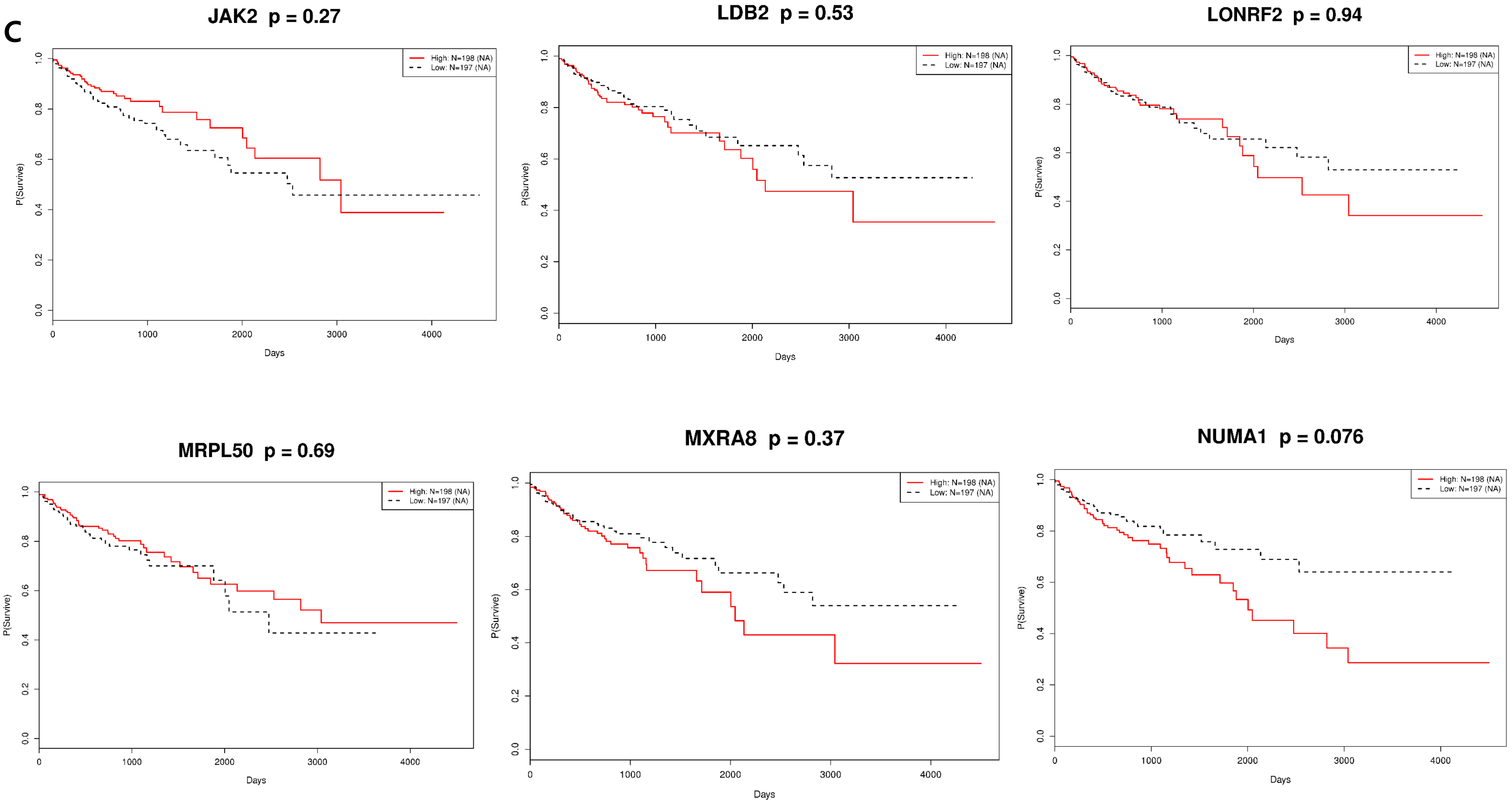
C
JAK2 p = 0.27
LDB2 p = 0.53
LONRF2 p = 0.94
MRPL50 p = 0.69
MXRA8 p = 0.37
NUMA1 p = 0.076
High: N=198 (NA)
Low: N=197 (NA)
P(Survive)
Days
0.0
0.2
0.4
0.6
0.8
1.0
0
1000
2000
3000
4000

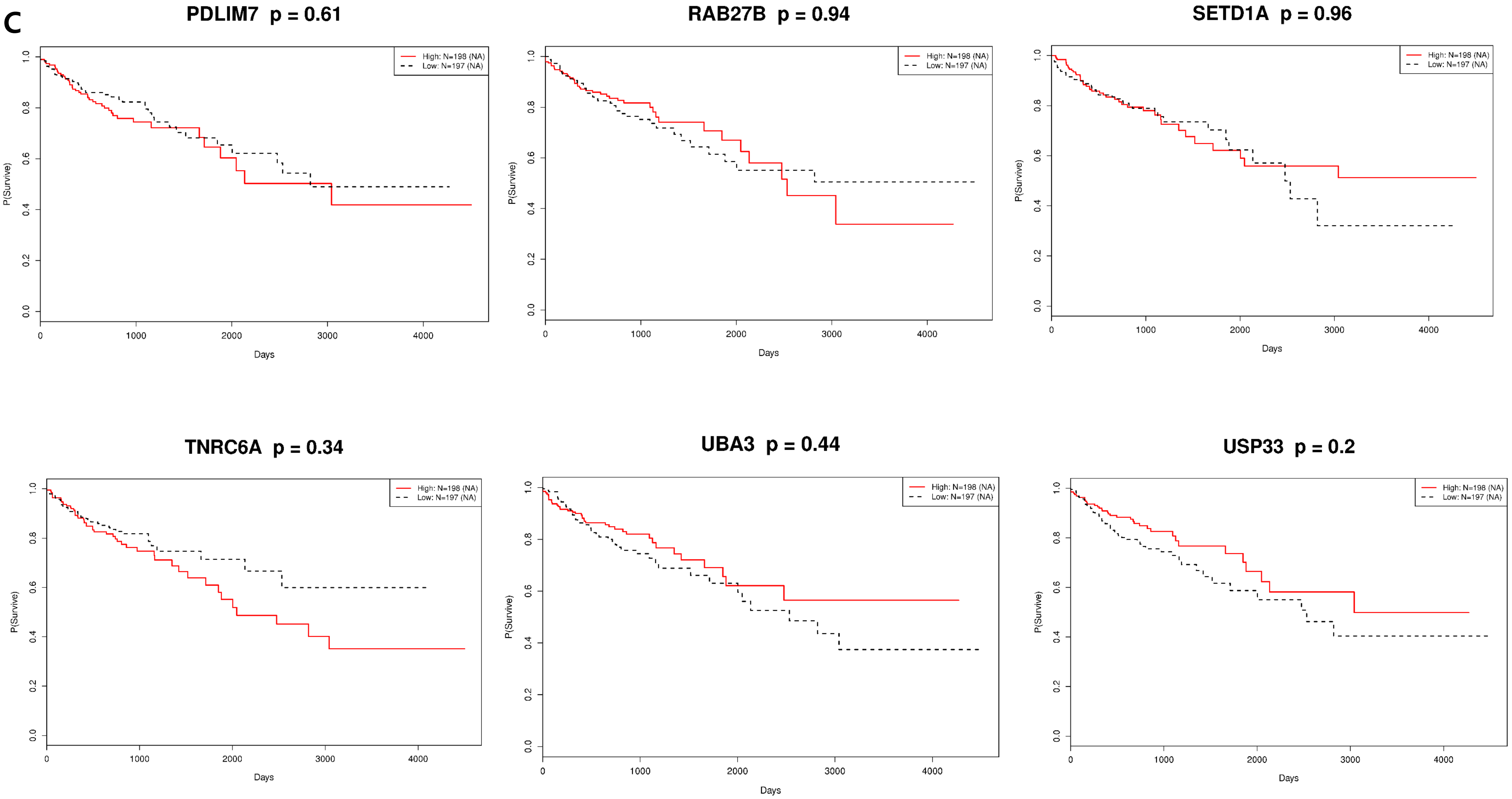
C
PDLIM7 p = 0.61
RAB27B p = 0.94
SETD1A p = 0.96
TNRC6A p = 0.34
UBA3 p = 0.44
USP33 p = 0.2
High: N=198 (NA)
Low: N=197 (NA)
P(Survive)
Days
0.0
0.2
0.4
0.6
0.8
1.0
0
1000
2000
3000
4000

C

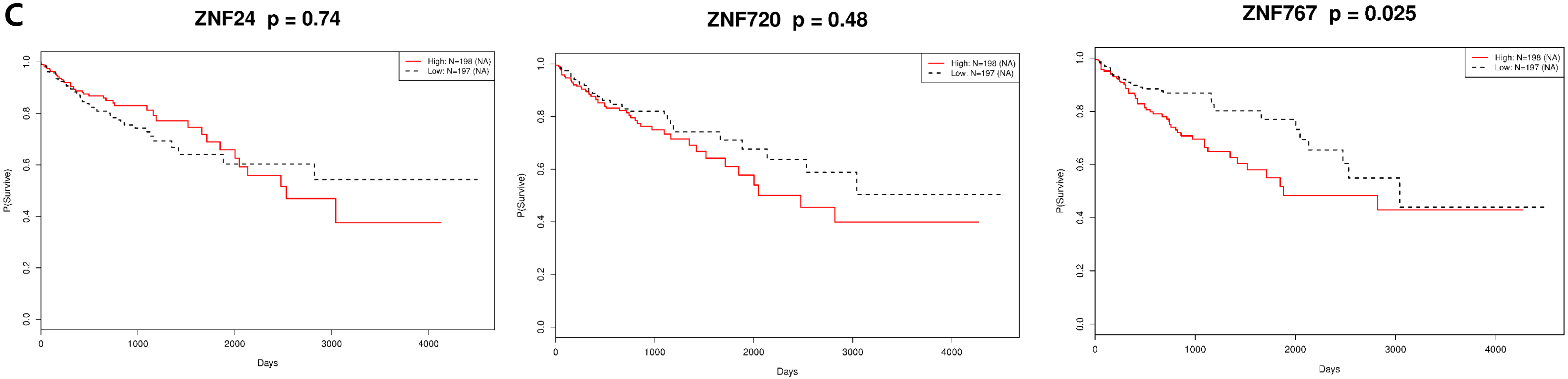

ZNF24 p = 0.74
High: N=198 (NA)
Low: N=197 (NA)
P(Survive)
1.0
0.8
0.6
0.4
0.2
0.0
0
1000
2000
3000
4000
Days
ZNF720 p = 0.48
High: N=198 (NA)
Low: N=197 (NA)
P(Survive)
1.0
0.8
0.6
0.4
0.2
0.0
0
1000
2000
3000
4000
Days
ZNF767 p = 0.025
High: N=198 (NA)
Low: N=197 (NA)
P(Survive)
1.0
0.8
0.6
0.4
0.2
0.0
0
1000
2000
3000
4000
Days

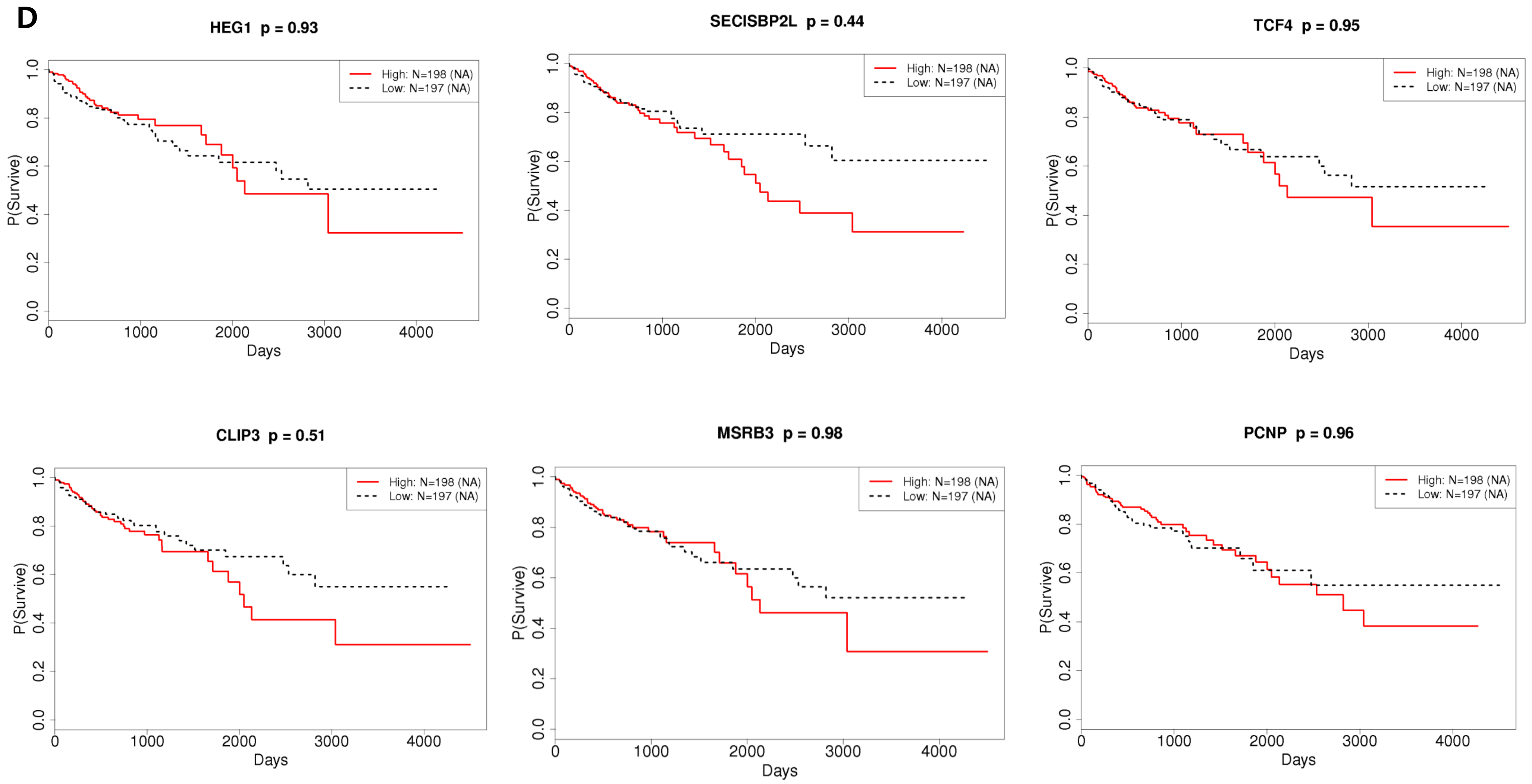

D
HEG1 p = 0.93
SECISBP2L p = 0.44
TCF4 p = 0.95
CLIP3 p = 0.51
MSRB3 p = 0.98
PCNP p = 0.96
High: N=198 (NA)
Low: N=197 (NA)
P(Survive)
Days
0.0
0.2
0.4
0.6
0.8
1.0
0
1000
2000
3000
4000

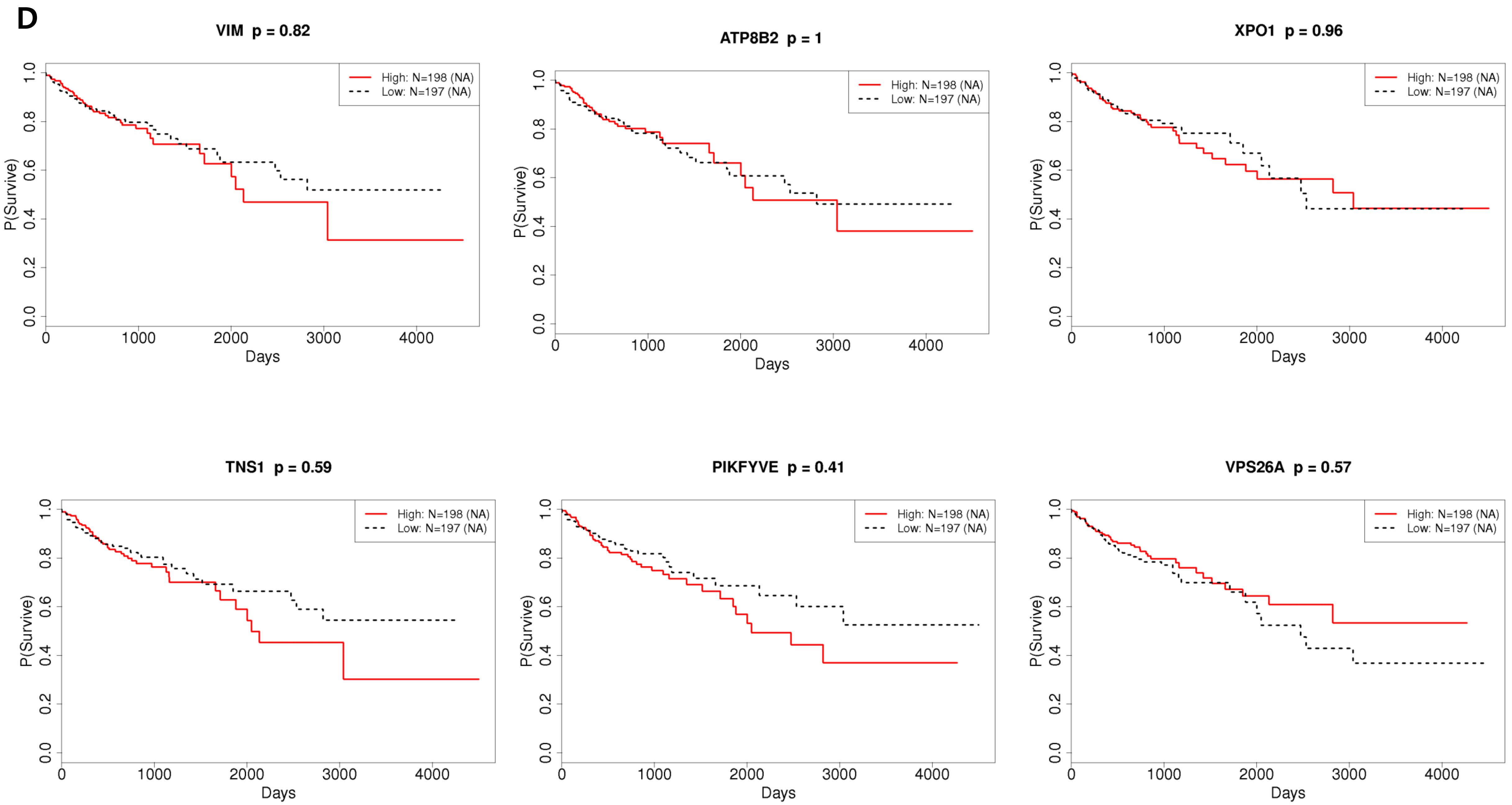
D
VIM p = 0.82
ATP8B2 p = 1
XPO1 p = 0.96
TNS1 p = 0.59
PIKFYVE p = 0.41
VPS26A p = 0.57
High: N=198 (NA)
Low: N=197 (NA)
P(Survive)
Days
0.0
0.2
0.4
0.6
0.8
1.0
0
1000
2000
3000
4000

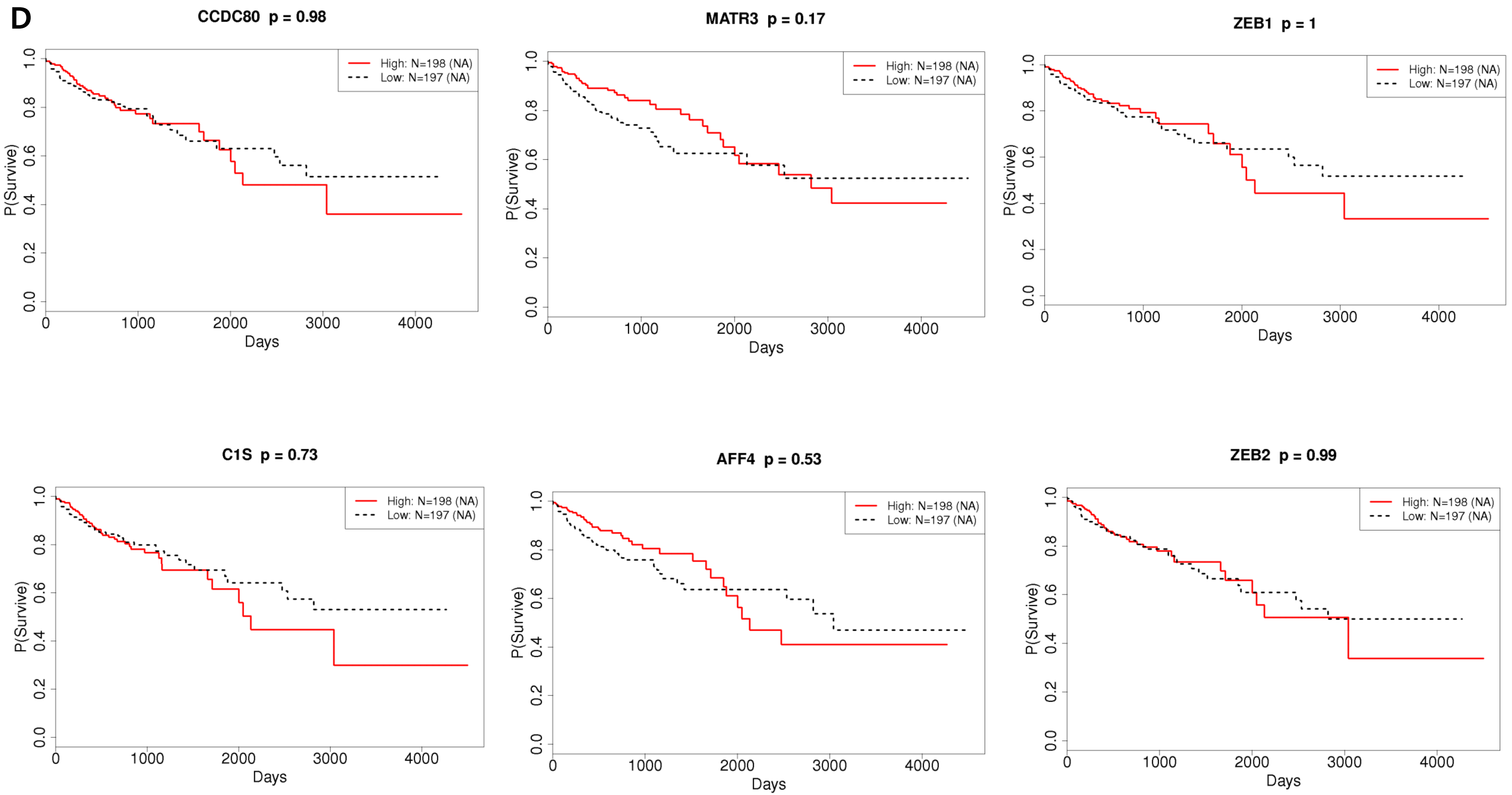

D
CCDC80 p = 0.98
MATR3 p = 0.17
ZEB1 p = 1
C1S p = 0.73
AFF4 p = 0.53
ZEB2 p = 0.99
High: N=198 (NA)
Low: N=197 (NA)
P(Survive)
0.0
0.2
0.4
0.6
0.8
1.0
Days
0
1000
2000
3000
4000

D

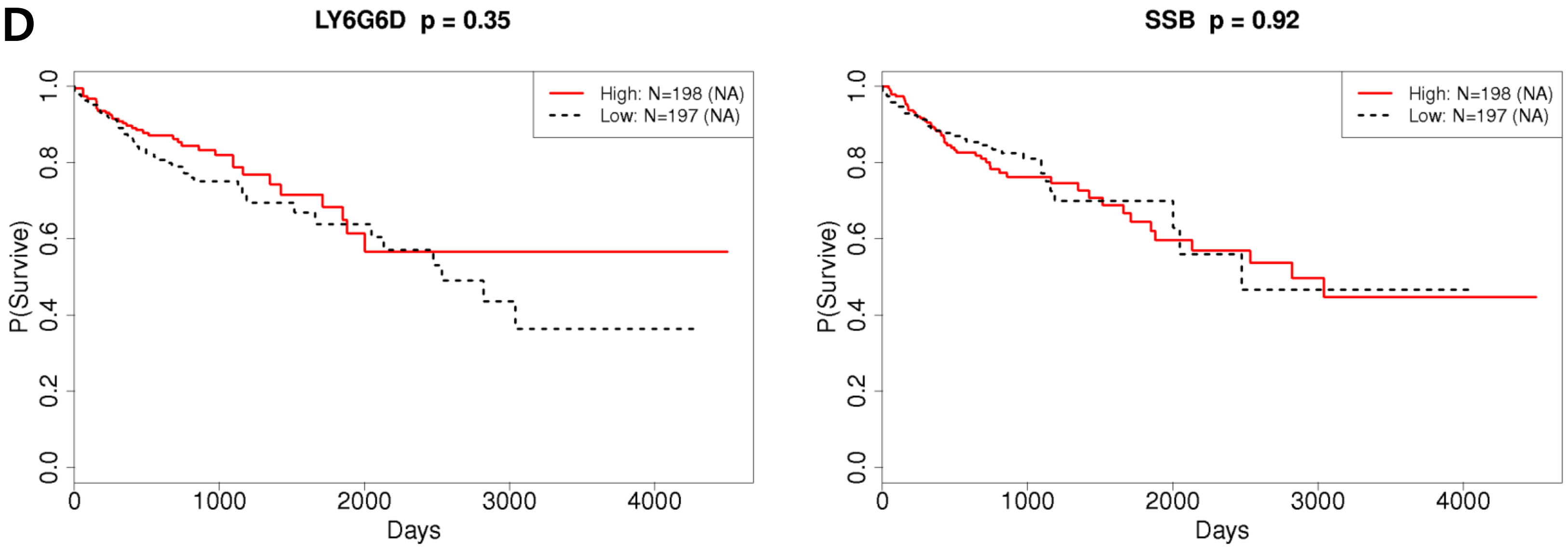
LY6G6D p = 0.35
SSB p = 0.92
High: N=198 (NA)
Low: N=197 (NA)
P(Survive)
Days
0
1000
2000
3000
4000
0.0
0.2
0.4
0.6
0.8
1.0
